# Diagnosing Linearity Along the Carbon Cascade in Terrestrial Biosphere Models and Observations

**DOI:** 10.1111/gcb.70982

**Published:** 2026-07-26

**Authors:** Huanyuan Zhang‐Zheng, Vivek K. Arora, Peter Anthoni, Thomas A. M. Pugh, Atul K. Jain, Wenping Yuan, Yadvinder Malhi, Julia Nabel, Daniel S. Goll, Julia Pongratz, Benjamin Poulter, Anthony P. Walker, Sönke Zaehle, Jürgen Knauer, Etsushi Kato, Ruijie Ding, Minxue Tang, Stephen Sitch, Michael O'Sullivan, César Terrer, Hanqin Tian, Naiqing Pan, Pierre Friedlingstein, Akihiko Ito, Qing Sun, Jeanne Decayeux, Benjamin D. Stocker

**Affiliations:** ^1^ Environmental Change Institute, School of Geography and the Environment University of Oxford Oxford UK; ^2^ Leverhulme Centre for Nature Recovery University of Oxford Oxford UK; ^3^ Department of Ecology, Environment and Geoscience Umeå University Umeå Sweden; ^4^ Canadian Centre for Climate Modelling and Analysis Environment and Climate Change Canada Victoria Canada; ^5^ Institute of Meteorology and Climate Research/Atmospheric Environmental Research Karlsruhe Institute of Technology Garmisch‐Partenkirchen Germany; ^6^ Department of Physical Geography and Ecosystem Science Lund University Lund Sweden; ^7^ Department of Geography, Earth and Environmental Sciences University of Birmingham Birmingham UK; ^8^ Birmingham Institute of Forest Research University of Birmingham Birmingham UK; ^9^ Department of Climate, Meteorology, and Atmospheric Sciences (CLiMAS) University of Illinois Urbana‐Champaign Urbana Illinois USA; ^10^ Institute of Carbon Neutrality, Sino‐French Institute for Earth System Science, College of Urban and Environmental Sciences Peking University Beijing China; ^11^ Max Planck Institute for Biogeochemistry Jena Germany; ^12^ Max Planck Institute for Meteorology Hamburg Germany; ^13^ Laboratoire Des Sciences du Climat et de L'environnement CEA‐CNRS‐UVSQ‐Université Paris‐Saclay Gif‐sur‐Yvette France; ^14^ Department of Geography Ludwig‐Maximilians‐Universität München München Germany; ^15^ Spark Climate Solutions San Francisco California USA; ^16^ Department of Geographical Sciences University of Maryland College Park Maryland USA; ^17^ Environmental Sciences Division and Climate Change Science Institute Oak Ridge National Laboratory Oak Ridge Tennessee USA; ^18^ School of Life Sciences, Faculty of Science University of Technology Sydney Ultimo New South Wales Australia; ^19^ Hawkesbury Institute for the Environment Western Sydney University Penrith New South Wales Australia; ^20^ Institute of Applied Energy Tokyo Japan; ^21^ Georgina Mace Centre for the Living Planet, Department of Life Sciences Imperial College London Ascot UK; ^22^ Faculty of Environment, Science and Economy University of Exeter Exeter UK; ^23^ Department of Civil and Environmental Engineering Massachusetts Institute of Technology Cambridge Massachusetts USA; ^24^ Center for Earth System Science and Global Sustainability, Schiller Institute for Integrated Science and Society Boston College Chestnut Hill Massachusetts USA; ^25^ Department of Earth and Environmental Sciences Boston College Chestnut Hill Massachusetts USA; ^26^ Laboratoire de Météorologie Dynamique, Institut Pierre‐Simon Laplace, CNRS, École Normale Supérieure Université PSL, Sorbonne Université, École Polytechnique Paris France; ^27^ Graduate School of Agricultural and Life Sciences The University of Tokyo Tokyo Japan; ^28^ Climate and Environmental Physics, Physics Institute University of Bern Bern Switzerland; ^29^ Wyss Academy for Nature University of Bern Bern Switzerland; ^30^ Oeschger Center for Climate Change Research University of Bern Bern Switzerland; ^31^ Centre National de Recherches Météorologiques (CNRM), Météo‐France, CNRS Université de Toulouse Toulouse France; ^32^ Institute of Geography University of Bern Bern Switzerland; ^33^ Oeschger Centre for Climate Change Research University of Bern Bern Switzerland

**Keywords:** carbon allocation, carbon cycle, elevated CO_2_, gross primary production, net primary production, vegetation models

## Abstract

Elevated carbon dioxide (eCO_2_) acts as a fertiliser for photosynthesis, driving an increase in gross primary production (GPP). However, it is unclear how effectively increased GPP propagates along the ‘carbon (C) cascade’ to increase net primary production (NPP) and vegetation C stocks (*C*_veg_) in different plant compartments. Vegetation models were criticised for being overly sensitive to photosynthesis (source‐driven), neglecting sink‐driven processes which may attenuate (or amplify) changes in NPP and vegetation C stocks. Here, we introduce an analytical framework to diagnose linearity (*L*) as ratios of relative changes in linked fluxes and pools. We then apply this framework to 16 models of the TRENDY v11 ensemble and to observation‐based estimates of CO_2_ sensitivities. We found widely varying global patterns in *L* across models. Six models showed a majority of grid cells with larger relative changes in NPP than in GPP (*L*_NPP:GPP_ > 1 for > 60% of gridcells), indicating increased vegetation carbon use efficiency under eCO_2_. Only three models had *L*_NPP:GPP_ < 1 for > 60% of gridcells. Four models showed a majority of gridcells with larger relative changes in estimated steady‐state *C*_veg_ than in NPP, while five models showed the opposite—in both cases with a large spread of *L*_Cveg*:NPP_ across grid cells within models. Observations‐based analysis reveals median *L*_Cveg*:NPP_ < 1 overall but evidence is insufficient for a conclusion. Three models showed a larger relative increase in root C than in *C*_veg_, (*L*_Croot:Cveg_ > 1) while five models showed the opposite. Most field evidence shows *L*_Croot:Cveg_ > 1. Widely differing distributions of *L* among models and links in the C cascade reveal a strong influence of nonlinear behaviour in individual models. However, due to the spread in *L*, across the whole models ensemble, *L* deviations from 1 were roughly balanced, leading to an overall linear behaviour of terrestrial C cycle representations in the multi‐model‐mean.

## Graphical abstract

Vegetation models simulate processes at different scales, from photosynthesis (cell), gross primary production (canopy), and net primary production (whole plant) to vegetation and soil carbon storage (ecosystem). Carbon fertilisation leads to increased photosynthesis, but does this translate into enhancement of other processes? A common critique of models is that their simulations are overly determined by how photosynthesis responds to rising CO_2_ (source‐driven), while other constraints on carbon assimilation into long‐lived C pools (sink‐driven) are underappreciated. But after decades of model development, is this still true?

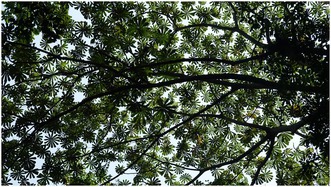

## 1 Introduction

Rising atmospheric CO_2_ has been stimulating photosynthesis of most land plants and has driven an increase in terrestrial gross primary production (GPP) by 13.5% ± 3.5% between 1981 and 2020 (Keenan et al. 2023). Over the same time period, the land biosphere has been a net carbon sink of 2.75 GtC yr.^−1^ on average (Friedlingstein et al. 2023; Pan et al. 2024; Ruehr et al. 2023), varying annually from 0.36 to 4.1 GtC yr.^−1^. This C sink partly offsets anthropogenic CO_2_ emissions and acts as a negative feedback against atmospheric CO_2_ growth, thereby slowing the pace of global warming (Canadell et al. 2021; Keenan et al. 2016). Although all organic carbon (C) stored in terrestrial systems ultimately originates from photosynthesis, it remains debated to what extent increases in GPP are causing the inferred terrestrial carbon sink (Fatichi et al. 2019; Ruehr et al. 2023; Walker et al. 2021). While the response of photosynthesis to CO_2_ occurs biochemically at the leaf level, the land C sink is inferred via the global C budget at the planetary level (Friedlingstein et al. 2025; Keeling et al. 1996). Establishing a causal link between the rise in GPP and the sustained positive land C sink is challenging because a large range of processes and feedbacks affect the link, and numerous fluxes and pools are involved in the propagation of GPP changes to changes in terrestrial C storage (Fatichi et al. 2014; Walker et al. 2021).

Each Dynamic Global Vegetation Model (DGVM) proposes its own way of representing this causal link. They resolve a variety of ecosystem processes from leaf to ecosystem scales and simulate their responses and feedbacks to rising CO_2_ and climate change (Prentice et al. 2007). The representation of individual processes differs among DGVMs (Davies‐Barnard et al. 2020; De Kauwe et al. 2013; Rogers et al. 2017; Zaehle et al. 2014), leading to divergent simulations of C cycle changes during the past decades (Stocker et al. 2024). However, common to all DGVMs is that they conceive the land C cycle dynamics as a *cascade* of C, representing that all C that cycles in terrestrial ecosystems is fuelled by photosynthesis and that a change in photosynthesis has cascading effects on all ‘downstream’ C pools and fluxes (Koven et al. 2015; Weng et al. 2019). Following the notion of the C cascade, an increase in GPP is propagated to changes in net primary production (NPP), vegetation biomass, and soil carbon (Prentice et al. 2007; Walker et al. 2021; Weng et al. 2019). If the land C cycle behaved as a linear system, any relative change in GPP would propagate to the same relative change in all downstream fluxes and pools when a new steady‐state is (hypothetically) attained and in the absence of changes in external forcings (e.g., climate change) (Box 1). However, the linearity of this propagation is contentious, and it is an open question how effectively an increase in GPP, driven by elevated CO_2_ (eCO_2_), scales downstream fluxes and pools, and whether dynamics simulated by DGVMs generally agree with observed changes in ecosystem C pools and fluxes under eCO_2_.

The central role of photosynthesis as the dominant driver of ecosystem C balance changes has been questioned, and it has been argued that a ‘source‐driven’ conception of C cycle dynamics conflicts with key observations at different scales (Fatichi et al. 2014, 2019; Körner 2015; Kramer 1981). For example, a decrease in Rubisco and hence the maximum rate of carboxylation capacity (*V*_cmax_) under eCO_2_ could reduce leaf dark respiration and contribute to an increase in carbon use efficiency (CUE = NPP/GPP) (Jiang et al. 2020; Leakey et al. 2009; Smith 2017; Stocker et al. 2024). An increase in CUE would imply a non‐linear response along the C cascade with super‐proportional scaling. The link between NPP and biomass C storage could be affected by the tree growth‐longevity trade‐off (Bialic‐Murphy et al. 2024; Brienen et al. 2020; Bugmann and Bigler 2011; Büntgen et al. 2019; Körner 2017; Locosselli et al. 2020), whereby accelerated tree growth and increased NPP leads to an accelerated tree lifecycle (decreased vegetation turnover time) through amplified light competition, exclusion of short‐statured plants from the canopy, and increased tree mortality in closed forest stands (Marqués et al. 2023). Such relationships have been identified from various tree and forest stand‐level observations across large environmental gradients (tree rings, forest inventories), but it remains unclear whether similar relationships hold for the response to eCO_2_ (Marqués et al. 2023). If it does, the tree growth‐longevity trade‐off would imply decreased τ and sub‐proportional scaling. Furthermore, eCO_2_ could lead to a general shortage of soil nutrients, required for fixing C into biomass (Hungate et al. 2003; Norby et al. 2010; Vicca et al. 2012). Nutrient limitation could thus drive enhanced root respiration and/or labile C export to root symbionts (Jiang et al. 2020). A shift of plant C allocation towards more short‐lived fine root biomass and C exuded into the rhizosphere at the expense of allocation to long‐lived aboveground woody biomass is often (but not always (Walker et al. 2019)) recorded in ecosystem CO_2_ experiments (Ainsworth and Long 2005; De Kauwe et al. 2014; Jiang et al. 2020; Leakey et al. 2009; Rogers et al. 1995; Schneider et al. 2004; Song et al. 2019; Stocker et al. 2024; Terrer et al. 2021). Meanwhile, the allocation shift towards more short‐lived biomass under rising CO_2_ implies that the total ecosystem‐level biomass C stock increase is smaller compared to a case where the allocation remains unchanged.

In recent years, DGVMs have been further developed to resolve several of the mechanisms mentioned above, which may lead to non‐linear dynamics and may affect emergent behaviour and CO_2_ sensitivities of individual variables. For example, among the 16 models used for the TRENDY v11 model ensemble simulations and for the 2023 annual update of the Global Carbon Budget, presented in Friedlingstein et al. (2023), 10 models accounted for the effects of soil nitrogen on carbon cycling–in contrast to the first generation of DGVMs, where these fundamental resource limitations were not considered. Some of the N and P‐resolving models simulate less NPP and a shift towards more belowground allocation, driven by effects of nutrient limitation (Smith et al. 2014; Xia et al. 2013). Three models resolve size‐structured forest stand dynamics and vegetation demography as opposed to treating exclusively average‐sized individuals as done in earlier DGVM generations. These models thus resolve some of the processes that may lead to the growth rate‐longevity trade‐off (Fisher et al. 2018; Needham et al. 2020). These additional process representations are expected to influence the GPP propagation that fuels additional ecosystem C storage to some extent. Theoretically, the simulated model dynamics should reflect such model structural choices and process representations. However, the links between model structure and the effectively simulated dynamics of the C cascade are not straightforward to anticipate from the model descriptions due to feedbacks, non‐linearity, threshold‐type behaviour, and the ensuing state‐dependency of effective functional relationships. Hence, it is unclear to what extent current‐generation DGVMs (still) simulate predominantly source‐driven C cycle dynamics and what links along the C cascade–from GPP to NPP to vegetation carbon in different plant compartments–are responsible for a departure from linear systems dynamics. Furthermore, it is unclear how simulated linearity (or deviations from linearity) along the C cascade compare to observations of respective relationships from CO_2_ manipulation experiments.

Here, we introduce an analytical framework for measuring the degree of linearity in the response of vegetation pools and fluxes to rising CO_2_ for both DGVM simulation outputs and field experiments (Box 1). We focus on the links between GPP and NPP, between NPP and vegetation C, and between C in different plant compartments and total vegetation C. The further propagation to soil C storage has been investigated by a previous study (Terrer et al. 2021) and thus is not analysed here. We quantitatively measure whether individual DGVMs behave in a linear manner (*L* = 1, see Box 1) for a set of current‐generation DGVMs (TRENDY v11, S1). Considering that C‐N coupled models and vegetation‐demography models may behave differently from first‐generation C‐only and average‐individual models, we also classify models into distinct groups and summarise their respective behaviours. While some field studies were discussed above, it is often challenging to combine insights from diverse experimental setups and scales of observations and to evaluate DGVMs against such data (Caldararu et al. 2023). Addressing this challenge, Walker et al. (2021) integrated results from a large range of published sources and reported CO_2_ sensitivities for a range of variables. As a demonstration of method (Box 1), we investigate the patterns of the linearity metric *L* derived from this set of empirical data (Walker et al. 2021). This enables a model‐data comparison at a relatively high level of abstraction and aggregation, focusing on emergent behaviour and sensitivities. The framework applied here thus provides an approach for revealing the fundamental dynamics of DGVMs' representation of the land C cycle and facilitates the comparison of model simulations and confronting them with findings from experiments and field observations.

BOX 1 **The C Cascade in Terrestrial Ecosystems—A Linear System?**

C enters ecosystems through photosynthesis, measured at the ecosystem‐level by GPP. Once fixed from CO_2_ into organic compounds, C travels through a cascade of fluxes and pools. In a linear system, a constant fraction of C in a pool is transferred downstream, along the C cascade, per unit time, and the relative partitioning into multiple downstream pools remains constant. Such a representation of C dynamics in terrestrial ecosystems can be described by a linear system of equations (Luo, Wu, et al. 2001; Luo et al. 2022; Sierra et al. 2021; Weng et al. 2019). A linear representation of the C cascade implies that, given constant input fluxes, the hypothetical steady‐state pool sizes are determined by their input flux; the time until the steady‐state is approached scales with the turnover time of the respective pool; and the magnitudes of all fluxes scale linearly with the ultimate C source in the system – GPP.

To diagnose linearity (or deviations from linear behaviour) of DGVMs, we focus on three links in the C cascade. First, carbon use efficiency (CUE) represents the link between GPP and NPP (NPP = CUE × GPP). If CUE were constant, a relative change in GPP, here referred to as *R*_GPP_, induces an equal relative change in NPP (*R*_NPP_):

(1) $$R_{\mathrm{NPP}}=R_{\mathrm{GPP}}=\text{ΔNPP}/\mathrm{NPP}=\text{ΔGPP}/\mathrm{GPP}$$

Here, Δ denotes the absolute change, mostly due to eCO_2_ in this study. Second, a linear relationship between NPP and *C*_veg_* follows from a first‐order decay representation of the *C* dynamics, in which the loss of *C*_veg_ is proportional to the current value of *C*_veg_ and to the inverse of the turnover time (τ), while the gain of *C*_veg_ is given by NPP. For a model following linear dynamics (constant effective τ), the temporal change in *C*_veg_ (d*C*_veg_/dt) is given by the gain minus the loss, hence:

(2) $$dC_{\mathrm{veg}}/\mathrm{dt}=\mathrm{NPP}–C_{\mathrm{veg}}/\tau$$

The steady‐state vegetation C pool size (*C*_veg_*) is attained when d*C*_veg_/dt = 0 and is thus given by the product of NPP and the (effective) τ of C in that pool.

(3) $${C_{\mathrm{veg}}}^{*}=\mathrm{NPP}\times \tau$$

Hence, with τ being constant, a relative change in NPP induces an equal relative change in *C*_veg_*:

(4) $$R_{{\text{Cveg}}^{*}}=R_{\mathrm{NPP}}=\Delta {C_{\mathrm{veg}}}^{*}/C_{\mathrm{veg}}=\text{ΔNPP}/\mathrm{NPP}$$

Note that Equation (4) describes relationships between NPP and *total* vegetation C, representing the sum of *C* stored in individual plant compartments (leaves, wood, fine roots), and that τ represents an *effective* turnover rate, influenced by allocation to the different compartments. Since the typical turnover time of *C* varies strongly between plant compartments, the allocation (the relative partitioning) of NPP for building new biomass in different compartments is influential for the *C* dynamics and is implicitly assumed constant in Equation (4).

Third, under constant allocation (and constant τ), the steady‐state relative changes in root *C* and total vegetation C are identical.

(5) $$R_{{\text{Croot}}^{*}}=R_{{\text{Cveg}}^{*}}=\Delta C_{{\text{root}}^{*}}/C_{\text{root}}=\Delta {C_{\mathrm{veg}}}^{*}/C_{\mathrm{veg}}$$

An analogous relationship applies for the other vegetation C compartments. That is, *C*_root_ may be replaced by *C*_leaf_ or *C*_wood_ in Equation (5).

Linearity of these links can be measured by the ratio of relative changes and illustrated by regressing the relative change of the two variables of each link against each other. Departure of the ratio from 1 or points plotting off the 1:1 line in the regression indicates non‐linear model behaviour. We thus consider the following three linearity metrics:

(6) $$L_{\mathrm{NPP}:\mathrm{GPP}}=R_{\mathrm{NPP}}/R_{\mathrm{GPP}}$$

(7) $$L_{\text{Cveg}*:\mathrm{NPP}}=R_{{\text{Cveg}}^{*}}/R_{\mathrm{NPP}}$$

(8) $$L_{\text{Croot}*:\text{Cveg}*}=R_{{\text{Croot}}^{*}}/R_{{\text{Cveg}}^{*}}$$

Environmental change may affect CUE, τ, and allocation, e.g., through heat and drought‐driven tree mortality, the effect of temperature on respiration rates, or the influence of reactive N deposition on growth and allocation. In contrast, CO_2_ exerts a direct control only on photosynthesis and GPP, while CUE, τ, and allocation have no *explicit* dependence on CO_2_. Note that these processes may change *implicitly* in response to eCO_2_ due to feedbacks and state‐dependencies of functional relationships, leading to non‐linear behaviour, even if process representations implemented in models have no explicit CO_2_ dependence. For example, allocation fractions are may not be influenced formulated as a function of CO_2_, but as a function of nutrient and water availability and current biomass pool sizes, which are in turn affected by the CO_2_ response of the ecosystem. Thus, *R* and *L* terms can be diagnosed from model simulations where only CO_2_ is changing, while climate is held constant, to quantify the linearity of the terrestrial C cycle dynamics in response to changing CO_2_. As such, *L* = 1 indicates a perfectly linear behaviour. *L*_NPP:GPP_ = 1 indicates that CUE is not affected by eCO_2_. *L*_Cveg*:NPP_ = 1 implies constant vegetation turnover time and allocation patterns. *L*_Croot*:Cveg*_ = 1 indicates constant root: shoot ratios and above vs. belowground allocation patterns. Non‐linearity could stem from multiple processes that violate Equations ((1), (2), (3), (4), (5))–((1), (2), (3), (4), (5)), including, (a) dynamic NPP allocation fraction, (b) dynamic turnover time, or (c) various feedback loopsover, for example, higher NPP leading to more leaf area, enhanced total photosynthesis, and further increases in NPP, and other non‐linear model structures. While the analytical framework presented here enables a quantification of emergent model behaviour in terms of linearity along the C cascade, it does not separate relative contributions of driving mechanisms.

Furthermore, the *L* metric can be related to CO_2_ sensitivities, reported in the literature (Walker et al. 2021). The CO_2_ sensitivity of a given variable *x* (*β*_*x*_) is commonly quantified as the logarithmic response ratio of *x*, evaluated from observed changes under (mostly experimentally manipulated) varying CO_2_, normalised by the logarithmic change ratio of CO_2_ (*c*_*a*_):

(9) $$\beta_{x}=\frac{\ln\left(x/x_{0}\right)}{\ln\left(c_{a}/c_{a,0}\right)}\approx \frac{\Delta x/x}{\Delta c_{a}/c_{a}}$$

The second, approximative, identity in Equation (9) holds for small changes in *x* (Δ*x*). Note that the normalisation by the relative CO_2_ change is necessary for observational data because observations of two linked variables *x* and *y* may be obtained from separate sources, e.g., experiments where CO_2_ was manipulated in different ways. In contrast, metrics *R* of two linked variables (*R*_*x*_ and *R*_*y*_) are not normalised by the CO_2_ change in Equations (1), (4), and (5) as they are evaluated from the same model simulation. Hence, assuming that *β* sensitivity terms are constants and insensitive to the level of the reference CO_2_ concentration and using the approximative equation above, we get:

(10) $$L_{y:x}=\beta_{y}/\beta_{x}.$$

Equation (10) enables a general data‐model comparison, using results from model evaluations yielding *L*_*y:x*_ and from *β* sensitivity terms reported in the literature (Walker et al. 2021).

## 2 Methods

### 2.1 Model Output Processing

We obtained outputs of TRENDY v11 S1 simulations from 16 models (Friedlingstein et al. 2023; Sitch et al. 2024). In these simulations, climate and land use were held constant at pre‐industrial levels, while atmospheric CO_2_ concentrations changed as observed—from 285 ppm to 407 ppm—based on atmospheric and ice core measurements (Sitch et al. 2024). In S1, nitrogen fertilisation of agricultural land was fixed at pre‐industrial levels, while varying nitrogen deposition on all land portions (agricultural and non‐agricultural land) was prescribed from observations. Fire‐enabled models allowed varying fire ignition probability. Using S1 simulations precludes climate‐driven effects on ecosystem C dynamics. TRENDY provides no simulations with varying CO_2_ and fixed N deposition. Therefore, this study includes both the effect of eCO_2_ and N deposition. Simulations were started from a dynamic steady‐state in year 1700 and transiently covered the period up to the year 2022. We identified models as ‘C‐N coupled models’ (*n* = 10) by referring to the Supporting Information of Friedlingstein et al. (2022). We identified models as ‘vegetation demography models’ (*n* = 3) by consulting modellers. We considered vegetation demography models as those that simulate tree‐size dependent demographic rates (growth, mortality, fecundity) and distinguish trees (or cohorts of trees) of different sizes within each plant functional type (PFT) and within a model gridcell.

**FIGURE 1:**
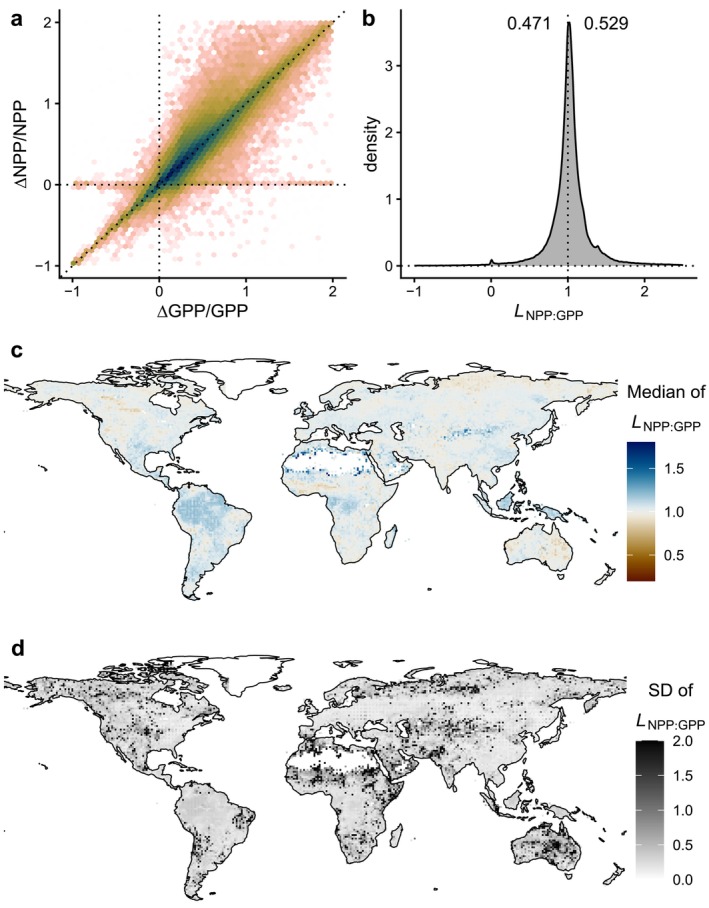
The relationship between the relative change of net primary productivity (*R*_NPP_ = ΔNPP/NPP) and the relative change of gross primary productivity (*R*_GPP_ = ΔGPP/GPP) under rising CO_2_ and changing N deposition, aggregated across models. (a) Density scatter plot of *R*_NPP_ vs. *R*_*G*PP_ values across all gridcells, pooled from balanced samples of all models. Dark colours denote high density, while bright colours denote low density of data points. The dotted lines are the *y* = 0 line and the *y* = *x* line. (b) Density of the distribution of *L*_NPP:GPP_ (=*R*_NPP_/*R*_GPP_) values, pooled from balanced samples of all models. Annotated numbers indicate the fraction of points below (left) and above (right) *L* = 1. (c) Spatial pattern of *L*_NPP:GPP_, shown as the median per gridcell across models. (d) Spatial pattern of *L*_NPP:GPP_, shown as standard deviation (SD) per gridcell across models. Large standard deviation suggests large inter‐model spread.

Although land use and climate do not change in TRENDY S1 simulations, the land cover fraction of different PFTs may change in response to rising CO_2_ (and N deposition), or in response to changing wildfire (driven by changing CO_2_ and N deposition) in models that simulate dynamic PFT distributions. In particular, shifts between grasses and trees are accompanied by substantial changes in aboveground biomass stocks even if NPP remains at similar levels. This is due to changes in the fraction of C allocated to (long‐lived) wood that is zero for grasses. For models with dynamic PFT (LPJ‐GUESS, JULES, and LPX‐Bern), we thus excluded gridcells for which the land cover fraction of the dominant PFT changed over 10% between simulation start and end. Sparsely vegetated regions (e.g., arid deserts and tundra) may have extreme or no relative changes in carbon flux. We noticed that most of these low‐biomass gridcells lead to *L*_Cveg*:NPP_ = 0 (Figure S24). Therefore, we excluded grid cells with *C*_veg_ below the 5% quantile.

For *L*_Croot:Cveg_, LPJ‐GUESS, DLEM, JSBACH and LPJ were excluded from the analysis because *C*_root_ of these models was not reported. For the same reason, some models were excluded from analysing *L*_Cleaf:Cveg*_ and *L*_Cwood:Cveg_. JULES and IBIS include coarse root biomass in *C*_wood_, while other models include it in *C*_root_ (Figure S23). Hence, figures associated with *C*_root_ should be interpreted with this in mind.

### 2.2 Quantifying Linearity

For a given model, the relative change *R* of variable *X* was calculated for each gridcell *i* as follows:

(11) $$R_{X}\left(i\right)=\frac{X\left(i,t_{1}\right)-X\left(i,t_{0}\right)\;}{X\left(i,t_{0}\right)}$$

where *X*(*i*, *t*_0_) is the mean of *X* over the first 10 simulation years, 1700 to 1709 (*t*_0_) and *X*(*i*, *t*_1_) is the mean over years 2013 to 2022 (*t*_1_). As variables *X*, we considered GPP, NPP, total biomass (*C*_veg_), root biomass (*C*_root_), and other compartments of biomass. The linearity term *L* of the link between two variables *X* and *Y* was calculated as the ratio of their relative changes.

(12) $$L_{Y:X}\left(i\right)=\frac{R_{Y}\;\left(i\right)\;}{R_{X}\;\left(i\right)}$$

We quantified *L* for the link between GPP and NPP (*L*_NPP:GPP_), between NPP and *C*_veg_ (*L*_Cveg*:NPP_), and between *C*_veg_ and *C* in individual plant biomass compartments (leaves, woody biomass, and roots).

Linearity (*L*) of each DGVM is ideally calculated by quantifying the ratio of relative changes in connected pools and fluxes at their respective steady‐state (Box 1). However, the model outputs analysed here are from transient simulations, forced by gradual CO_2_ changes that increased from a stable preindustrial level (285 ppm in 1700) in an accelerating manner to its current level (407 ppm in 2022). At this time scale, pools with turnover time scales on the order of decades and longer cannot be assumed to track their steady‐state size during the simulations. For example, in forests, most of the vegetation carbon (*C*_veg_) is contained in woody biomass, which has a turnover time of decades to centuries (Locosselli et al. 2020). Thus, when dealing with links between fluxes and pools (e.g., *L*_Cveg*:NPP_), the steady‐state of such pools needs to be estimated from the reported transient state.

By re‐arranging Equation (2) (Box 1), the turnover rate (τ) can be estimated from NPP, the current (transiently changing) vegetation C pool size (*C*_veg_(t)) and its temporal change (d*C*_veg_/dt):

(13) $$\tau =C_{\mathrm{veg}}\left(t\right)/\left(\mathrm{NPP}\left(t\right)–dC_{\mathrm{veg}}/\mathrm{dt}\right)$$

By combining Equations (3) and (14), we get an expression for estimating the steady‐state vegetation C pool size from the transient dynamics:

(14) $${C_{\mathrm{veg}}}^{*}=C_{\mathrm{veg}}\left(t\right)\times \mathrm{NPP}\left(t\right)/\left(\mathrm{NPP}\left(t\right)–{\mathrm{dC}}_{\mathrm{veg}}/\mathrm{dt}\right)$$

Equation (14) was applied here for estimating the steady‐state vegetation C pool size *C*_veg_* from the transient simulation outputs, whereby *C*_veg_(t) in Equation (14) was taken as the mean of *C*_veg_ across years 2013 to 2022. d*C*_veg_/dt in Equation (14) was taken as $\left(C_{\mathrm{veg}}\left(2022\right)-C_{\mathrm{veg}}\left(2013\right)\right)/10$. NPP(t) was calculated as the mean across 2013 to 2022. To test whether the *L* outcomes are sensitive to the choice of the time period, we performed analyses also for years 2008–2017 instead of 2013–2022, while keeping everything else unchanged (Figure S25).

Please note that Equations (3) and (14) assume first‐order linear kinetics. Hence, estimating *C*_veg_* to assess linearity bears a conceptual circularity. Therefore, we also performed an evaluation of the reliability of this steady state‐estimation method (Equation 14) for two alternative simple demonstration models of the C cascade and a state‐of‐the‐art vegetation demography model (BiomeEP) (Marqués et al. 2023) that either (by design) follow linear dynamics or not (Figures S31–S42). Models were forced with transiently changing CO_2_, then followed by a phase with constant CO_2_ (and other forcings) for 1000 simulation years to attain a steady state. This evaluation demonstrates that the steady‐state correction yields a more accurate estimation of the actual steady state and hence a more accurate quantification of the linearity term *L* for all three demonstration models (more details in the Discussion).

GPP is controlled by the physiology of photosynthesis, which has a relatively rapid response to CO_2_, and by the structure of the canopy. Leaf area changes in response to the environment evolve over seasonal time scales and respond to altered CO_2_ within years or faster, as seen for example at the Duke FACE experiment (McCarthy et al. 2007). We thus did not apply the steady‐state correction (Equation 14) on GPP and used unmodified model outputs for respective analyses. NPP is equal to GPP minus autotrophic respiration (*R*_a_). *R*_a_ contains growth respiration (which scales with NPP), fine root, leaf, and sapwood maintenance respiration. Fine root and leaf productivity and biomass in models respond on fast time scales (seasons to years), which should thus equilibrate correspondingly fast. Sapwood respiration may take longer to equilibrate if taken to be proportional to sapwood mass, if sapwood mass scales with total woody biomass, and given that woody biomass has a decadal to centennial response time scale. However, sapwood respiration is only a relatively small fraction of *R*_a_ (Zhang‐Zheng et al. 2024). Based on the above, we neglected non‐equilibrium effects for GPP and NPP. Similarly, for *C*_leaf_ (Figures S13–S15), we assumed equilibrium given the short lifetime of leaf biomass (months to years) (Gill and Jackson 2000; Wright et al. 2004). Thus, *L*_Cleaf:Cveg*_ is presented.

For the link between *C*_root_ and *C*_veg_, and for the link between *C*_wood_ and *C*_veg_, the steady‐state correction would be ideal, because these carbon pools have long turnover times, ranging from several decades to centuries (Xue et al. 2017; Yu et al. 2023). However, we could not apply the steady‐state correction (Equation 14) because DGVMs did not report root NPP and woody NPP. We chose to compare *C*_wood_ to uncorrected *C*_veg_ because they both have decadal turnover times and because *C*_veg_ is dominated by *C*_wood_. The steady‐state correction may thus cancel out. Therefore, we analysed *L*_Cwood:Cveg_. For *C*_root_, we also provide results where we applied the steady‐state correction on *C*_veg_ for an alternative evaluation of *L*_Croot:Cveg*_ (Figures S10–S12). We found very similar patterns to the uncorrected version (*L*_Croot:Cveg_) (Figures S7–S9), but with a general tendency for lower values. The lack of steady‐state correction for *C*_wood_ is owed to the absence of plant compartment‐specific NPP model outputs and constitutes limitations of this study.

**FIGURE 2:**
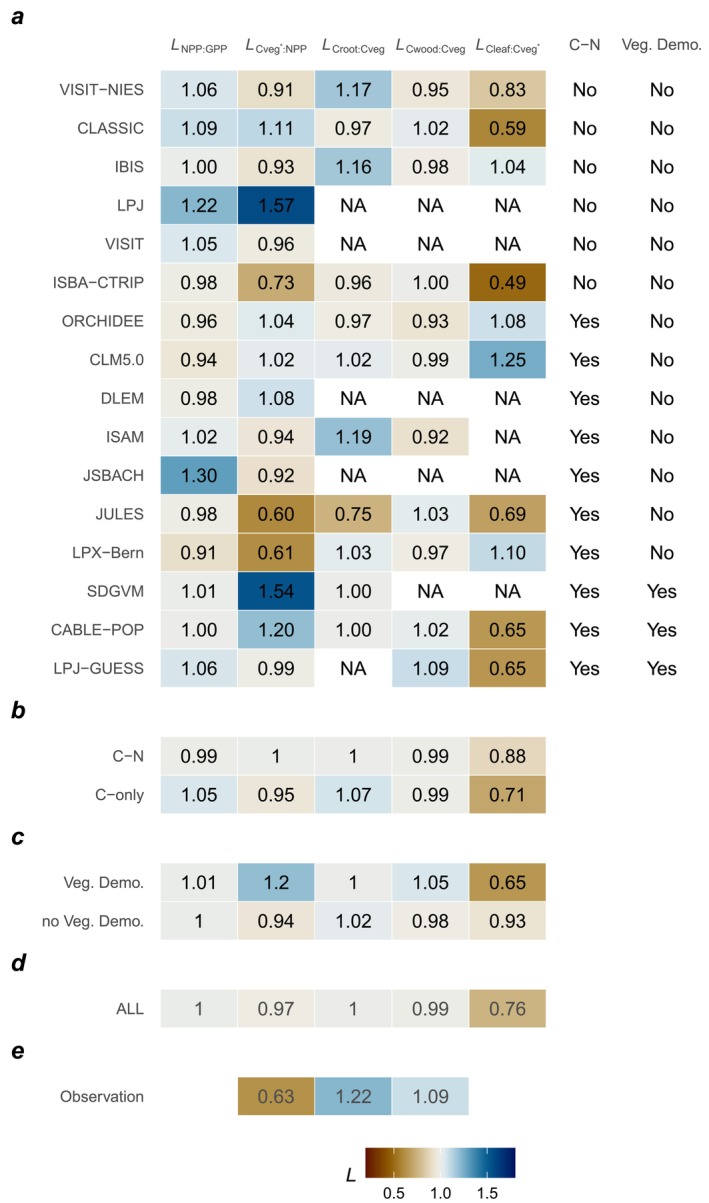
Overview of linearity terms *L*_NPP:GPP_, *L*_Cveg*:NPP_, *L*_Croot:Cveg_, *L*_Cwood:Cveg_ and *L*_Cleaf:Cveg*_ across models. We show the median of all gridcells for each model (a). We also show linearity terms *L* as a median across all models (d) and model groups according to whether the model considered carbon nitrogen coupling (C‐N) (b) and vegetation demography (Veg. Demo.) (c). Values in ‘Observation’ represent median values of *L*, shown in Figure 4b,d,f. Values of the linearity terms are given as annotations and accordingly coded by colours. The interquartile range (IQR) ancillary to the median is provided in Supporting Information. White cells (NA) in (a) indicate missing values due to the non‐availability of compartment‐specific vegetation C outputs. See Supporting Information for models' C‐N and Veg. Demo. grouping information.

### 2.3 Illustration of Linearity

The relative increase terms *R* and linearity terms *L* are evaluated for each link, gridcell, and model. We provide results at different levels of aggregation, including spatial maps, density scatter plots, and histograms of the distributions of *L* for each link and model and a summary table of median values for *L*. For the spatial maps, we calculated the median of *L* terms from all models for each gridcell where the median represents overall *L* across models. As DGVMs vary in resolution, we first resampled model outputs to a coarser common resolution (1° in longitude and latitude). For the density scatter plot and histogram of the term *L*, such resampling was not suitable since resampling would reduce the range of values (extreme values would be smoothed out). The outputs of the model with the coarsest spatial resolution (VISIT) were provided for a total of 58,697 land gridcells. Therefore, we randomly sampled 58,697 gridcells from each model and pooled all values from all models to present the ‘overall’ pattern (e.g., Figure 1a,b) and the ‘all’ panel in Supporting Information. The random sampling thus ensures that outputs from low spatial resolution models are not underrepresented in the analysis of pooled data from multiple models.

**FIGURE 3:**
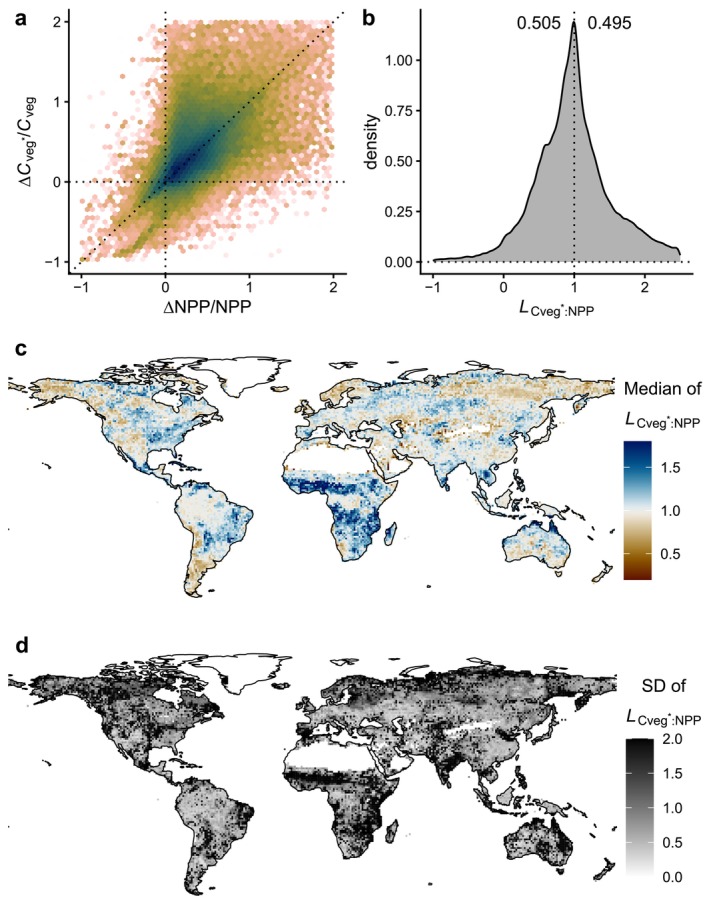
The relationship between the relative change of steady‐state vegetation C (*R*_Cveg*_ = ΔC_veg*_/C_veg_) and the relative change of net primary productivity (*R*_NPP_ = ΔNPP/NPP) under rising CO_2_ and changing N deposition, aggregated across models. (a) Density scatter plot of *R*_Cveg*_ vs. *R*_NPP_ values across all gridcells, pooled from balanced samples of all models. Dark colours denote high density, while bright colours denote low density. The dotted lines are the *y* = 0 line and the *y* = *x* line. (b) Density of the distribution of *L*_Cveg*:NPP_ (=*R*_Cveg*_/*R*_NPP_) values, pooled from balanced samples of all models. Annotated numbers indicate the fraction of points below (left) and above (right) *L* = 1. (c) Spatial pattern of *L*_Cveg*:NPP_, shown as the median per gridcell across models. (d) Spatial pattern of *L*_Cveg*:NPP_, shown as standard deviation per gridcell across models. Large standard deviation suggests large inter‐model spread.

### 2.4 Synthesis of Field Evidence

The analytical framework outlined in Box 1 (Equations 9 and 10) can be used to translate *β* sensitivity terms reported by Walker et al. (2021) to corresponding linearity terms (*L*). This enabled us to derive *L* from field observations. *β* values derived by Walker et al. (2021) were sourced from diverse types of observations and across multiple scales. However, the analytical framework controls for effects of the magnitude of CO_2_ change (Equation 9) and enables a rough comparison that yields quantitative insights into the ratio of CO_2_ responses of two linked variables (Equation 10). At this level of detail and precision, we consider the comparison of these observation‐derived CO_2_ sensitivities with values obtained from model outputs to be meaningful and informative. Walker et al. (2021) provide *β* estimates and their uncertainties for 𝛽_Cveg_, 𝛽_Croot_, 𝛽_Cwood_ and biomass production (𝛽_BP_ ≈ 𝛽_NPP_) originating from different sources and data types. Walker et al. (2021) did not provide GPP (but net assimilation rate only) so 𝛽_GPP_ */*𝛽_NPP_ is missing. We derived respective normal distributions using the 95% confidence interval provided by Walker et al. (2021). We then generated 100,000 randomly sampled combinations of 𝛽_x_ and 𝛽_y_, where each source of the *x* and *y* data had an equal probability of being selected. Each combination yielded one value of *β*_*x*_ */β*_*y*_ and thus one *L*. The random sample generates long tails in the distribution of *β*, extending outside a realistic range. Therefore, we capped values between −1 and 2.5 for visualisations. Following Equation (10), the derived *β*_*x*_ */β*_*y*_ could be compared to model *L*, since these values are normalised by the change in CO_2_. We use only sensitivity estimates from experimentally controlled observations (‘eCO_2_’ in Walker et al. 2021). We used the following variables from tab. 2 of Walker et al. (2021): ‘BP’ interpreted here as NPP; ‘*C*_veg’ interpreted here as *C*_veg_; ‘*C*_wood’ and ‘*C*_wood_ag’ interpreted here as *C*_wood_; and ‘*C*_root’, ‘*C*_veg_bg, ‘*C*_veg_root’, ‘*C*_veg_bg_inc’, ‘*C*_fine‐root’ and ‘BP_fine‐root’ interpreted here as *C*_root_. For the latter, we included also variables measuring biomass *productivity*, not just *pool size*. This is to overcome data sparsity and rests on the assumption of a constant root turnover. Walker et al. (2021) did not analyse experimental data for the link between GPP and NPP as GPP is not commonly estimated in CO_2_ experiments and no values for GPP under ‘eCO_2_’ were reported in Walker et al. (2021). In addition, we summarised empirical evidence and our interpretation for implication for different *L* terms in Table S1.

**FIGURE 4:**
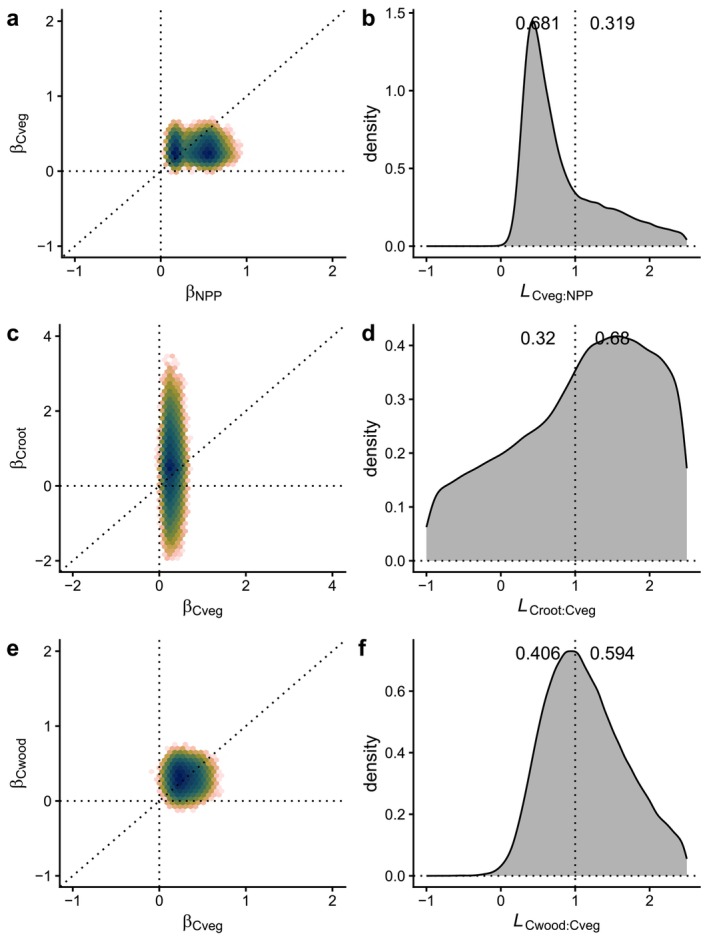
Compilation of experimental evidence regarding linearity along the C cascade. Shown are (a, b) relationships between sensitivities NPP and total vegetation C; (c, d) total vegetation C and root C; and (e, f) total vegetation C and wood C. Left panels (a, c, e) show the density of combinations of CO_2_ sensitivity terms (𝛽), sampled within their normal error distribution given by Walker et al. (2021), with dark colours indicating high density of points. The dotted 1:1 line indicates equal CO_2_ sensitivity between each of the two connected variables, indicating a linear (proportional) link of effects. Right panels (b, d, f) show the distribution of linearity terms *L*, defined as the ratio of the two corresponding CO_2_ sensitivity terms (𝛽, see Equation 10), bounded between [−1, 2.5]. The dotted vertical line at *L* = 1 indicates linear behaviour. Annotated numbers indicate the fraction of points below (left) and above (right) *L* = 1. The random sample generates long tails in the distribution of β, so we capped values.

## 3 Results

### 3.1 The Link Between GPP and NPP

The magnitudes of relative changes in GPP (*R*_GPP_) varied across models. Some models simulated relatively small increases (SDGVM: 13% [interquartile range across gridcells: 7%]; CLASSIC: 11% [IQR 23%]), while others simulated larger increases (e.g., ISBA‐CTRIP: 39% [IQR 20%], ORCHIDEE: 31% [IQR 40%], ISAM: 31% [15%], and CABLE‐POP: 31% [IQR 26%]) (Supporting Information). However, while *R*_GPP_ and *R*_NPP_ varied substantially across models, their ratio (*L*_NPP:GPP_) was tightly conserved. Pooled outputs showed narrow clustering of *R*_GPP_ vs. *R*_NPP_ along the 1:1 line (Figure 1) and a tight distribution of *L*_NPP:GPP_ around unity (1.02 [IQR: 0.22]; Supporting Information).

Although the simulated link between GPP and NPP is predominantly linear when considering pooled outputs from all models (*L*_NPP:GPP_ = 1), individual models show strong deviations from *L*_NPP:GPP_ = 1 (Figures S1–S3). Several models (CLASSIC, LPJ, LPJ‐GUESS, SDGVM, VISIT, JSBACH) exhibit patterns of *L*_NPP:GPP_ > 1 for more than 50% of gridcells (Figure S1). For JSBACH, 76% of all gridcells exhibit *L*_NPP:GPP_ > 1. Several models show the opposite, including LPX‐Bern, ORCHIDEE, and DLEM. In LPX‐Bern, only 25% exhibit *L*_NPP:GPP_ > 1, and a substantial portion of gridcells exhibit zero NPP change despite changes in GPP (Figures S1 and S2).

Geographical patterns emerge on average across all models, with a tendency towards *L*_NPP:GPP_ > 1 in many moist tropical regions and *L*_NPP:GPP_ < 1 in drier regions high‐northern latitudes (Figure 1c). However, clear geographical patterns are also evident within models and vary strongly across models (Figure S3). For example, JSBACH exhibits a clear tendency of *L*_NPP:GPP_ > 1 across the globe, but not for tropical rainforests. ORCHIDEE simulates an opposite pattern with a tendency for *L*_NPP:GPP_ < 1 across the globe, but > 1 in tropical rainforests. In several models (IBIS, VISIT, VISIT‐NIES), *L*_NPP:GPP_ is rather uniform across the globe or doesn't exhibit a clear latitudinal pattern. For several models with a strong geographical variation (CABLE‐POP, SDGVM), when all grid cells are pooled together, *L* > 1 and *L* < 1 on the map (Figure S3) counter‐balance each other and regress to *L* = 1 (Figure S1).

Across most models without C‐N coupling, there was a tendency for *L*_NPP:GPP_ > 1 with median *L* = 1.06 (Figure 2). Five out of the 10 C‐N coupled models simulated *L*_NPP:GPP_ < 1, while the other five models simulated an opposite tendency, yielding median *L* = 0.99. We found no systematic differences between models incorporating vegetation demography (*L* = 1.01) versus average‐individual models (*L* = 1.00). Since individual models do not agree on *L*_NPP:GPP_ and the geographical patterns include both *L* > 1 and *L* < 1, when presented as multi‐model median (across the whole models ensemble), the simulated link between GPP and NPP is predominantly linear overall (*L*_NPP:GPP_ = 1.00 [IQR 0.22]). There is no direct empirical evidence for patterns in *L*_NPP:GPP_ as GPP is not commonly estimated in CO_2_ experiments.

### 3.2 The Link Between NPP and Vegetation Carbon

The overall median of *L*_Cveg*:NPP_ is around unity at 0.97 [IQR 0.73] (Figure 3). Unity indicates that, on average, the change in vegetation carbon tends to be linearly related to changes in NPP. However, the distribution of *L*_Cveg*:NPP_ was much wider than that of *L*_NPP:GPP_, owing to more spatial variation and more inter‐model spread. Although most individual models clearly deviate from an overall linear relationship between NPP and *C*_veg_, the general pattern of the results pooled from all models indicates a peak of the distribution of *L*_Cveg*:NPP_ at unity and a roughly balanced share of pixels across models indicating *L*_Cveg*:NPP_ > 1 vs. *L*_Cveg*:NPP_ < 1 (Figure 3).

**FIGURE 5:**
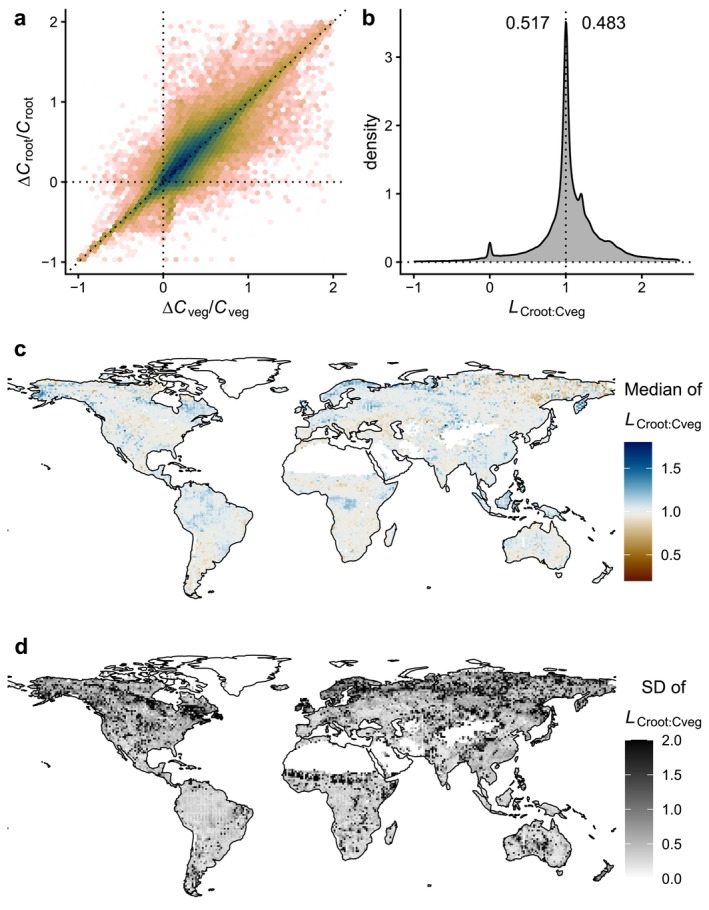
The relationship between the relative change of root biomass (*R*_Croot_ = ΔC_root_/C_root_) and the relative change of total vegetation biomass (*R*_Cveg_ = ΔC_veg_/C_veg_) under rising CO_2_ and changing N deposition, aggregated across models. (a) Density scatter plot of *R*_Croot_ vs. *R*_Cveg_ values across all gridcells, pooled from balanced samples of all models. Dark colours denote high density, while bright colours denote low density. The dotted lines are the *y* = 0 line and the *y* = *x* line. (b) Density of the distribution of *L*_Croot:Cveg_ (=*R*_Croot_/*R*_Cveg_) values, pooled from balanced samples of all models. Annotated numbers indicate the fraction of points below (left) and above (right) *L* = 1. (c) Spatial pattern of *L*_Croot:Cveg_, shown as the median per gridcell across models. (d) Spatial pattern of *L*_Croot:Cveg_, shown as standard deviation per gridcell across models. Large standard deviation suggests large inter‐model spread.

The patterns in *R*_Cveg*_ versus *R*_NPP_ were very diverse across models (Figures S4–S6). For example, ISBA‐CTRIP exhibited overall no clear relationship between the magnitudes of relative changes in NPP and *C*_veg_ and a clear dominance of *L*_Cveg*:NPP_ < 1. In ORCHIDEE, there was a tendency of *L*_Cveg*:NPP_ > 1 for small values of *R*_NPP_ and *L*_Cveg*:NPP_ < 1 for large values of *R*_NPP_. Several models had *L*_Cveg*:NPP_ > 1 for a clear majority (> 60%) of gridcells (CABLE‐POP, DLEM, LPJ, SDGVM). Among them, DLEM, LPJ, and CLM5.0 had a substantial portion of gridcells for which *R*_Cveg*_ was much larger than *R*_NPP_ (Figure S5). In contrast, several models have *L*_Cveg*:NPP_ < 1 for a clear majority of gridcells (IBIS, ISBA‐CTRIP, JULES, LPX‐Bern, VISIT‐NIES). Several models exhibited multimodal distributions of *L*_Cveg*:NPP_ with one peak around zero, corresponding to no change in *C*_veg*_. No clear commonalities could be found among carbon‐nitrogen coupled vs. C‐only models (Figure 2b). On average, both groups exhibited a median of *L*_Cveg*:NPP_ very close to unity. Vegetation demography models overall have a median *L*_Cveg*:NPP_ > 1, inconsistent with theoretical expectations considering the tree growth‐longevity trade‐off (Marqués et al. 2023), while non‐demography models overall show *L*_Cveg*:NPP_ < 1.

Geographical patterns emerged on average across all models, with a tendency for *L*_Cveg*:NPP_ < 1 in arid and cold regions (e.g., Sahara, northeastern Siberia, interior Eurasia) and *L*_Cveg*:NPP_ > 1 in seasonally dry tropical forests and savannah regions (Figure 3c). However, spatial patterns of *L*_Cveg*:NPP_ were very diverse across individual models (Figure S6). Across forested regions, several models consistently simulate *L*_Cveg*:NPP_ > 1 (CABLE‐POP, CLASSIC, SDGVM), while other models consistently simulate *L*_Cveg*:NPP_ < 1 in forest regions (IBIS, ISAM, LPX‐Bern). For several models (DLEM, ISAM, LPJ‐GUESS, ORCHIDEE, VISIT, VISIT‐NIES), *L*_Cveg*:NPP_ was near unity for tropical rainforests. Observational data was relatively sparse (only two reported *β*_Cveg_ values and seven *β*_Croot_ values were used from Walker et al. (2021)). Yet, it suggested a clear dominance of a larger 11 sensitivity in NPP than in *C*_veg_, implying *L*_Cveg:NPP_ < 1 (Figure 4).

### 3.3 The Link Between Leaf, Wood, Root and Total Vegetation Carbon

Across models and gridcells, *L*_Croot:Cveg_ was distributed tightly around 1 (median 1.00, [IQR 0.32]), indicating overall a clear tendency of equal relative changes in root and total vegetation biomass and no general shift in allocation under rising CO_2_ (and changing N deposition) (Figure 2). This linear pattern was evident within some models and was most clearly expressed for CABLE‐POP and SDGVM (Figures S7 and S8). However, some models deviated from this pattern and simulated higher relative changes in root biomass than in total vegetation biomass (*L*_Croot:Cveg_ > 1) for a clear majority (> 60%) of gridcells (IBIS, ISAM, VISIT‐NIES). Some models simulated *L*_Croot:Cveg_ < 1 for a clear majority of gridcells (JULES, ORCHIDEE). The C‐only models simulated, on average, a slightly higher *L*_Croot:Cveg_ (median 1.07) than C‐N coupled models (median 1.0, Figure 5).

*L*_Cwood:Cveg_ generally exhibited an opposite pattern to *L*_Croot:Cveg_ (Figure 2), suggesting that more allocation to *C*_root_ is often associated with less allocation to woody biomass (*C*_wood_). This pattern was most clearly evident from IBIS, ISAM, and VISIT‐NIES, which showed *L*_Cwood:Cveg_ < 1 (Figure S16), opposite to *L*_Croot:Cveg_. Patterns of *L*_Cleaf:Cveg*_ appeared independent of allocation to wood and roots (Figure 2). Four models (especially CLM5.0 and LPX‐BERN) showed *L*_Cleaf:Cveg*_ > 1, while six models showed strong *L*_Cleaf:Cveg*_ < 1. Overall, the median of *L*_Cleaf:Cveg*_ was at 0.76 [IQR 0.81], suggesting less relative allocation to leaves than to total vegetation pools under eCO_2_.

We also analysed model outputs where we applied the steady‐state correction on *C*_veg_ for an alternative evaluation of *L*_Croot:Cveg*_ (Figure S10). We found similar patterns compared to the uncorrected version (*L*_Croot:Cveg_) (Figure S7), but with a general tendency for lower values for (corrected) *L*_Croot:Cveg*_ than for (uncorrected) *L*_Croot:Cveg_.

The geographical distribution of simulated *L*_Croot:Cveg_ varied across models (Figure S9). For example, ORCHIDEE simulated values < 1 in high northern latitudes; JULES simulated values < 1 in boreal and tropical forest regions. IBIS and LPX‐Bern simulated values > 1 in tropical forest regions, while ISAM and VISIT‐NIES simulated values > 1 in most geographical regions. Some models exhibit a rather homogenous spatial pattern across the globe with values near 1 (CABLE‐POP, CLM5.0, SDGVM), while others simulate strong spatial variations (LPX‐Bern, CLASSIC).

The compilation of field evidence suggests 𝛽_Croot_ > 𝛽_Cveg_ (Figure 4) for a clear majority of sampled data points (68% of data points). The peak of the distribution for *L*_Cwood:Cveg_ was slightly below 1 (Figure 4), in line with the pattern found in model outputs. However, many observations‐based data points suggested 𝛽_Cwood_ > 𝛽_Cveg_ and thus, uncertainty was overall high in observations‐based estimates of linearity, owing to substantial variation in field‐based 𝛽 of respective variables and the small data sample.

## 4 Discussion

We investigated the linearity of links along the modelled C cascade—from GPP to NPP, to vegetation biomass and its partitioning into different plant compartments. Our approach was motivated by the following two aspects. First, it decomposes the terrestrial carbon (C) cascade into a sequence of diagnostically evaluable links among fluxes and pools that together determine the land C‐cycle response to rising CO_2_. Assessing these successive relationships reduces the confounding influence of internal feedbacks by effectively normalising each response with respect to changes in the next upstream flux or pool. This provides a more tractable framework than evaluating the CO_2_ sensitivity of individual variables in isolation. For example, the sensitivity of vegetation carbon (*C*_veg_) to CO_2_ depends not only on gross primary productivity (GPP), but also on the coupling of GPP to net primary productivity (NPP) and of NPP to *C*_veg_, each governed by multiple underlying processes.

Second, the analytical framework for diagnosing linearity is compatible with commonly quantified CO_2_ sensitivity (*β*) terms (Box 1). Thus, comparison of diagnosed *L* against empirical patterns yields insight about model dynamics beyond commonly used static benchmarks and thereby tests model dynamics that are particularly relevant in the context of C cycle changes in response to the continued rise in atmospheric CO_2_.

*L* near unity has a clear interpretation: it indicates equal relative changes in two variables that are typically linked by a single or limited set of processes and model structural assumptions. *L* = 1 indicates that the simulated C cycle dynamics behave as a linear system and that ‘source‐control’ dominates its response to increasing GPP under rising CO_2_ (Fatichi et al. 2014, 2019; Körner 2015; Kramer 1981). Thereby, diagnosing *L* distils key model behaviour that can be obtained from commonly available model outputs and derived from observations.

Here, we used TRENDY simulations from CO_2_‐only runs and contrast model‐based *L* to observations‐based values. *L* < 1 suggests an attenuating process along the C cascade—one that dampens the response of downstream pools or fluxes relative to upstream changes (e.g., in GPP). Conversely, *L* > 1 indicates an amplifying, or ‘super‐source driven’ response, where downstream components of the C cycle respond more sensitively to changes in upstream C input.

We conducted the observation‐based calculation of *L* (and its distribution) primarily as a proof of concept to demonstrate the applicability of the method to experimental data. However, the results remain inconclusive because of the limited volume of available observations, their restricted representativeness for the global‐scale changes simulated by models, and the large uncertainty range obtained from the bootstrap analysis. To maximise consistency with the CO_2_‐only model simulations, we applied strict data‐selection criteria and retained only sensitivity estimates derived from experimentally controlled elevated CO_2_ studies (‘eCO_2_’; Walker et al. 2021), which further reduced data availability. Nevertheless, this demonstration illustrates how observational datasets can be analysed in a manner directly comparable to model output, thereby providing process‐level insights into carbon‐cycle dynamics and identifying which links along the carbon cascade contribute to departures from linear dynamics and source control (Fatichi et al. 2014).

We found widely varying patterns in *L* across models (Figure 2a), in particular for the link between NPP and *C*_veg_ (Figure 3d). This indicates that alternative model structural choices lead to diverging *L* among the individual models and that no consensus exists about the representation of key mechanisms that govern the response of the land C cycle to rising CO_2_, consistent with a review of insights gained from Free Air CO_2_ Enrichment (FACE) experiments (Medlyn et al. 2015). In contrast to these earlier works, our analysis covers more links and provides a global picture revealing that there is often a strong variation in model behaviour (measured by *L*) across the globe and different vegetation types and biomes—within individual models (Figures S3, S6, S9, S15, and S18). The clear deviation of *L* from unity for individual models and links indicates an absence of purely source‐driven dynamics in current models.

Previous empirical findings do point out that increases in GPP propagate across the C cascade and increase all ‘downstream’ fluxes (Litton et al. 2007). However, links between fluxes reported by Litton et al. were often not linear. Such non‐linearities could arise, e.g., from the feedback of woody biomass production on respective turnover rates through the tree growth rate‐longevity trade‐off (Brienen et al. 2020; Bugmann and Bigler 2011; Büntgen et al. 2019; Körner 2017). Non‐linearities may also result from changes in NPP partitioning, for example, a shift towards relatively more root production under increasing soil nutrient shortages (Poorter et al. 2012; Stocker et al. 2024), driven by their uptake and immobilisation through enhanced biomass production.

### 4.1 GPP—NPP

The link between GPP and NPP is determined by the response of autotrophic respiration (*R*_a_) and the ecosystem carbon use efficiency (CUE = NPP/GPP) to rising CO_2_. Models calculate NPP from GPP and *R*_a_. Therefore, *L*_NPP:GPP_ is closely related to formulations of the controls on autotrophic respiration (*R*_a_) (Figure S28). *R*_a_ is commonly modelled as consisting of two components—growth and maintenance respiration (Prentice and Cowling 2013). Growth respiration is commonly taken to be proportional to NPP, while maintenance respiration is often taken to be controlled by the total mass of live biomass and modified by temperature and tissue nitrogen concentrations (e.g., LPX‐Bern, LPJ‐GUESS, CABLE‐POP). In a few models (LPJ‐GUESS, CABLE‐POP), leaf maintenance respiration is modelled to be proportional to *V*_cmax_ and (directly or indirectly) to leaf nitrogen content. This link to leaf N introduces a feedback when leaf N concentrations are limited by N availability. Hence, if N availability is simulated to decrease under rising CO_2_—as is often the case in the absence of parallel increases in N deposition or experimental N fertilisation (Stocker et al. 2024; Zaehle et al. 2014), a reduction in the ratio of maintenance respiration (and hence *R*_a_) to GPP is simulated, leading to a CUE increase, and a pattern of *L*_NPP:GPP_ > 1. Note that in our results both GPP and *R*_a_ increase under eCO_2_ (Figure S28). A similar feedback and resulting pattern of *L*_NPP:GPP_ > 1 may arise from CO_2_‐driven reductions in *V*_cmax_ (Leakey et al. 2009) if leaf respiration is formulated as a function of *V*_cmax_. JSBACH calculates maintenance respiration from *V*_cmax_ but not nitrogen content and shows *L*_NPP:GPP_ > 1, implying declines of *V*_cmax_ in the simulation. ORCHIDEE calculates maintenance respiration from nitrogen content but not *V*_cmax_. ORCHIDEE shows *L*_NPP:GPP_ = 0.96 (see also below). JULES calculates maintenance respiration from both *V*_cmax_ and mass based leaf nitrogen concentration but shows *L*_NPP:GPP_ = 0.98 (see also below) (Clark et al. 2011).

We found patterns of *L*_NPP:GPP_ > 1 across most gridcells for CLASSIC, JSBACH, LPJ. Among them, only JSBACH simulates C‐N interactions, indicating that N limitation driving reduced leaf respiration may be an important process driving the results in JSBACH, but is not the dominating effect in all models exhibiting this pattern. It also indicates that reduced autotrophic respiration under elevated CO_2_ may also be simulated in C‐only models (CLASSIC and LPJ). Most C‐only models show *L*_NPP:GPP_ > 1 except ISBA‐CTRIP. In ISBA, biomass allocation is non‐linear, such that CO_2_ fertilisation can promote greater growth in some pools than in others (e.g., leaves; Figure S15). Moreover, respiration is parameterised differently across biomass pools. Together, these effects can cause respiration to increase more strongly than carbon uptake from photosynthesis.

The simulated tendency for *L*_NPP:GPP_ < 1 in several models may be affected by the accounting of C components under N limitation. In C‐N coupled models with widespread *L*_NPP:GPP_ < 1 (DLEM, LPX‐Bern, ORCHIDEE, Figure S1), other nitrogen limitation effects that increase autotrophic respiration outweigh the mechanism explained above. A mismatch of soil N supply and plant N demand is treated in some models by reducing NPP to match N supply under stoichiometric constraints and to divert C for N acquisition, whereby the invested C is accounted as an autotrophic respiration term, thus leading to reduced CUE (e.g., CLM5.0) (Fisher et al. 2010, 201; Lawrence et al. 2019). In other models, such C investments are counted towards allocation to a labile C pool and are thus part of NPP (CABLE‐POP). Similarly, in JSBACH (Reick et al. 2021), NPP is defined before applying N limitation, and therefore likely contributes to its general pattern of *L*_NPP:GPP_ > 1. After applying N limitation, part of NPP is allocated to labile pools, representing root exudates and other non‐structural carbohydrates. The version of JULES in this study let nitrogen limitation reduce *V*_cmax_, GPP, *R*_a_ and NPP, without simulation of non‐structural carbohydrates (Jones et al. 2020). For LPX‐Bern, NPP is limited by the ratio of N demand to N uptake, while autotrophic respiration is not explicitly affected. Taken together, differences between models in their simulation of patterns in *R*_NPP_ vs. *R*_GPP_ are partly related to definition ambiguities in C components that are expended (as part of NPP) or respired (as part of *R*_a_) under N limitation. This results in a diversity of responses in *L*_NPP:GPP_ and its geographical pattern among C‐N coupled models. Hence, a general behaviour of the simulated link between GPP and NPP could not be associated with whether models represented C‐N interactions or not.

Observations from Free‐Air CO_2_ Enrichment (FACE) experiments may provide the most direct test for simulated linearities diagnosed here. However, GPP is often not available from FACE experiments because ecosystem‐scale GPP is difficult to measure under eCO_2_. Instead, light‐saturated CO_2_ assimilation rates (*A*_sat_) have been measured and reported as a proxy for GPP and indicate a higher sensitivity than in NPP (Stocker et al. 2024). Measurements of NPP are not equivalent to models' GPP—*R*_a_ but biomass production, which miss roots exudation. It was reported that the eCO_2_ effect on *A*_sat_ was greater than that on biomass production (Ainsworth and Long 2005; Leakey et al. 2009; Walker et al. 2021). Meanwhile, where GPP was estimated at FACE (EucFACE, POPFACE, DUKE, but no values provided through Walker et al. 2021), the relative increase in NPP was higher than the relative increase in GPP (Gielen et al. 2005; Jiang et al. 2020; Luo, Medlyn, et al. 2001). This appears consistent with the model ensemble that simulated *L*_NPP:GPP_ > 1. However, NPP increases in EucFACE were predominantly used to increase exudates and C export to mycorrhizae (Jiang et al. 2020)—a component that is commonly not explicitly resolved in DGVMs. Plant biomass production increased much less than NPP in EucFACE. Increased C export to mycorrhizae under eCO_2_ is often reported (Fransson 2012; Phillips et al. 2012). At DUKE FACE, measured biomass production increased by 38% (Palmroth et al. 2024) and (Luo, Medlyn, et al. 2001) estimated a 40% increase in GPP by photosynthesis modelling. A revision of model output protocols and the provision of model results for biomass production (excluding allocation to non‐structural C) would enable clearer comparability among models and against experimental results (Walker et al. 2018). A widespread observation in experiments is that *V*_cmax_ declines under elevated CO_2_ (Ainsworth and Rogers 2007; Stocker et al. 2024) which implies a negative response of active Rubisco and may lead to a reduction in leaf respiration, contributing to an increase in CUE and *L*_NPP:GPP_ > 1—consistent with simulations of several models analysed here and presented in the literature (Reich et al. 1998; Wolf et al. 2006; Zaehle and Friend 2010).

### 4.2 NPP—C_veg*_

Two key mechanisms affect the link between NPP and *C*_veg_. First, *L*_Cveg*:NPP_ is affected by changes in allocation of C to different pools with different turnover times—dominated by the distinction between allocation to woody vs. non‐woody biomass (Walker et al. 2019). Second, *L*_Cveg*:NPP_ is affected by changes in the effective turnover time of C in biomass—a reflection of forest stand dynamics, tree demographic rates, self‐thinning (Marqués et al. 2023) and tree size‐dependent mortality (Bennett et al. 2015). Thanks to the consideration of model outputs from TRENDY S1, climate‐driven changes in disturbance and environmental stress‐related tree mortality are excluded by design. Yet, changes in tree mortality (i.e., *C*_veg_ turnover time) may still be affected indirectly by rising CO_2_ through accelerated self‐thinning in vegetation demography‐enabled models (Figure 2) and other models (LPJ, LPX‐Bern) that consider related processes through the crown area ‘packing constraint’ (Marqués et al. 2023; Norby and Zak 2011; Strigul et al. 2008; Walker et al. 2015; Weng et al. 2022; Zaehle and Friend 2010). Moreover, shifts in allocation towards more short‐lived fine root biomass under rising CO_2_ and altered nutrient availability could drive a reduction in effective turnover times in total vegetation C (De Kauwe et al. 2014). Models did not report fine roots and coarse roots separately, which makes this difficult to diagnose. Nonetheless, we observed several models allocate considerably more to *C*_wood_ than *C*_leaf_ + *C*_root_ under eCO_2_ which implies an increase of the effective turnover times in *C*_veg_ (including JULES, CLASSIC, CABLE‐POP and LPJ‐GUESS) and thus theoretically a tendency for *L*_Cveg*:NPP_ > 1 (Figure 2a).

Across models, the relationship between *R*_NPP_ and *R*_Cveg*_ was much weaker compared to the other two links investigated here. Yet, the average response across all models and gridcells pooled still indicates a tendency towards a predominantly linear relationship with the distribution of *L*_Cveg*:NPP_ peaking around 1.0 (Figure 3). This indicates that, across this model ensemble, a change in NPP elicits a relatively wide range of changes in steady‐state *C*_veg_, but on average across the globe and the model ensemble, a linear relationship dominates. A secondary peak of the *L*_Cveg*:NPP_ distribution around 0 (Figure S24) indicates that no vegetation biomass changes are simulated despite NPP increases. This pattern occurs mostly in arid and grassland regions (Figure S6) (Figure 3b compared to Figure S24b) and likely reflects the behaviour of annual herbaceous vegetation in which NPP increases do not lead to sustained *C*_veg_ gains.

For several models, we found *L*_Cveg*:NPP_ around unity or slightly above across forest regions (ISBA‐CTRIP, ORCHIDEE, VISIT, VISIT‐NIES), indicating no or compensating responses in effective biomass turnover rates and mortality and allocation to short vs. long‐lived biomass pools (Figure S6). Several models that consider a ‘packing constraint’ for simulating tree size‐density relationships (LPJ, LPX‐Bern) exhibit general patterns of *L*_Cveg*:NPP_ < 1 across tropical forest regions (Figure S6). In LPJ‐GUESS, this packing constraint is partly relieved by allowing tree crown overlaps (Walker et al. 2015), which may result in higher *L*_Cveg*:NPP_ values than in the genealogically related models LPJ and LPX‐Bern. In IBIS, a general pattern of *L*_Cveg*:NPP_ < 1 may be linked to a smaller increase in woody biomass than in total biomass (Figure S16). Simulated patterns of *L*_Cveg*:NPP_ < 1 may also arise from structural limits of tree sizes and ensuing mortality that are reached more often as NPP increases (JSBACH). In contrast, SDGVM assumes a positive relationship between the wood allocation fraction and NPP (Walker et al. 2015) and exhibits a general pattern with *L*_Cveg*:NPP_ > 1 across forest regions. Additionally, CABLE‐POP simulates more carbon allocation to total vegetation than to leaves (Figure 2a) and exhibits a dominant *L*_Cveg*:NPP_ > 1. General patterns of *L*_Cveg*:NPP_ are linked to model structural assumptions. Nonetheless, other mechanisms could be at play and simulated patterns are likely affected by simulated vegetation states (e.g., tree density and size). Therefore, direct causal effects between model structure and diagnosed linearity could be better interpreted if additional model outputs were made available (e.g., NPP and C stocks by vegetation compartment; tree mortality).

Although observation‐based compilation shows a median *L*_Cveg:NPP_ < 1, data behind this result are sparse. Furthermore, field observed *C*_veg_ was not corrected for steady state so it is challenging to assess how well the simulated patterns align with empirical evidence for *L*_Cveg*:NPP_. The direction of *L*_Cveg*:NPP_ is currently inconclusive because multiple processes underlie *L*_Cveg:NPP_ and the literature lacks consensus on these processes (discussed below) (Table S1). A pattern of *L*_Cveg*:NPP_ < 1 in forest regions appears to be supported by the observation that fast‐growing trees tend to live shorter than slow‐growing trees (Brienen et al. 2020; Bugmann and Bigler 2011; Büntgen et al. 2019; Locosselli et al. 2020). However, these relationships predominantly emerge from spatial or interspecific variations (Locosselli et al. 2020; Walker et al. 2021), not from temporal changes in response to eCO_2_. Yet, one long‐term forest monitoring study found little change in forest self‐thinning relations despite accelerated growth, thus supporting *L*_Cveg*:NPP_ < 1 (Pretzsch et al. 2014). In contrast, an analysis of FACE experiments in relatively young forests indicates increases in wood allocation with increasing NPP (Walker et al. 2019), thus supporting *L*_Cveg*:NPP_ > 1. Meanwhile, some literature suggested *L*_Croot:Cveg_ > 1 (discussed below) which support *L*_Cveg*:NPP_ < 1. Nonetheless vegetation demography models overall simulate *L*_Cveg*:NPP_ = 1.2 (Figure 2c), in contrast to the theoretical expectation from considerations of forest dynamics and self‐thinning relations. The representation of vegetation demography should reduce *L*_Cveg*:NPP_, but this signal may be masked by other processes, for example, an increase in *C*_veg_ turnover time due to relatively more allocation to woody biomass than leaves. Future studies are needed to investigate the relative contribution of different mechanisms that affect the link between NPP and the vegetation C pool and to use diverse observations for estimating linearity in the framework applied here.

### 4.3 C_veg_—C_root_

The link between *C*_veg_ and *C*_root_ is determined by allocation and whether the effective turnover rates in these pools change under rising CO_2_. Unless effective turnover rates change, an increased fraction of NPP allocated to root biomass will yield *L*_Croot:Cveg_ > 1. The median of *L*_Croot:Cveg_ was larger than 1 based on experimental observations (Figure 5). This is consistent with the general pattern from ecosystem experiments for such a shift towards more belowground allocation under elevated CO_2_ (Ainsworth and Long 2005; Chen et al. 2026; De Kauwe et al. 2014; Jiang et al. 2020; Leakey et al. 2009; H. H. Rogers et al. 1995; Schneider et al. 2004; Song et al. 2019; Terrer et al. 2021) and that the relative increase in *C*_root_ is greater than the relative increase in *C*_veg_ (Stocker et al. 2024) (Table S1). This response has been interpreted in light of the functional balance hypothesis (Bloom et al. 1985; Franklin 2007; Rastetter et al. 1997) which predicts a shift towards more belowground allocation as the balance between CO_2_ assimilation efficiency (C uptake per unit aboveground biomass) and soil nutrient acquisition efficiency (nutrient acquisition per unit root biomass) increases under rising CO_2_ (Stocker et al. 2024). However, dynamics in response to an experimental step‐change in CO_2_ likely differ from dynamics in response to a gradual rise, extended over decadal to centennial time scales as simulated in TRENDY runs. In models that simulate a demand‐dependency of N uptake, previous multi‐model evaluations against FACE experiments found a gradual relaxation from N limitation over time (Walker et al. 2015). Hence, the gradual rise in CO_2_ may trigger a weaker nitrogen limitation effect and associated shifts in allocation, and a reduced tendency for *L*_Croot:Cveg_ > 1.

On average across the ensemble of models investigated here, the pattern towards increased belowground allocation is not generally simulated. Contrary to expectations, C–N models simulate lower *L*_Croot:Cveg_ than C‐only models. Only ISAM, IBIS, and VISIT‐NIES simulated a clear tendency for *L*_Croot:Cveg_ > 1. ISAM accounts for C‐N cycling and a plastic response of root growth to soil N supply and demand (Meiyappan et al. 2015), which likely led to the clear pattern of *L*_Croot:Cveg_ > 1. However, other models that exhibited *L*_Croot:Cveg_ > 1 (IBIS, VISIT‐NIES) did not resolve N cycling, and models that did resolve C‐N interactions simulated *L*_Croot:Cveg_ < 1 for a clear majority of gridcells (JULES, ORCHIDEE). JULES's *C*_root_ is fine root without coarse root (Figure S23) and *C*_root_ = *C*_leaf_ was fixed (Figure S30) (Clark et al. 2011). This appears to lead to a disagreement with field observations Figure 4 (Table S1). Several models simulated predominantly equal relative changes in total vegetation biomass and root biomass. The distribution of *L*_Croot:Cveg_ is most narrowly constrained around unity for IBIS, CABLE‐POP and SDGVM, and ORCHIDEE (shown as a tall peak in Figure S7). This pattern likely reflects the most common model structural choice whereby fixed allocation is considered per plant functional type. For example, ORCHIDEE considers a fixed stem‐to‐coarse root ratio following the ‘pipe model’ concept (Goll et al. 2018). Note that *C*_root_ of ORCHIDEE includes coarse roots (Figure S23). CABLE‐POP considers a fixed allocation fraction to fine roots (Haverd et al. 2018) (Figure S7). In IBIS, the distribution of *L*_Croot:Cveg_ exhibits two modes and it appears that root allocation in forest regions is governed differently, leading to *L*_Croot:Cveg_ > 1, compared to other regions, where *L*_Croot:Cveg_ is near unity (Figure S9). Substantial between‐model structural differences exist also regarding allocation to *C*_leaf_ and *C*_wood_ (Figures S13–S18). We note that the interpretation of *L*_Croot:Cveg_ patterns in relation to changes in root allocation should be approached with caution, as concurrent shifts in effective turnover rates can obscure or counteract these patterns. Additional model output variables, most importantly plant compartment‐specific NPP, would enable a clearer interpretation of simulated mechanisms.

### 4.4 Limitations and Sensitivity Test

Diagnosing linearity in responses of the land C cycle is challenging because available simulation outputs do not represent steady states under different levels of CO_2_ but instead reflect the transient response. We applied an estimation of the steady state from transient simulations (Equation 14). In future, purposely designed model simulations (e.g., a step‐increase in CO_2_ and a subsequent equilibration to a new steady‐state), and model outputs on plant compartment‐specific NPP would facilitate diagnosing system dynamics and the comparison to ecosystem CO_2_ experiments. Nonetheless, this study presents several sensitivity tests regarding the estimation of the steady state. We performed an evaluation of the reliability of this steady state‐estimation method (Equation 14) using three demonstration vegetation models (Figures S30–S42). We found that (i) the steady‐state correction yields a more accurate description of the actual steady state (which is not available from TRENDY v11 outputs) and hence a more accurate quantification of the linearity term *L* for all models. (ii) The correction does not imply a diagnosed linear behaviour for models that do not follow linear dynamics and the qualitative information of *L* > 1 or *L* < 1 is insensitive to the correction. (iii) The quantitative effect of applying the correction on non‐linear models is minor. To visualise the effect, we have also shown results for *L*_Cveg:NPP_ (Figures S19–S21) in addition to *L*_Cveg*:NPP_ (Figures 5 and S4–S6). These two sets of results delivered similar patterns in *L* while *L*_Cveg*:NPP_ is slightly larger than *L*_Cveg:NPP_. The finding of a wide diversity in *L*_CVeg*:NPP_, also seen for *L*_CVeg:NPP_, (Figures S19–S21); and the finding of a relatively narrow clustering of *L*_CRoot:CVeg_ values around unity, is also seen for *L*_CRoot:CVeg*_, (Figures S10–S13). We also note that the same procedure for estimating steady‐state pool sizes was applied to outputs from all models, so inter‐model comparison is not affected by steady‐state correction. We have also shown the difference between the relative change of total vegetation C with steady state correction (*R*_*C*veg_*) and without correction (*R*_*C*veg_) (Figures S26 and S27). *R*_*C*veg_/*R*_*C*veg_* is around 0.8–0.9, and is relatively uniform across models and spatially.

Our approach to diagnosing system dynamics from available transient model outputs enables comparisons to diverse observations in the future and may guide future model development and evaluation towards a focus on capturing essential and widely observed patterns in the C cycle dynamics. Currently, the field observations‐based pattern is unclear for *L*_NPP:GPP_ and *L*_Cveg*:NPP_. Considering the difficulties in simultaneously measuring GPP and NPP in CO_2_ experiments, data availability may constitute a persistent limitation for evaluating models' representation of respective processes.

Furthermore, for the *L* terms quantified on links that include pools (here *L*_Cveg*:NPP_ and *L*_Croot:Cveg*_), interpretations have to consider that both changes in relative allocation between compartments with widely varying turnover rates and compartment‐specific turnover rates (affected by continuous turnover and by more episodic tree mortality, phenology and leaf senescence) can influence results. This obscures interpretations with a direct view to process representations in the models. Additional model outputs (e.g., compartment‐specific NPP) could be used to better inform analyses in this respect.

### 4.5 Conclusions

Based on field observations, the ‘source‐driven’ paradigm of land carbon dynamics was criticised (Fatichi et al. 2014, 2019). Several additional processes have been implemented in models and suggest a stronger importance of ‘sink‐driven’ controls (e.g., nutrient limitations). Indeed, we found that individual models deviate clearly from linear, source‐driven dynamics and stark global variations of the degree of non‐linearity—especially for the relationship between NPP and vegetation C stock changes (Figure S6).

However, our analysis did not yield any support that this deviation from linear dynamics is a reflection of representations of individual processes that have been argued to imply sink control and deviation from linear dynamics (Fatichi et al. 2014), including N limitation and vegetation demography (Figure 2b,c). These non‐linear formulations in the model did lead to non‐linear behaviour at different geographical locations, but they combine to approximate linearity. We found the strongest inter‐model disagreement for the link between vegetation C and NPP (Figure 3d)—indicating a need for model benchmarking with a focus on related processes—including the tree growth‐longevity feedback, forest demographic processes, and CO_2_‐driven changes in allocation to tissues with widely differing turnover times.

## Author Contributions

**Peter Anthoni:** data curation, methodology, validation, writing – review and editing. **Vivek K. Arora:** methodology, validation, data curation, writing – review and editing. **Julia Pongratz:** methodology, validation, writing – review and editing, data curation. **Sönke Zaehle:** methodology, validation, writing – review and editing, data curation. **Julia Nabel:** methodology, validation, writing – review and editing, data curation. **Benjamin Poulter:** methodology, validation, writing – review and editing, data curation. **Daniel S. Goll:** methodology, validation, writing – review and editing, data curation. **Thomas A. M. Pugh:** methodology, validation, writing – review and editing, data curation. **Atul K. Jain:** methodology, validation, writing – review and editing, data curation. **Wenping Yuan:** methodology, validation, writing – review and editing, data curation. **Yadvinder Malhi:** methodology, validation, writing – review and editing, data curation. **Huanyuan Zhang‐Zheng:** methodology, investigation, funding acquisition, writing – original draft, writing – review and editing, visualization, validation, software, formal analysis, project administration, resources, data curation. **Anthony P. Walker:** methodology, validation, writing – review and editing, data curation. **Jürgen Knauer:** methodology, validation, writing – review and editing, data curation. **Etsushi Kato:** methodology, validation, writing – review and editing, data curation. **Akihiko Ito:** methodology, validation, writing – review and editing, data curation. **Naiqing Pan:** methodology, validation, writing – review and editing, data curation. **Michael O'Sullivan:** methodology, validation, writing – review and editing, data curation. **Minxue Tang:** software, formal analysis, validation, visualization, writing – review and editing. **Hanqin Tian:** methodology, validation, writing – review and editing, data curation. **Pierre Friedlingstein:** methodology, validation, writing – review and editing, data curation. **Jeanne Decayeux:** methodology, validation, writing – review and editing, data curation. **Benjamin D. Stocker:** methodology, validation, writing – review and editing, data curation, conceptualization, investigation, funding acquisition, visualization, formal analysis, software, project administration, resources, supervision. **Qing Sun:** methodology, validation, writing – review and editing, data curation. **Stephen Sitch:** methodology, validation, writing – review and editing, data curation. **Ruijie Ding:** writing – review and editing, software, formal analysis, validation, visualization. **César Terrer:** methodology, validation, writing – review and editing, data curation.

## Funding

This work was supported by the U.S. Department of Energy, Next Generation Ecosystem Experiments‐Tropics, Swiss National Science Foundation (Grant PCEFP2_181115) and Swedish research council for sustainable development (Grant 2023‐00361). H.Z.‐Z. was supported as part of the Next Generation Ecosystem Experiments‐Tropics, funded by the U.S. Department of Energy, Office of Science, Office of Biological and Environmental Research. JSBACH simulations are performed using resources of the Deutsches Klimarechenzentrum (DKRZ) granted by its Scientific Steering Committee (WLA) under project 891. A.I. was supported by JSPS KAKENHI (grant no. 21H05318). Q.S. was supported by the Swiss National Science Foundation (200020 200511). Simulations of LPX‐Bern were performed on UBELIX (https://www.id.unibe.ch/hpc), the HPC cluster at the University of Bern. T.A.M.P. was funded under the European Union‘s Horizon 2020 programme (grant agreement no. 758873, TreeMort) and Horizon Europe (101141836, Tree2Globe). This study is a contribution to the Swedish government‘s strategic research areas BECC and MERGE and the Nature‐based Future Solutions profile area at Lund University. This study is supported by Swedish research council for sustainable development (grant: 2023‐00361). ORNL is managed by UT‐Battelle LLC, for the DOE under contract DE‐AC05‐1008 00OR22725. B.D.S. acknowledges funding from the Swiss National Science Foundation grant PCEFP2_181115. J.K. acknowledges support from the UTS Chancellor‘s Research Fellowship.

## Conflicts of Interest

The authors declare no conflicts of interest.

## Supporting information


**Table S1:** Field evidence on linearity. Please note that subscript is not used to enhance visibility.
**Figure S1:** Density of the distribution of LNPP:GPP values across gridcells by model, printed as labels on top of each panel. ‘All’ shows cross models overall pattern, identical to the figure presented in the main text. The proportion of gridcells with LNPP:GPP < 1 (LNPP:GPP > 1) is given by the annotation on the left (right) side of the plotting area. The panel labelled ‘ALL’ represents the joint pattern with data pooled from all models. LNPP:GPP = RNPP/RGPP where RNPP (RGPP) is the relative change of net primary productivity, NPP (gross primary production, GPP), evaluated from simulations with rising CO2 and changing N deposition. Both L and R terms are unitless. We tagged models as carbon‐nitrogen coupled models (C‐N) and vegetation demography models (Veg.D.).
**Figure S2:** Density scatter plot of RNPP vs. RGPP values across all gridcells for each model. The name of the model is printed on top of each histogram. Dark colors denote high density, while bright colors denote low density. RNPP is the relative change (ΔNPP/NPP, unitless) of net primary productivity (NPP) evaluated from simulations with rising CO2 and changing N deposition. RGPP is the relative change (ΔGPP/GPP, unitless) of gross primary productivity (GPP), evaluated from the same simulations. The panel labelled ‘ALL’ represents the joint pattern with data pooled from all models. We tagged models as carbon‐nitrogen coupled models (C‐N) and vegetation demography models (Veg.D.).
**Figure S3:** Spatial pattern of LNPP:GPP for each model. The name of each model is printed on top of each map. LNPP:GPP = RNPP/RGPP where RNPP (RGPP) is the relative change of net primary productivity, NPP (gross primary production, GPP), evaluated from simulations with rising CO2 and changing N deposition. Both L and R terms are unitless. We tagged models as carbon‐nitrogen coupled models (C‐N) and vegetation demography models (Veg.D.).
**Figure S4:** Density of the distribution of LCveg*:NPP values across gridcells by model, printed as labels on top of each panel. The proportion of gridcells with LCveg*:NPP < 1 (LCveg*:NPP > 1) is given by the annotation on the left (right) side of the plotting area. The panel labelled ‘ALL’ represents the joint pattern with data pooled from all models. LCveg*:NPP = RCveg*/RNPP where RNPP (RCveg) is the relative change of net primary productivity, NPP (steady‐state total vegetation C pools), evaluated from simulations with rising CO2 and changing N deposition. Both L and R terms are unitless. We tagged models as carbon‐nitrogen coupled models (C‐N) and vegetation demography models (Veg.D.).
**Figure S5:** Density scatter plot of RNPP vs. RCveg* values across all gridcells for each model. The name of the model is printed on top of each histogram. Dark colors denote high density, while bright colors denote low density. RNPP is the relative change (ΔNPP/NPP, unitless) of net primary productivity (NPP) evaluated from simulations with rising CO2 and changing N deposition. RCveg* is the relative change (ΔCveg*/Cveg*, unitless) of total vegetation carbon, evaluated from the same simulations. The panel labelled ‘ALL’ represents the joint pattern with data pooled from all models. We tagged models as carbon‐nitrogen coupled models (C N) and vegetation demography models (Veg.D.).
**Figure S6:** Spatial pattern of LCveg*:NPP for each model. The name of each model is printed on top of each map. LCveg*:NPP = RCveg*/RNPP where RCveg* is the relative change of the steady state estimate of total vegetation carbon, and RNPP is the relative change in net primary production (NPP), evaluated from simulations with rising CO2 and changing N deposition. Both L and R terms are unitless.
**Figure S7:** Density of the distribution of LCroot:Cveg values across gridcells by model, printed as labels on top of each panel. The proportion of gridcells with LCroot:Cveg < 1 (LCroot:Cveg > 1) is given by the annotation on the left (right) side of the plotting area. The panel labelled ‘ALL’ represents the joint pattern with data pooled from all models. LCroot:Cveg = RCroot/RCveg where RCroot (RCveg) are the relative change of root C (total vegetation C), evaluated from simulations with rising CO2 and changing N deposition. Both L and R terms are unitless. We tagged models as carbon‐nitrogen coupled models (C‐N) and vegetation demography models (Veg.D.).
**Figure S8:** Density scatter plot of RCroot vs. RCveg values across all gridcells for each model. The name of the model is printed on top of each histogram. Dark colors denote high density, while bright colors denote low density. RCroot is the relative change (ΔCroot/Croot, unitless) of root C. RCveg is the relative change (ΔCveg/Cveg, unitless) of total vegetation C. Both relative changes are evaluated from the same simulations. The panel labelled ‘ALL’ represents the joint pattern with data pooled from all models. We tagged models as carbon‐nitrogen coupled models (C‐N) and vegetation demography models (Veg.D.).
**Figure S9:** Spatial pattern of LCroot:Cveg for each model. The name of each model is printed on top of each map. LCroot:Cveg = RCroot/RCveg where RCroot (RCveg) is the relative change in root C (total vegetation C), evaluated from simulations with rising CO2 and changing N deposition. Both L and R terms are unitless. We tagged models as carbon‐nitrogen coupled models (C‐N) and vegetation demography models (Veg.D.).
**Figure S10:** Density of the distribution of LCroot:Cveg* values across gridcells by model, printed as labels on top of each panel. The proportion of gridcells with LCroot:Cveg* < 1 (LCroot:Cveg* > 1) is given by the annotation on the left (right) side of the plotting area. The panel labelled ‘ALL’ represents the joint pattern with data pooled from all models. LCroot:Cveg* = RCroot/RCveg* where RCroot are the relative change of root C (steady‐state total vegetation C), evaluated from simulations with rising CO2 and changing N deposition. Both L and R terms are unitless. As many models include coarse roots in Croot, Croot should not have rapid response to carbon fertilisation. Ideally, we should apply steady state conversion to Croot, and calculate LCroot*:Cveg* but this could not be implemented due to the lack of Croot turnover time. We believe that LCroot:Cveg* is less useful than LCroot:Cveg.
**Figure S11:** Density scatter plot of RCroot vs. RCveg* values across all gridcells for each model. The name of the model is printed on top of each histogram. Dark colors denote high density, while bright colors denote low density. RCroot is the relative change (ΔCroot/Croot, unitless) of root C. RCveg* is the relative change (ΔCveg*/Cveg, unitless) of steady‐state total vegetation C. Both relative changes are evaluated from the same simulations. The panel labelled ‘ALL’ represents the joint pattern with data pooled from all models. As many models include coarse roots in Croot, Croot should not have rapid response to carbon fertilisation. Ideally, we should apply steady state conversion to Croot, and calculate LCroot*:Cveg* but this could not be implemented due to the lack of Croot turnover time. We believe that LCroot:Cveg* is less useful than LCroot:Cveg (Figure S8).
**Figure S12:** Spatial pattern of LCroot:Cveg* for each model. The name of each model is printed on top of each map. LCroot:Cveg* = RCroot/RCveg* where RCroot (RCveg*) is the relative change in root C (steady‐state total vegetation C), evaluated from simulations with rising CO2 and changing N deposition. Both L and R terms are unitless. As many models include coarse roots in Croot, Croot should not have rapid response to carbon fertilisation. Ideally, we should apply steady state conversion to Croot, and calculate LCroot*:Cveg* but this could not be implemented due to the lack of Croot turnover time. We believe that LCroot:Cveg* is less useful than LCroot:Cveg (Figure S8).
**Figure S13:** Density of the distribution of LCleaf:Cveg* values across gridcells by model, printed as labels on top of each panel. The proportion of gridcells with LCleaf:Cveg* < 1 (LCleaf:Cveg* > 1) is given by the annotation on the left (right) side of the plotting area. The panel labelled ‘ALL’ represents the joint pattern with data pooled from all models. LCleaf:Cveg* = RCleaf/RCveg* where RCleaf are the relative change of leaf C (steady‐state total vegetation C), evaluated from simulations with rising CO2 and changing N deposition. We tagged models as carbon‐nitrogen coupled models (C‐N) and vegetation demography models (Veg.D.).
**Figure S14:** Density scatter plot of RCleaf vs. RCveg* values across all gridcells for each model. The name of the model is printed on top of each histogram. Dark colors denote high density, while bright colors denote low density. RCleaf is the relative change (ΔCleaf/Cleaf, unitless) of leaf C. RCveg* is the relative change (ΔCveg*/Cveg, unitless) of steady‐state total vegetation C. Both relative changes are evaluated from the same simulations. The panel labelled ‘ALL’ represents the joint pattern with data pooled from all models. We tagged models as carbon nitrogen coupled models (C‐N) and vegetation demography models (Veg.D.).
**Figure S15:** Spatial pattern of LCleaf:Cveg* for each model. The name of each model is printed on top of each map. LCleaf:Cveg* = RCleaf/RCveg* where RCleaf (RCveg*) is the relative change in leaf C (steady‐state total vegetation C), evaluated from simulations with rising CO2 and changing N deposition. Both L and R terms are unitless. We tagged models as carbon nitrogen coupled models (C‐N) and vegetation demography models (Veg.D.).
**Figure S16:** Density of the distribution of LCwood:Cveg values across gridcells by model, printed as labels on top of each panel. The proportion of gridcells with LCwood:Cveg < 1 (LCwood:Cveg > 1) is given by the annotation on the left (right) side of the plotting area. The panel labelled ‘ALL’ represents the joint pattern with data pooled from all models. LCwood:Cveg = RCwood/RCveg where RCwood (RCveg) are the relative change of wood C (total vegetation C), evaluated from simulations with rising CO2 and changing N deposition. Both L and R terms are unitless. We tagged models as carbon‐nitrogen coupled models (C‐N) and vegetation demography models (Veg.D.).
**Figure S17:** Density scatter plot of RCwood vs. RCveg values across all gridcells for each model. The name of the model is printed on top of each histogram. Dark colors denote high density, while bright colors denote low density. RCwood is the relative change (ΔCwood/Cwood, unitless) of wood C. RCveg is the relative change (ΔCveg/Cveg, unitless) of total vegetation C. Both relative changes are evaluated from the same simulations. The panel labelled ‘ALL’ represents the joint pattern with data pooled from all models. We tagged models as carbon nitrogen coupled models (C‐N) and vegetation demography models (Veg.D.).
**Figure S18:** Spatial pattern of LCwood:Cveg for each model. The name of each model is printed on top of each map. LCwood:Cveg = RCwood/RCve* where RCwood (RCveg) is the relative change in wood C (steady‐state total vegetation C), evaluated from simulations with rising CO2 and changing N deposition. Both L and R terms are unitless. We tagged models as carbon nitrogen coupled models (C‐N) and vegetation demography models (Veg.D.).
**Figure S19:** Similar to Figure S4. The only difference is the steady state estimation of Cveg* in Figure S4 but not in this figure (see method). The figure is provided for visualising the effect of steady state estimation. We are certain that Figure S4 is more reasonable than this one.
**Figure S20:** Similar to Figure S5. The only difference is the steady state estimation of Cveg* in Figure S5 but not in this figure (see method). The figure is provided for visualising the effect of steady state estimation. We are certain that Figure S5 is more reasonable than this one.
**Figure S21:** Similar to Figure S6. The only difference is the steady state estimation of Cveg* in Figure S6 but not in this figure (see method). The figure is provided for visualising the effect of steady state estimation. We are certain that Figure S6 is more reasonable than this one.
**Figure S22:** Similar to Figure 5. The only difference is the steady state estimation of Cveg* in Figure 5 but not in this figure (see method). The figure is provided for visualising the effect of steady state estimation. We are certain that Figure 5 is more reasonable than this one.
**Figure S23:** Root biomass (Croot) divided by vegetation biomass (Cveg) for each model. This figure is used to diagnose which model includes coarse roots in output variable Croot. The idea is that for tropical evergreen forests, Croot/Cveg > 5% would signal that coarse root is included in Croot. We did try to find out whether the model outputs of Croot include coarse roots, by reading manuals and contracting modelers. However, some modelers just can't remember or they are not responsible for roots simulation. Such information may be archived somewhere in the output explanation of the model manual. In this case, the only risk is that the model manual was not written for TRENDY and the TRENDY outputs could be in a different format. If unfortunately not explained in the output part, it would be somewhere in the codes or equations which will take a whole day to find out (an example of model opaqueness). In any case, the most reliable and efficient way to find out, is by diagnosing the output and draw Croot/Cveg.
**Figure S24:** The relationship between the relative change of steady‐state vegetation C (RCveg* = ΔCveg*/Cveg) and the relative change of net primary productivity (RNPP = ΔNPP/NPP). Same figure as Figure 5 (in the main text) but here we include gridcells with Cveg lower than 5% quantile (i.e., include sparse desert). By comparing with Figure 5, it could be concluded that small Cveg values lead to a spike at of LCveg*:NPP = 0.
**Figure S25:** Redo Figure 1 with the period 2008–2017 instead of 2013–2022, while keeping everything else unchanged. This is done as a sensitivity test to check the effect of chosen time period. This figure is very similar to Figure 1, implying that the chosen time period would not affect emergent patterns in L.
**Figure S26:** Difference between the relative change of total vegetation C with steady state correction (RCveg*) and without correction (RCveg).
**Figure S27:** Difference between the relative change of total vegetation C with steady state correction (RCveg*) and without correction (RCveg).
**Figure S28:** Relative change in autotrophic respiration (Ra).
**Figure S29:** Redo Figures S16 and S17 but with steady state correction for Cwood. We can only do this for JULES only because of the availability of relevant output variables.
**Figure S30:** Redo Figures S7 and S8 but with steady state correction for Croot. We can do this for JULES only because of the availability of relevant output variables. None of the TRENDY v11 models provided NPProot or NPPwood so steady state correction for root and wood is not possible. However, JULES provides fWoodLitter (which is the carbon flux leaving the wood pool) and fRootLitter (which is the carbon flux leaving the root pool). Therefore we can get NPProot using:
**Figure S31:** Simulated vegetation C pool size (orange line) in response to transiently changing CO2 after 1700 up to 2018 and the estimated steady state pool size, derived from transiently changing simulation outputs (black line). CO2 is held constant for 1000 years after 2018.
**Figure S32:** Difference between the actual steady state vegetation C pool and the estimated value, derived from transient model outputs.
**Figure S33:** Difference between the actual steady state vegetation C pool and the estimated value, derived from transient model outputs.
**Figure S34:** Linearity term L derived for the linear model (ccascade) from (uncorrected) transient outputs (blue), from the estimated steady‐state (orange), and from the actual steady state (black).
**Figure S35:** Simulated vegetation C pool size (orange line) in response to transiently changing CO2 after 1700 up to 2018 and the estimated steady state pool size, derived from transiently changing simulation outputs (black line). CO2 is held constant for 1000 years after 2018.
**Figure S36:** Difference between the actual steady state vegetation C pool and the estimated value, derived from transient model outputs.
**Figure S37:** Difference between the actual steady state vegetation C pool and the estimated value, derived from transient model outputs.
**Figure S38:** Linearity term L derived for the linear model (ccascade) from (uncorrected) transient outputs (blue), from the estimated steady‐state (orange), and from the actual steady state (black).
**Figure S39:** Simulated vegetation C pool size (orange line) in response to transiently changing CO2 after 1700 up to 2018 and the estimated steady state pool size, derived from transiently changing simulation outputs (black line). CO2 is held constant for 1000 years after 2018.
**Figure S40:** Difference between the actual steady state vegetation C pool and the estimated value, derived from transient model outputs.
**Figure S41:** Difference between the actual steady state vegetation C pool and the estimated value, derived from transient model outputs.
**Figure S42:** Linearity term L derived for the non‐linear model (BiomeEP) from (uncorrected) transient outputs (blue), from the estimated steady‐state (orange), and from the actual steady state (black).

**Supporting Information: 1** Median and inter‐quantile range (IQR) for each relative change and linearity term. This table is inserted here as a screenshot to remind readers that a full table of L and R is available as Supporting Information.

## Data Availability

Model outputs from TRENDY v11 simulations were obtained from https://mdosullivan.github.io/GCB/. Codes and data to reproduce figures are available on Zenodo at the following link: https://doi.org/10.5281/zenodo.20720993. The project may further develop after publication which could be tracked at https://github.com/geco‐bern/dgvmlin. To reproduce results, please run vignettes/DGVMLIN_trendy_v11.rmd and analysis/calc_walker21_relative_changes.R. Values for the *R* and *L* terms are available as Supporting Information on *Global Change Biology* webpage.
