# Supplementary material for: Diagnosing Linearity Along the Carbon Cascade in Terrestrial Biosphere Models and Observations
Source: Glob Chang Biol. 2026 Jul 26;32(7):e70982. doi: 10.1111/gcb.70982 (PMC13401867; doi:10.1111/gcb.70982)
Supplement: Supplementary file 1 — Table S1: Field evidence on linearity. Please note that subscript is not used to enhance visibility. Figure S1: Density of the distribution of LNPP:GPP values across gridcells by model, printed as labels on top of each panel. ‘All’ shows cross models overall pattern, identical to the figure presented in the main text. The proportion of gridcells with LNPP:GPP < 1 (LNPP:GPP > 1) is given by the annotation on the left (right) side of the plotting area. The panel labelled ‘ALL’ represents the joint pattern with data pooled from all models. LNPP:GPP = RNPP/RGPP where RNPP (RGPP) is the relative change of net primary productivity, NPP (gross primary production, GPP), evaluated from simulations with rising CO2 and changing N deposition. Both L and R terms are unitless. We tagged models as carbon‐nitrogen coupled models (C‐N) and vegetation demography models (Veg.D.). Figure S2: Density scatter plot of RNPP vs. RGPP values across all gridcells for each model. The name of the model is printed on top of each histogram. Dark colors denote high density, while bright colors denote low density. RNPP is the relative change (ΔNPP/NPP, unitless) of net primary productivity (NPP) evaluated from simulations with rising CO2 and changing N deposition. RGPP is the relative change (ΔGPP/GPP, unitless) of gross primary productivity (GPP), evaluated from the same simulations. The panel labelled ‘ALL’ represents the joint pattern with data pooled from all models. We tagged models as carbon‐nitrogen coupled models (C‐N) and vegetation demography models (Veg.D.). Figure S3: Spatial pattern of LNPP:GPP for each model. The name of each model is printed on top of each map. LNPP:GPP = RNPP/RGPP where RNPP (RGPP) is the relative change of net primary productivity, NPP (gross primary production, GPP), evaluated from simulations with rising CO2 and changing N deposition. Both L and R terms are unitless. We tagged models as carbon‐nitrogen coupled models (C‐N) and vegetation demography models (Veg.D. [file GCB-32-e70982-s001.pdf]

Supplementary information for

Diagnosing linearity along the carbon cascade in terrestrial biosphere models

Huanyuan Zhang-Zheng [1,2,3], Vivek K. Arora [4], Peter Anthoni [5], Thomas A. M. Pugh [6,7,8], Atul K. Jain [9], Wenping Yuan [10], Yadvinder Malhi [1,2], Julia Nabel [11,12], Daniel S. Goll [13], Julia Pongratz [14,12], Benjamin Poulter [15,16], Anthony P. Walker [17], Sönke Zaehle [11], Jürgen Knauer [18,19], Etsushi Kato [20], Ruijie Ding [21], Minxue Tang [21], Stephen Sitch [22], Michael O'Sullivan [22], César Terrer [23], Hanqin Tian [24,25], Naiqing Pan [24], Pierre Friedlingstein [22,26], Akihiko Ito [27], Qing Sun [28,29,30], Jeanne Decayeux [31], Benjamin D. Stocker [32,33]*

* Corresponding Author (benjamin.stocker@unibe.ch)

1 Environmental Change Institute, School of Geography and the Environment, University of Oxford, Oxford, United Kingdom
2 Leverhulme Centre for Nature Recovery, University of Oxford, Oxford, UK
3 Department of Ecology, Environment and Geoscience, Umeå University, 901 87 Umeå, Sweden
4 Canadian Centre for Climate Modelling and Analysis, Environment and Climate Change Canada, Victoria, Canada
5 Institute of Meteorology and Climate Research/Atmospheric Environmental Research, Karlsruhe Institute of Technology, 82467 Garmisch-Partenkirchen, Germany
6 Department of Physical Geography and Ecosystem Science, Lund University, Lund, Sweden
7 Department of Geography, Earth and Environmental Sciences, University of Birmingham, UK
8 Birmingham Institute of Forest Research, University of Birmingham, UK
9 Department of Climate, Meteorology, and Atmospheric Sciences (CLiMAS), University of Illinois Urbana-Champaign, Urbana, IL 61801, USA
10 Institute of Carbon Neutrality, Sino-French Institute for Earth System Science, College of Urban and Environmental Sciences, Peking University, Beijing, China
11 Max Planck Institute for Biogeochemistry, Jena, Germany
12 Max Planck Institute for Meteorology, Hamburg, Germany
13 Laboratoire des Sciences du Climat et de l'Environnement, CEA-CNRS-UVSQ-Université Paris-Saclay, Gif-sur-Yvette, France
14 Ludwig-Maximilians-Universität München, Department of Geography, München, Germany
15 Spark Climate Solutions, San Francisco, CA, USA
16 University of Maryland, Department of Geographical Sciences, College Park, MD 20742, USA
17 Environmental Sciences Division and Climate Change Science Institute, Oak Ridge National Laboratory, Oak Ridge, TN, USA
18 School of Life Sciences, Faculty of Science, University of Technology Sydney, Ultimo, NSW, Australia
19 Hawkesbury Institute for the Environment, Western Sydney University, Penrith, NSW, Australia
20 Institute of Applied Energy, Tokyo 105-0003, Japan
21 Georgina Mace Centre for the Living Planet, Department of Life Sciences, Imperial College London, Silwood Park Campus, Buckhurst Road, Ascot, UK
22 Faculty of Environment, Science and Economy, University of Exeter, Exeter, UK
23 Department of Civil and Environmental Engineering, Massachusetts Institute of Technology, Cambridge, MA, USA
24 Center for Earth System Science and Global Sustainability, Schiller Institute for Integrated Science and Society, Boston College, Chestnut Hill, MA, USA
25 Department of Earth and Environmental Sciences, Boston College, Chestnut Hill, MA, USA
26 Laboratoire de Météorologie Dynamique, Institut Pierre-Simon Laplace, CNRS, École Normale Supérieure, Université PSL, Sorbonne Université, École Polytechnique, Paris, France
27 Graduate School of Agricultural and Life Sciences, the University of Tokyo, Tokyo, Japan
28 Climate and Environmental Physics, Physics Institute, University of Bern, Bern, Switzerland
29 Wyss Academy for Nature, University of Bern, Bern, Switzerland

30 Oeschger Center for Climate Change Research, University of Bern, Bern, Switzerland
31 Centre National de Recherches Météorologiques (CNRM), Météo-France, CNRS, Université de Toulouse, Toulouse, France
32 Institute of Geography, University of Bern, Hallerstrasse 12, 3012 Bern, Switzerland
33 Oeschger Centre for Climate Change Research, University of Bern, Falkenplatz 16, 3012 Bern, Switzerland

Table S1 Field evidence on linearity. Please note that subscript is not used to enhance visibility.

Source	Method	Implication	Finding
(Stocker et al., 2024; Van Sundert et al., 2023)	Meta-analysis of eCO2 experiments	LNPP:GPP>1	Vcmax and nitrogen content decrease
(Gielen et al., 2005)	POPFACE	LNPP:GPP>1	NPP:GPP increase
(Leakey et al., 2009)	Synthesis of 15 FACE experiments	LNPP:GPP>1	Vcmax and leaf nitrogen decrease
(Jiang et al., 2020)	EucFACE experiment	LNPP:GPP>1	Leaf respiration decrease; CUE increase.
(Bugmann & Bigler, 2011)	Forest inventory	LCveg*:NPP < 1	Tree growth increase; longevity decrease; decreased vegetation turnover time τ
(Brienen et al., 2015, 2020)	Forest inventory	LCveg*:NPP < 1	Forest mortality increase
(Rogers et al., 1995)	62 eCO2 cropland study sites	LCroot:Cveg > 1	Root allocation increase
(Ainsworth & Long, 2005)	Meta-analysis of FACE experiments	LCroot:Cveg > 1	Belowground allocation increase
(De Kauwe et al., 2014)	Model–data comparisons for two FACE experiments	LCroot:Cveg > 1	Root and rhizosphere allocation increase
(Terrer et al., 2021)	Meta-analysis of eCO2 experiments	LCroot:Cveg > 1; LCveg*:NPP < 1	Carbon allocation shift to root and rhizosphere
(Marqués et al., 2023)	Forest inventory	LCveg*:NPP < 1	Accelerated self-thining
(Walker et al., 2019)	Synthesis of four FACE experiments	LCveg*:NPP > 1	Wood allocation increase
(Walker et al., 2019)	Synthesis of four FACE experiments	LCwood:Cveg > 1	Wood allocation increase
(Stocker et al., 2024; Van Sundert et al., 2023)	Meta-analysis of eCO2 experiments	LCroot:Cveg > 1; LCveg*:NPP < 1	Root:shoot ratio increase
(Walker et al., 2021)	Synthesis of Forest inventory studies	LCveg*:NPP < 1	Vegetation turnover rate increase (given by k_veg in the study)
(Körner, 2017)	Synthesis of Forest inventory studies	LCveg*:NPP < 1	Forest mortality and turnover increase
(Jiang et al., 2020)	EucFACE experiment	LCveg:NPP < 1	Rhizosphere allocation increase. More increase in NPP than biomass for both understorey plants and trees
(Norby & Zak, 2011)	Synthesis of FACE experiments	No concensus for LCleaf:Cveg*	
(Han et al., 2023)	Meta-analysis of eCO2 experiments	LCroot:Cveg > 1	Root:shoot ratio increase
(Franklin, 2007)	Four forest FACE experiments	LCroot:Cveg > 1	Fine-root investment increase
(Phillips et al., 2012)	eCO2 experiments	LCroot:Cveg > 1	Belowground carbon and nitrogen cycling increase
(Fransson, 2012)	eCO2 experiments	LCroot:Cveg > 1	Ectomycorrhizal carbon flow increase
(Gielen et al., 2005)	POPFACE	LCroot:Cveg > 1	Above:belowground NPP decrease
(Norby et al., 2010)	ORNL FACE	LCveg*:NPP < 1; LCroot:Cveg > 1	NPP increase more than wood increment
(Weng et al., 2019)	Model–data comparisons	LCveg*:NPP > 1	Biomass increase more than NPP
Figure S24 (this study) represented from (Walker et al., 2021)	Synthesis of various types of field measurements	LNPP:GPP<1<br>LCveg*:NPP < 1<br>LCroot:Cwood > 1	

Note : EucFACE (Jiang et al., 2020) BiFOR (Norby et al., 2024) and a previous meta-analysis (Curtis & Wang, 1998) did not find change in biomass allocation under eCO2. Walker et al., (2021) wrote in the main text that the eCO2 effect on *C*veg turnover time is uncertain, although some limited data suggest LCveg*:NPP < 1 as shown above. A review of autotrophic respiration reported that the long-term eCO2 effect on autotrophic respiration is really uncertain (Smith, 2017), so although some field evidences are given above, there is no consensus on LCveg*:NPP and LNPP:GPP at the time of writing. Please note that the above is not an exhaustive list. The logical link between 'Finding' and 'Implication' is explained in the main text Discussion.

1. Linearity $L_{NPP:GPP}$

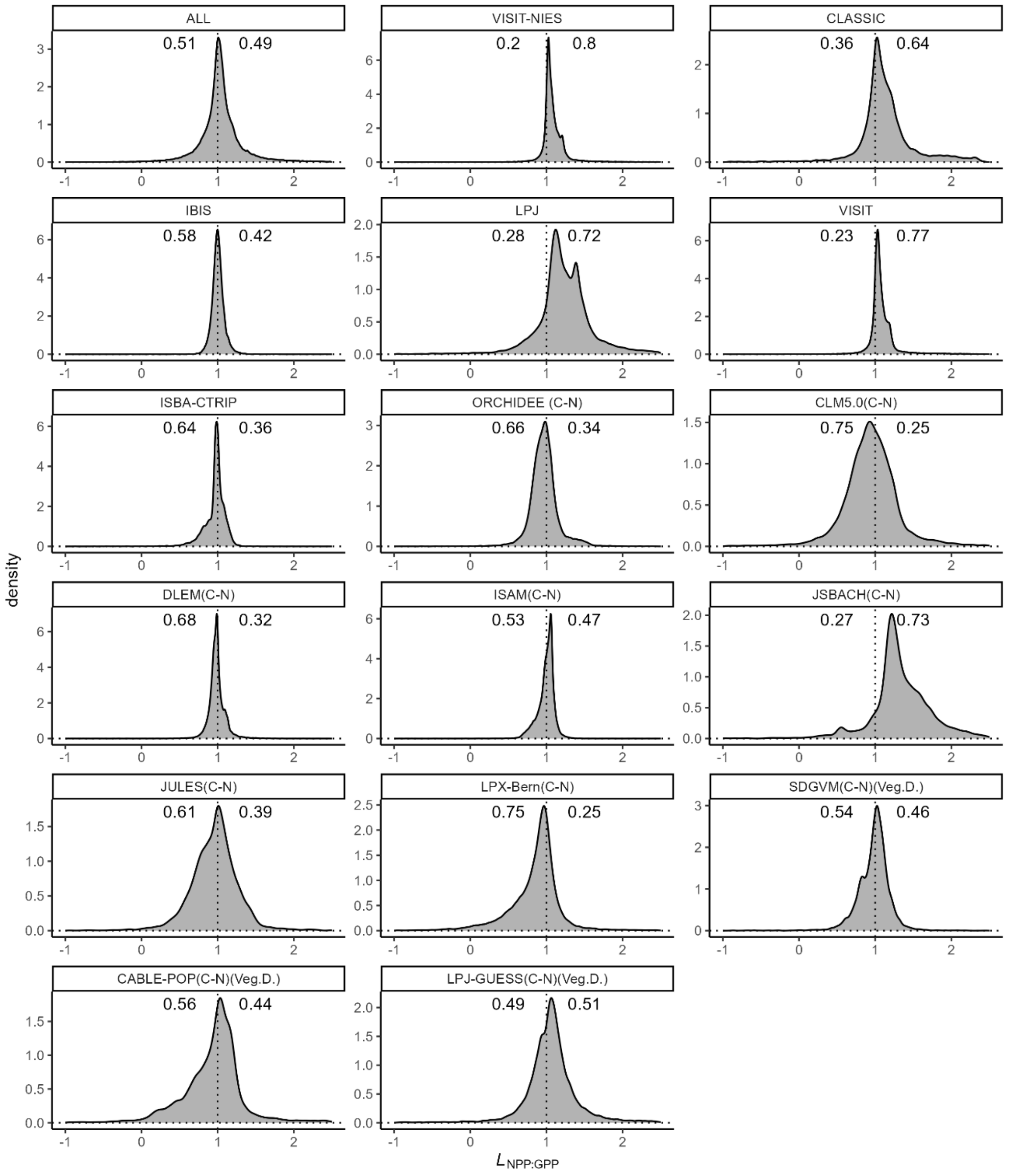

Figure S1 Density of the distribution of $L_{NPP:GPP}$ values across gridcells by model, printed as labels on top of each panel. 'All' shows cross models overall pattern, identical to the figure presented in the main text. The proportion of gridcells with $L_{NPP:GPP} < 1$ ($L_{NPP:GPP} > 1$) is given by the annotation on the left (right) side of the plotting area. The panel labelled 'ALL' represents the joint pattern with data pooled from all models. $L_{NPP:GPP} = R_{NPP} / R_{GPP}$ where R_{NPP} (R_{GPP}) is the relative change of net primary productivity, NPP (gross primary production, GPP), evaluated from simulations with rising CO_2 and changing N deposition. Both L and R terms are unitless. We tagged models as carbon-nitrogen coupled models (C-N) and vegetation demography models (Veg.D.).

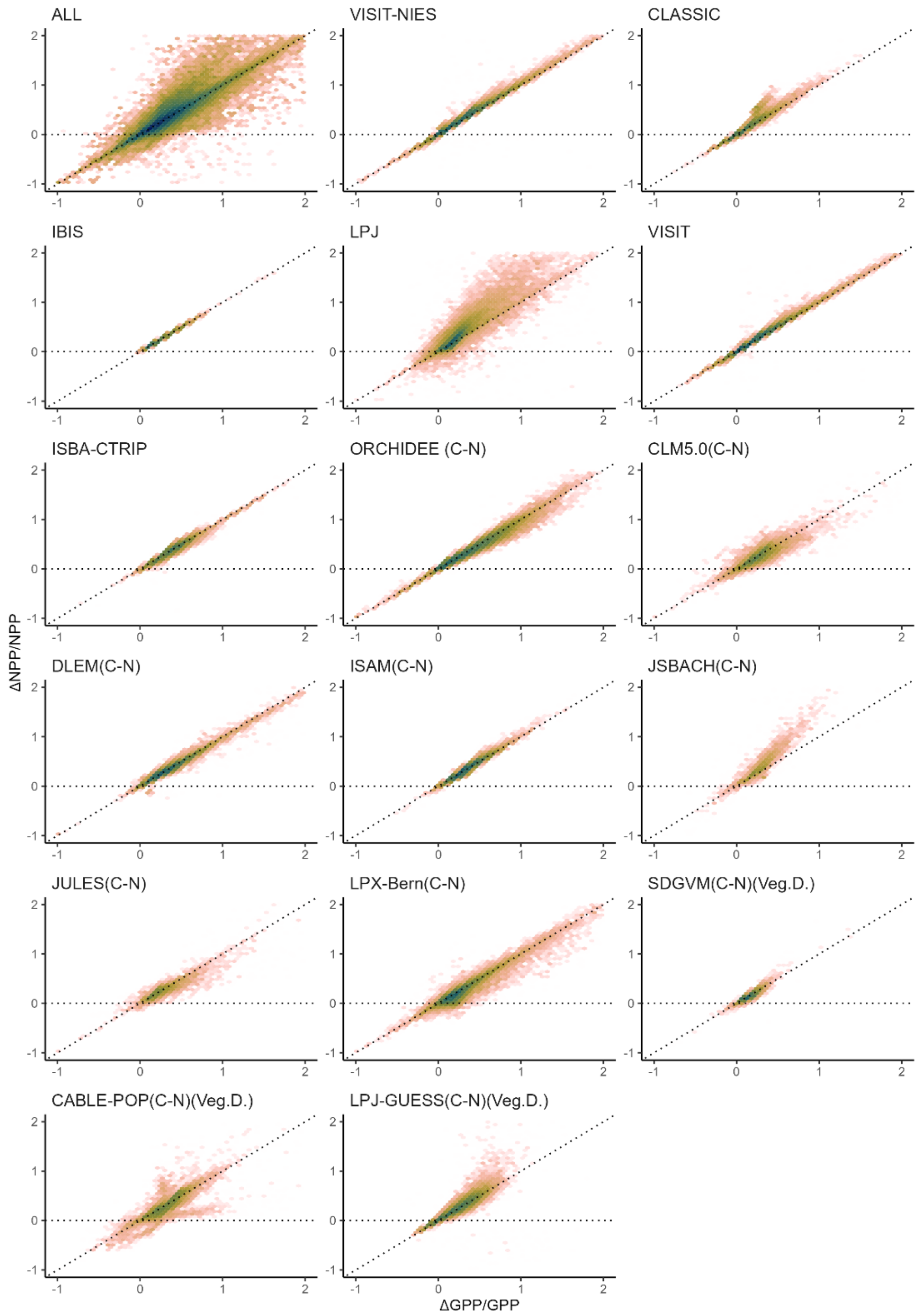

Figure S2 Density scatter plot of R_{NPP} vs. R_{GPP} values across all gridcells for each model. The name of the model is printed on top of each histogram. Dark colors denote high density, while bright colors denote low density. R_{NPP} is the relative change (ΔNPP/NPP, unitless) of net primary productivity (NPP) evaluated from simulations with rising CO_2 and changing N deposition. R_{GPP} is the relative change (ΔGPP/GPP, unitless) of gross primary productivity (GPP), evaluated from the same simulations. The panel labelled 'ALL' represents the joint pattern with data pooled from all models. We tagged models as carbon-nitrogen coupled models (C-N) and vegetation demography models (Veg.D.).

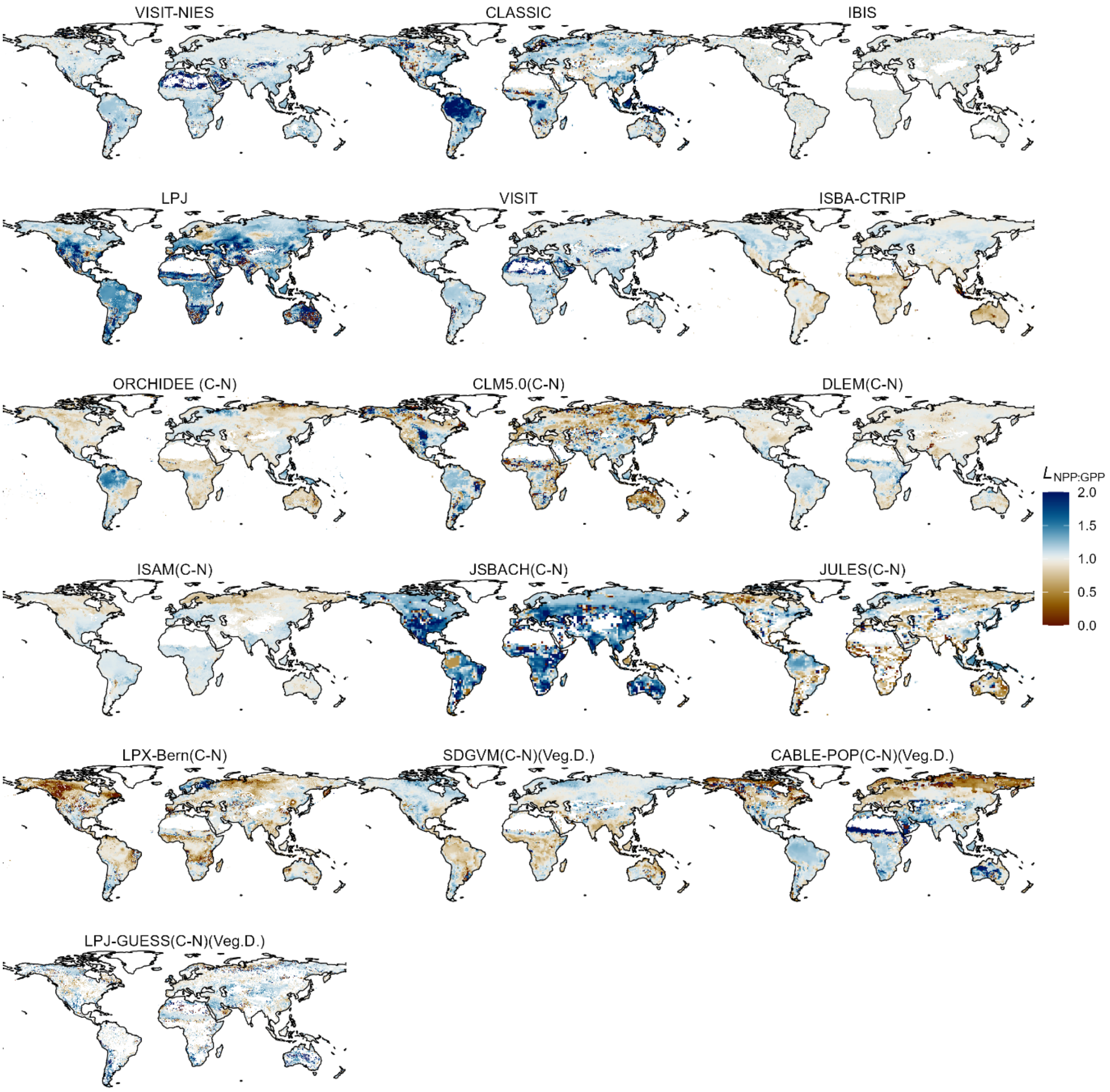

Figure S3 Spatial pattern of $L_{NPP:GPP}$ for each model. The name of each model is printed on top of each map. $L_{NPP:GPP} = R_{NPP} / R_{GPP}$ where R_{NPP} (R_{GPP}) is the relative change of net primary productivity, NPP (gross primary production, GPP), evaluated from simulations with rising CO_2 and changing N deposition. Both L and R terms are unitless. We tagged models as carbon-nitrogen coupled models (C-N) and vegetation demography models (Veg.D.).

2. Linearity $L_{Cveg*:NPP}$

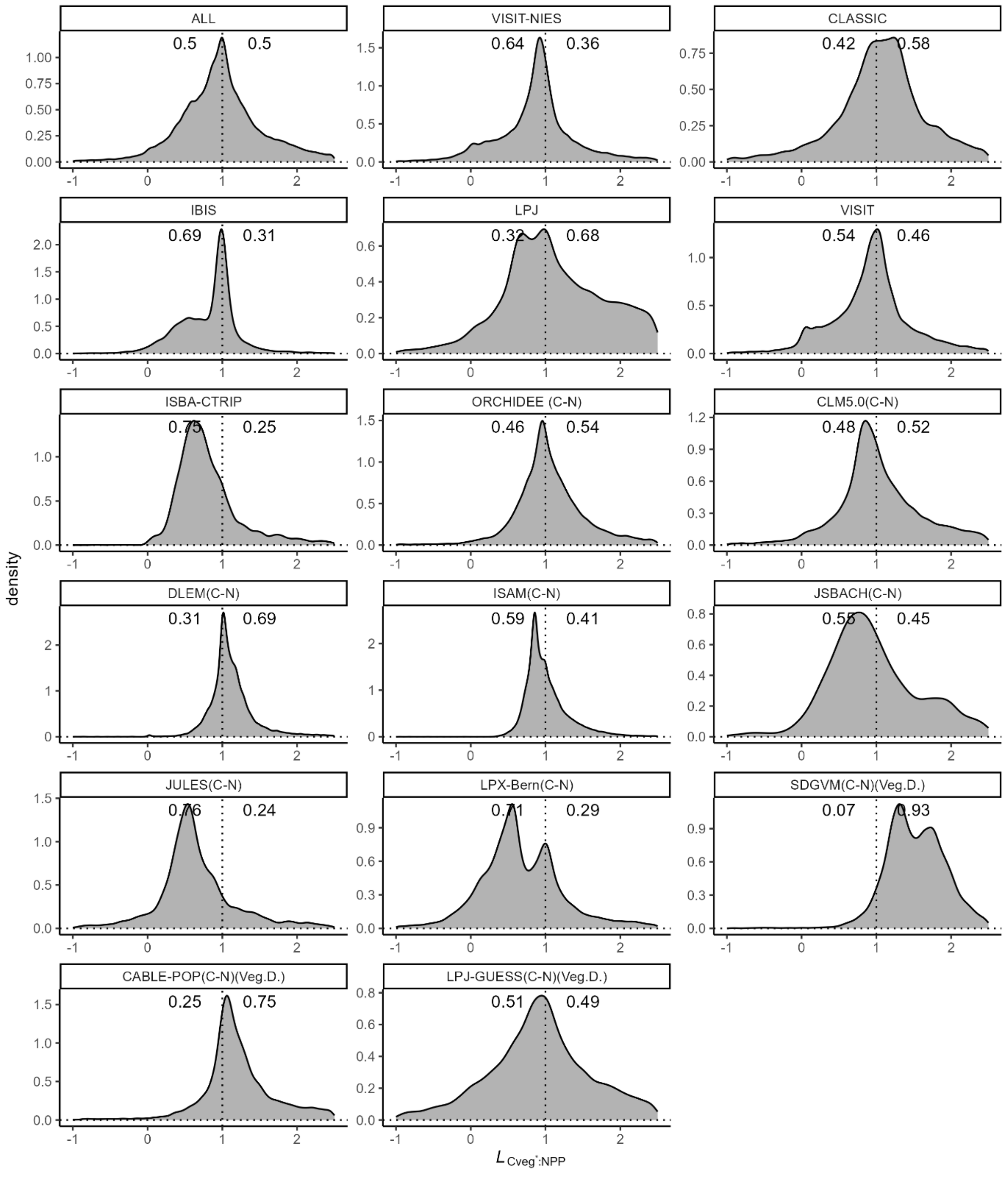

Figure S4 Density of the distribution of $L_{Cveg*:NPP}$ values across gridcells by model, printed as labels on top of each panel. The proportion of gridcells with $L_{Cveg*:NPP} < 1$ ($L_{Cveg*:NPP} > 1$) is given by the annotation on the left (right) side of the plotting area. The panel labelled 'ALL' represents the joint pattern with data pooled from all models. $L_{\mathrm{Cveg*:NPP}} = R_{\mathrm{Cveg*}} / R_{\mathrm{NPP}}$ where R_{NPP} (R_{Cveg}) is the relative change of net primary productivity, NPP (steady-state total vegetation C pools), evaluated from simulations with rising CO_2 and changing N deposition. Both L and R terms are unitless. We tagged models as carbon-nitrogen coupled models (C-N) and vegetation demography models (Veg.D.).

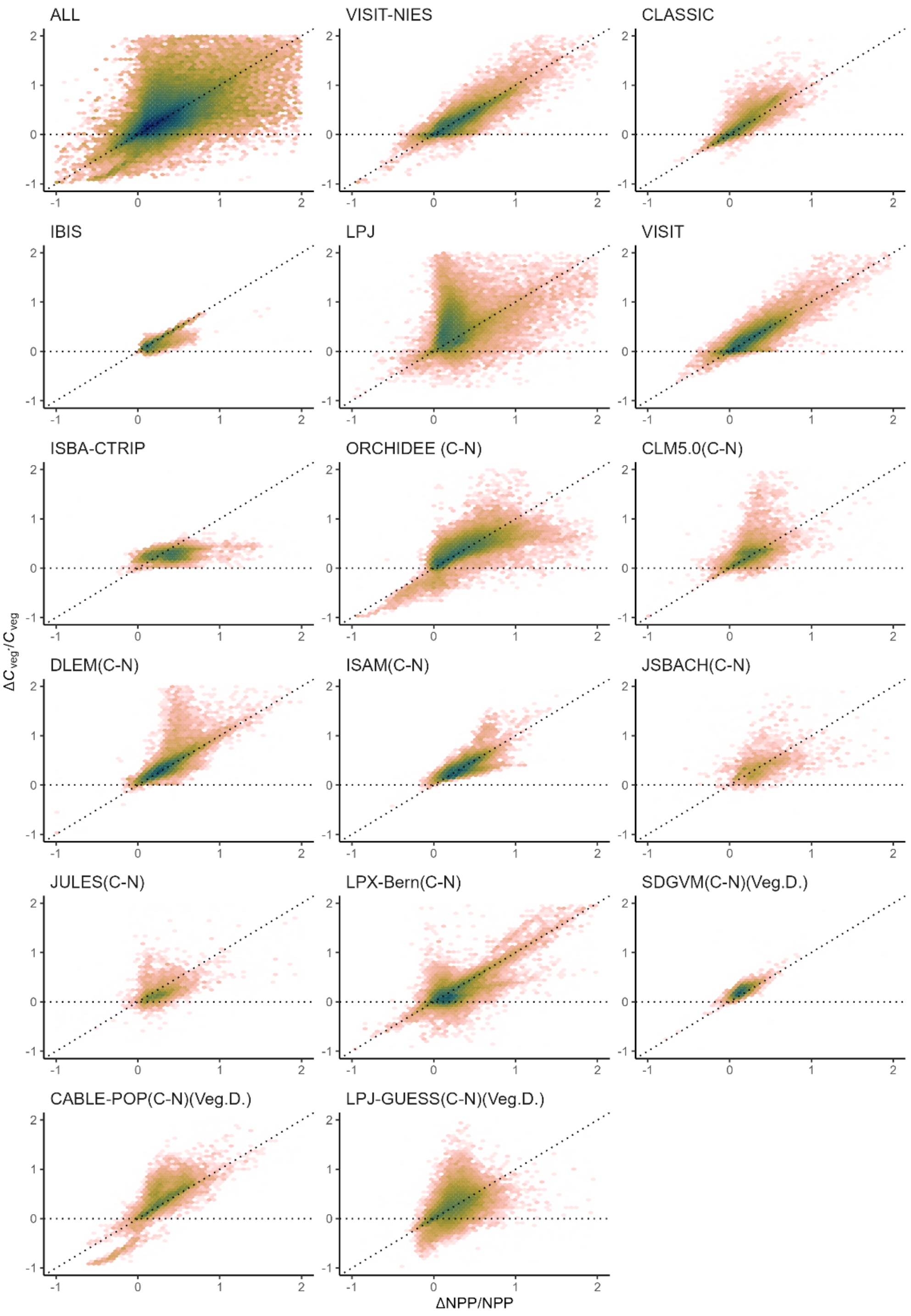

Figure S5 Density scatter plot of R_{NPP} vs. R_{Cveg*} values across all gridcells for each model. The name of the model is printed on top of each histogram. Dark colors denote high density, while bright colors denote low density. R_{NPP} is the relative change (ΔNPP/NPP, unitless) of net primary productivity (NPP) evaluated from simulations with rising CO_2 and changing N deposition. R_{Cveg*} is the relative change ($\Delta C_{veg*}/C_{veg*}$, unitless) of total vegetation carbon, evaluated from the same simulations. The panel labelled 'ALL' represents the joint pattern with data pooled from all models. We tagged models as carbon-nitrogen coupled models (C-N) and vegetation demography models (Veg.D.).

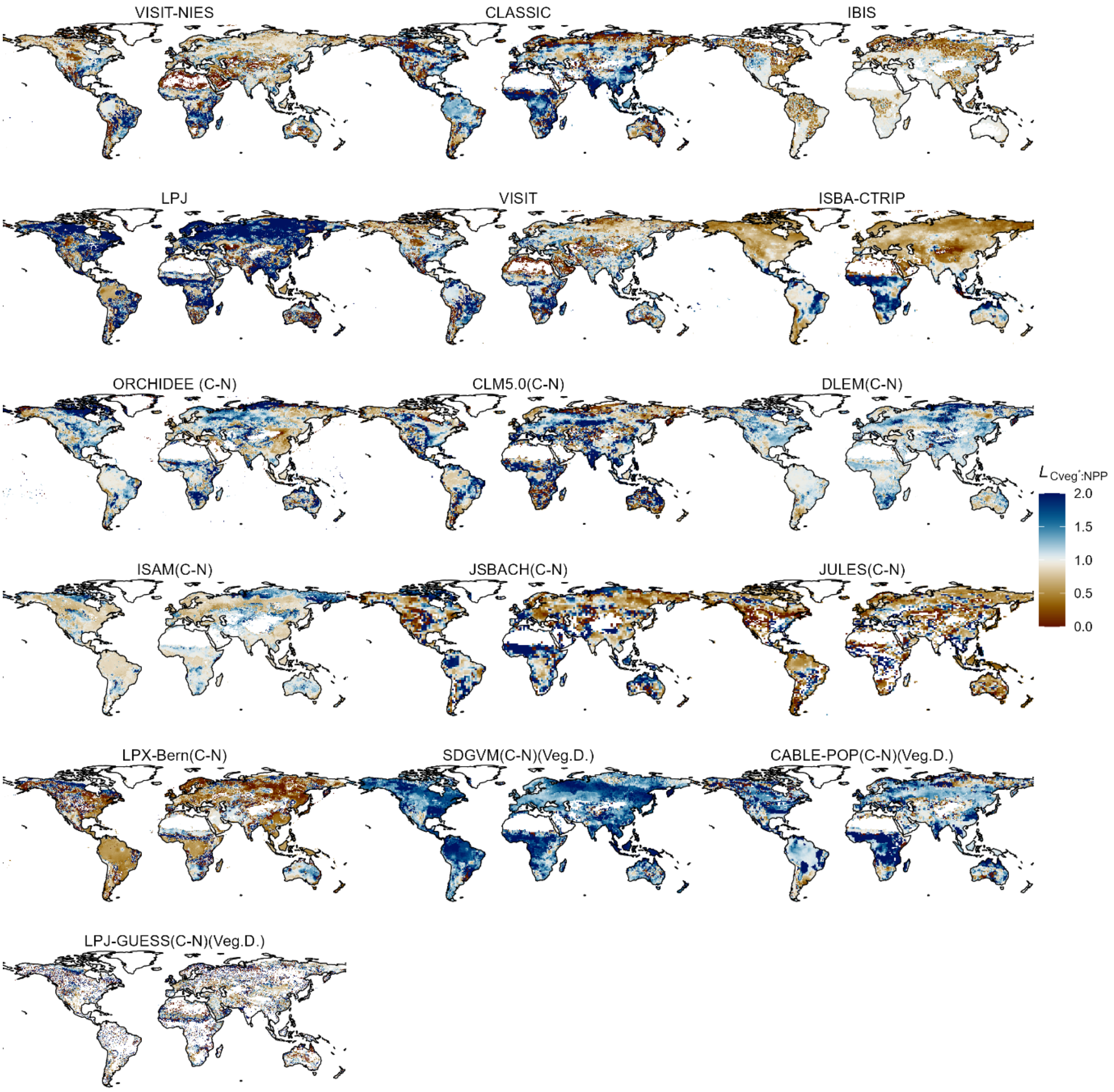

Figure S6 Spatial pattern of $L_{Cveg*:NPP}$ for each model. The name of each model is printed on top of each map. $L_{Cveg*:NPP} = R_{Cveg*} / R_{NPP}$ where R_{Cveg*} is the relative change of the steady-state estimate of total vegetation carbon, and R_{NPP} is the relative change in net primary production (NPP), evaluated from simulations with rising CO_2 and changing N deposition. Both L and R terms are unitless.

3. Linearity $L_{\mathrm{Croot:Cveg}}$

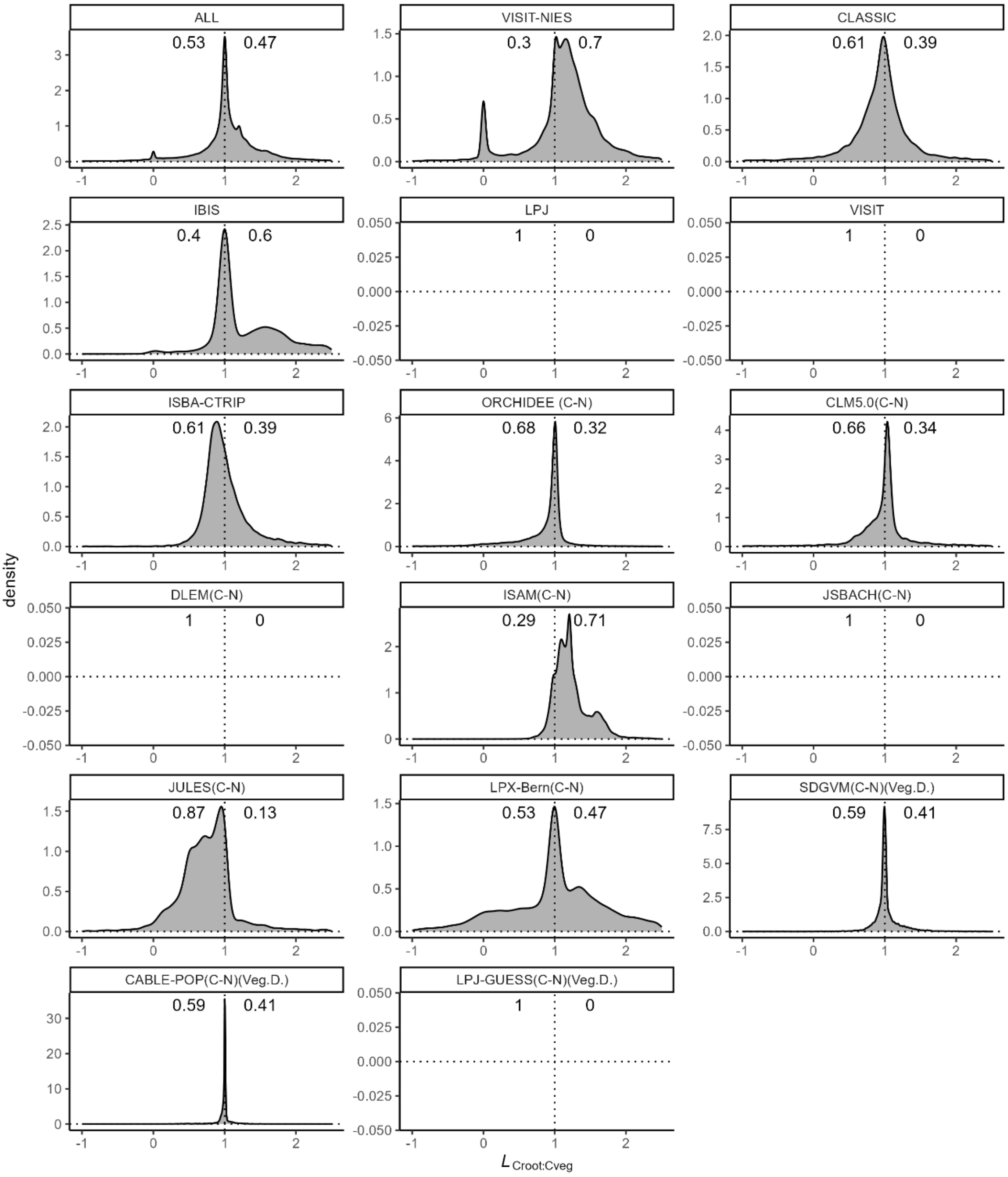

Figure S7 Density of the distribution of $L_{\mathrm{Croot:Cveg}}$ values across gridcells by model, printed as labels on top of each panel. The proportion of gridcells with $L_{\mathrm{Croot:Cveg}} < 1$ ($L_{\mathrm{Croot:Cveg}} > 1$) is given by the annotation on the left (right) side of the plotting area. The panel labelled 'ALL' represents the joint pattern with data pooled from all models. $L_{\mathrm{Croot:Cveg}} = R_{\mathrm{Croot}} / R_{\mathrm{Cveg}}$ where R_{Croot} (R_{Cveg}) are the relative change of root C (total vegetation C), evaluated from simulations with rising CO_2 and changing N deposition. Both L and R terms are unitless. We tagged models as carbon-nitrogen coupled models (C-N) and vegetation demography models (Veg.D.).

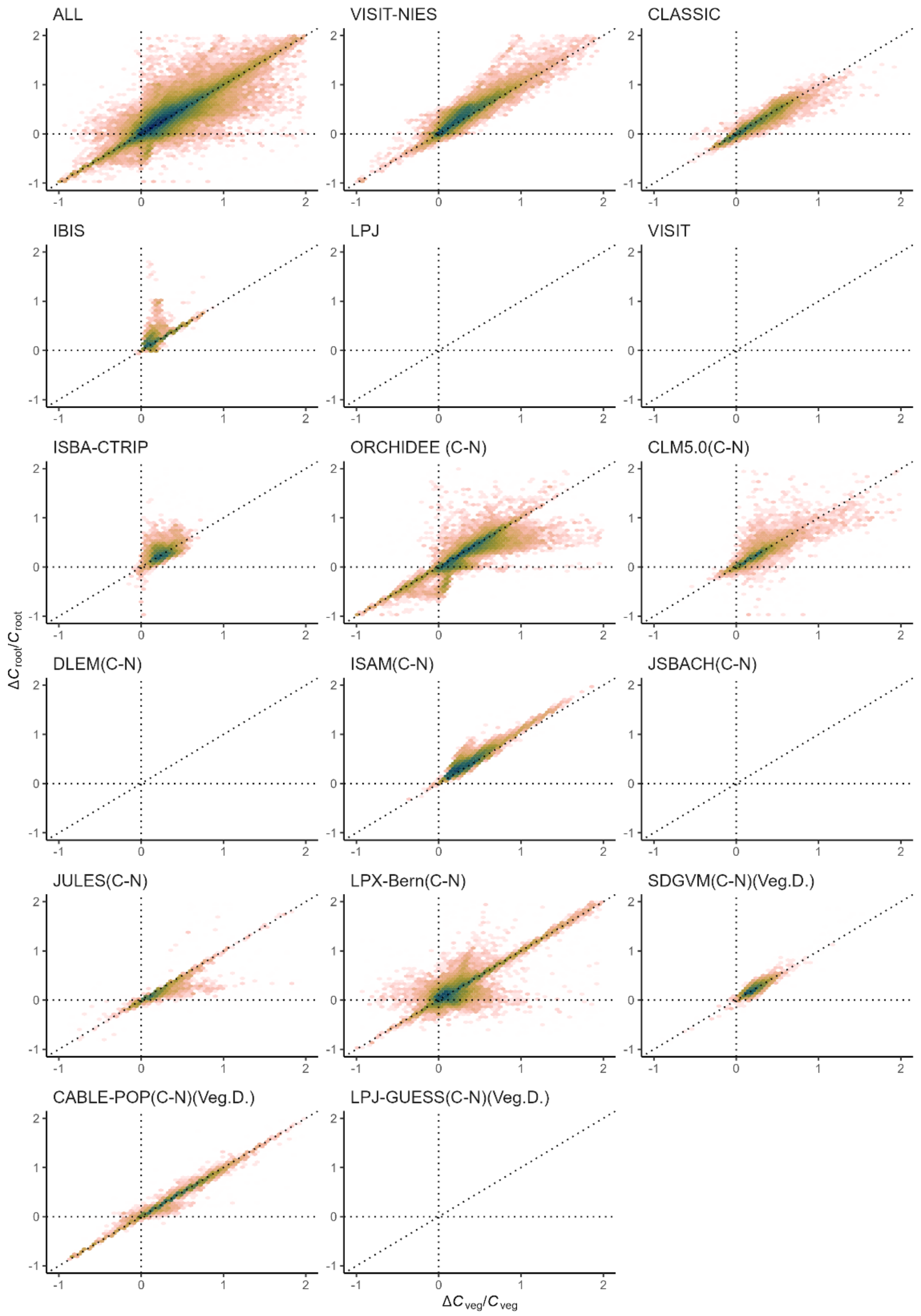

Figure S8 Density scatter plot of R_{Croot} vs. R_{Cveg} values across all gridcells for each model. The name of the model is printed on top of each histogram. Dark colors denote high density, while bright colors denote low density. R_{Croot} is the relative change ($\Delta C_{root}/C_{root}$, unitless) of root C. R_{Cveg} is the relative change ($\Delta C_{veg}/C_{veg}$, unitless) of total vegetation C. Both relative changes are evaluated from the same simulations. The panel labelled 'ALL' represents the joint pattern with data pooled from all models. We tagged models as carbon-nitrogen coupled models (C-N) and vegetation demography models (Veg.D.).

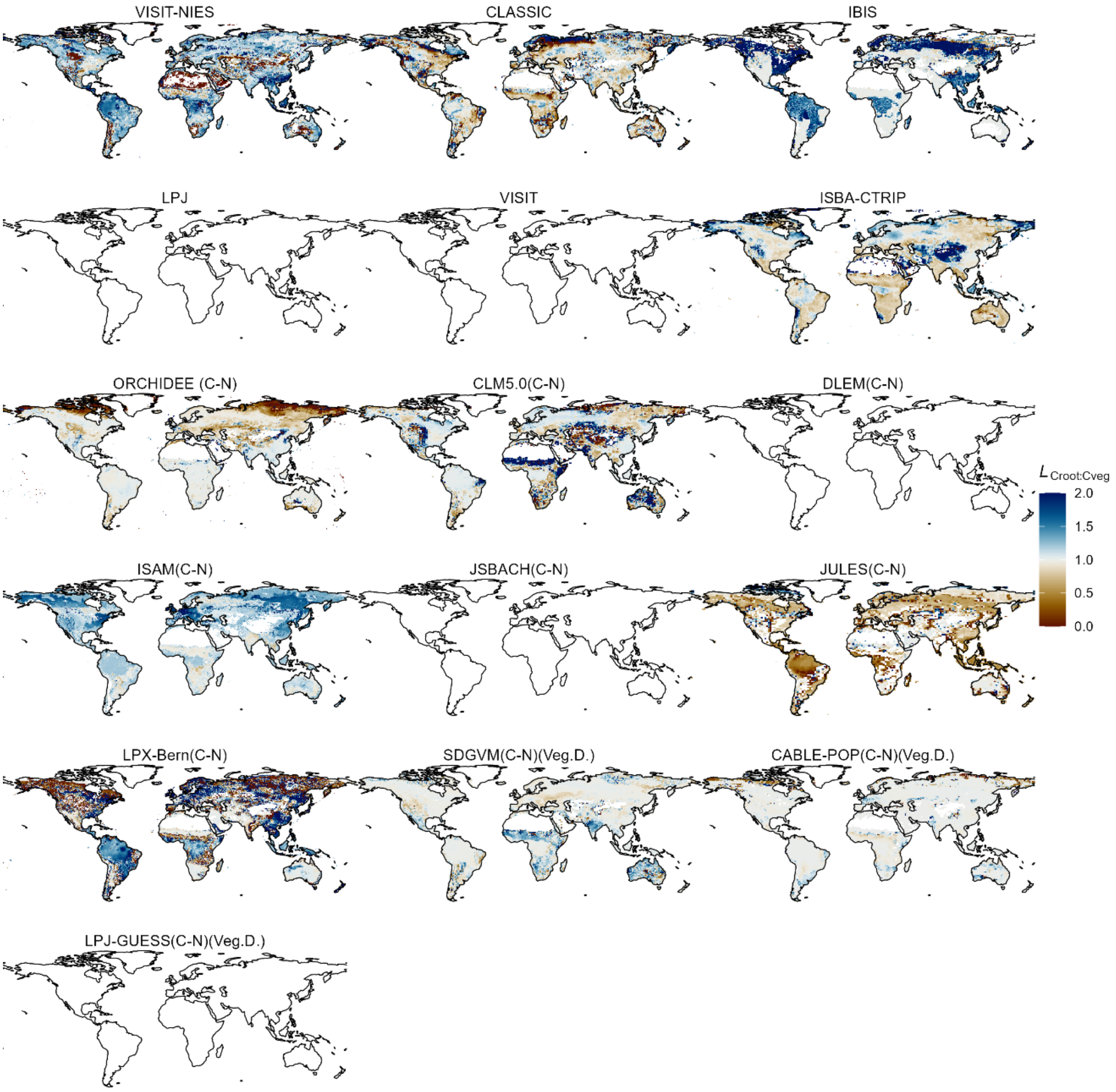

Figure S9 Spatial pattern of $L_{Croot:Cveg}$ for each model. The name of each model is printed on top of each map. $L_{Croot:Cveg} = R_{Croot} / R_{Cveg}$ where R_{Croot} (R_{Cveg}) is the relative change in root C (total vegetation C), evaluated from simulations with rising CO_2 and changing N deposition. Both L and R terms are unitless. We tagged models as carbon-nitrogen coupled models (C-N) and vegetation demography models (Veg.D.).

4. Linearity $L_{Croot:Cveg*}$

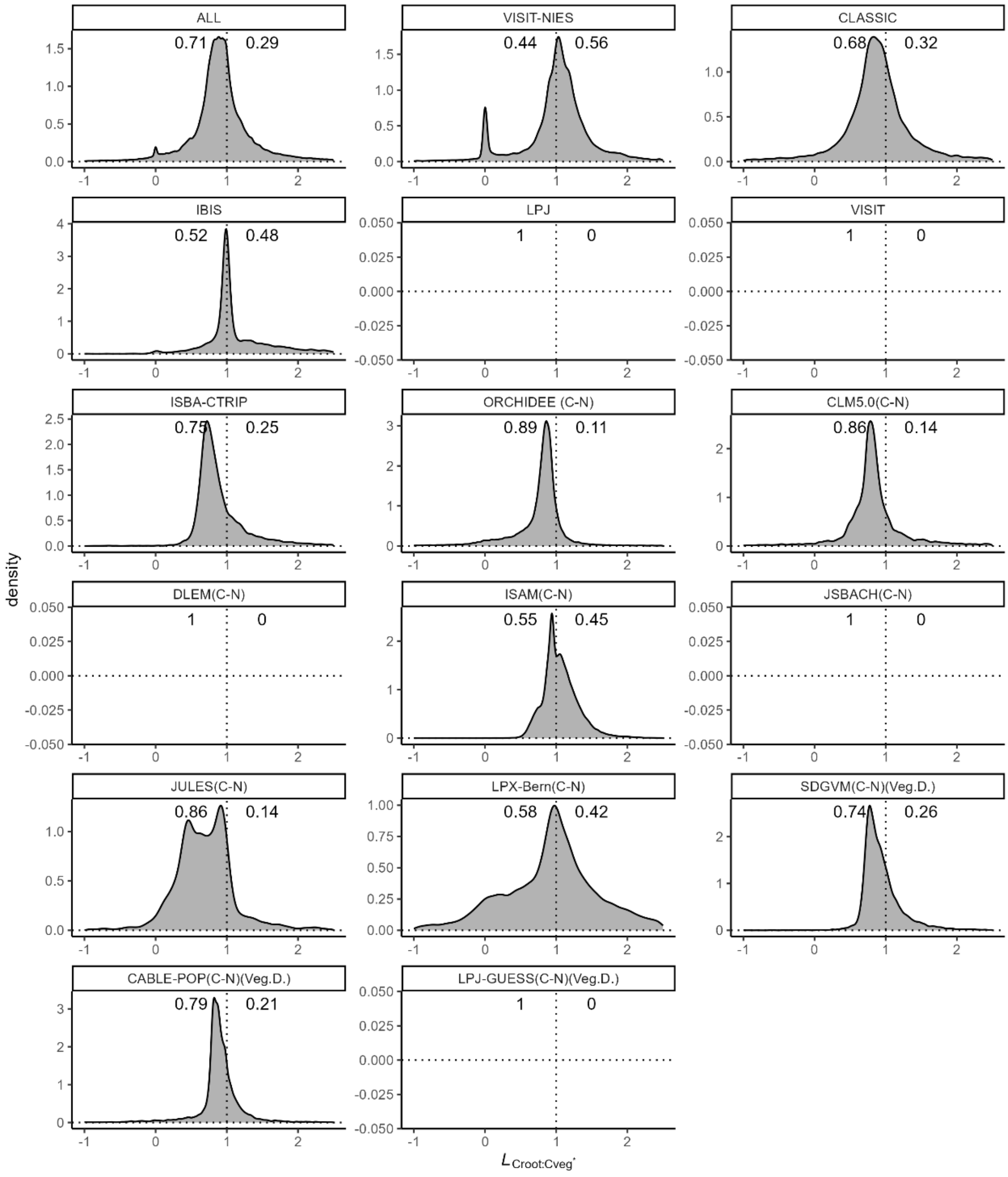

Figure S10 Density of the distribution of $L_{Croot:Cveg*}$ values across gridcells by model, printed as labels on top of each panel. The proportion of gridcells with $L_{Croot:Cveg*} < 1$ ($L_{Croot:Cveg*} > 1$) is given by the annotation on the left (right) side of the plotting area. The panel labelled 'ALL' represents the joint pattern with data pooled from all models. $L_{Croot:Cveg*} = R_{Croot} / R_{Cveg*}$ where R_{Croot} are the relative change of root C (steady-state total vegetation C), evaluated from simulations with rising CO_2 and changing N deposition. Both L and R terms are unitless. As many models include coarse roots in C_{root}, C_{root} should not have rapid response to carbon fertilization. Ideally, we should apply steady state conversion to C_{root}, and calculate $L_{Croot*:Cveg*}$ but this could not be implemented due to the lack of C_{root} turnover time. We believe that $L_{Croot:Cveg*}$ is less useful than $L_{Croot:Cveg}$.

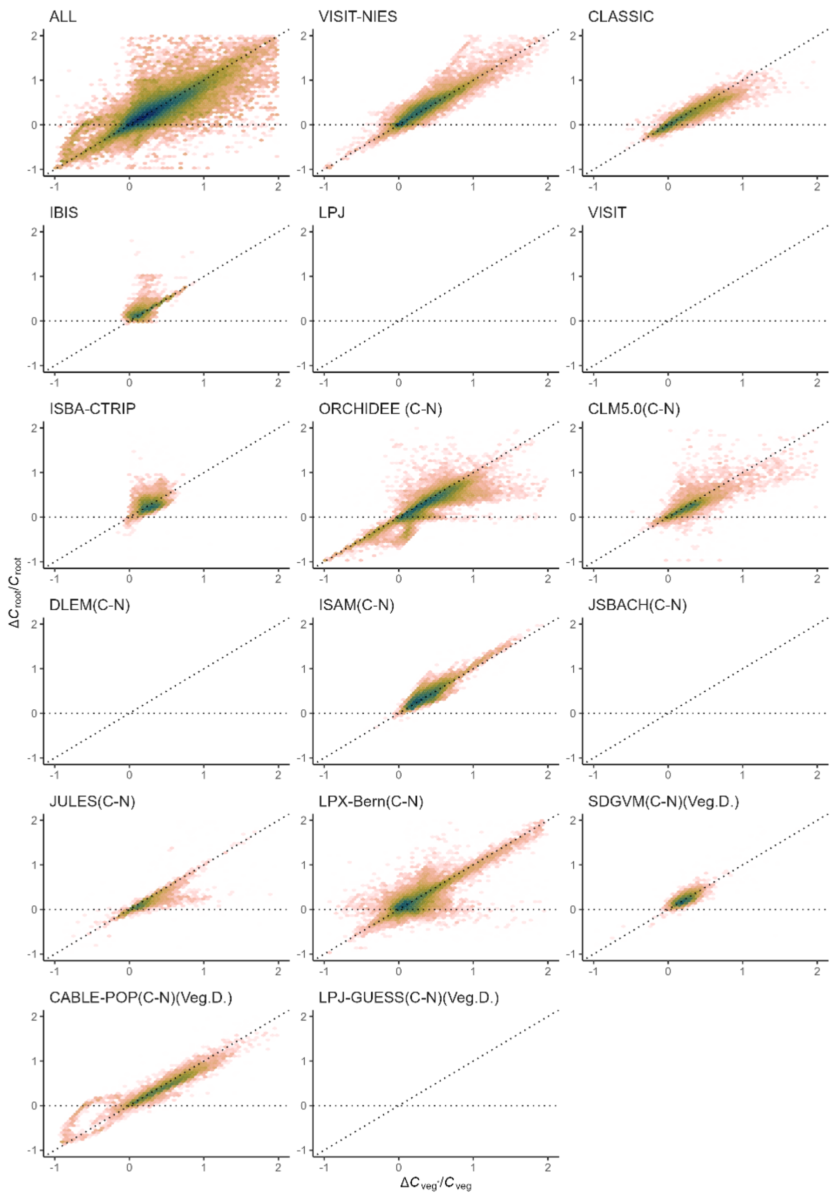

Figure S11 Density scatter plot of R_{Croot} vs. $R_{\mathrm{Cveg*}}$ values across all gridcells for each model. The name of the model is printed on top of each histogram. Dark colors denote high density, while bright colors denote low density. R_{Croot} is the relative change ($\Delta C_{root}/C_{root}$, unitless) of root C. $R_{\mathrm{Cveg*}}$ is the relative change ($\Delta C_{veg*}/C_{veg}$, unitless) of steady-state total vegetation C. Both relative changes are evaluated from the same simulations. The panel labelled 'ALL' represents the joint pattern with data pooled from all models. As many models include coarse roots in C_{root}, C_{root} should not have rapid response to carbon fertilization. Ideally, we should apply steady state conversion to C_{root}, and calculate $L_{\mathrm{Croot*:Cveg*}}$ but this could not be implemented due to the lack of C_{root} turnover time. We believe that $L_{\mathrm{Croot:Cveg*}}$ is less useful than $L_{\mathrm{Croot:Cveg}}$ (Figure S8).

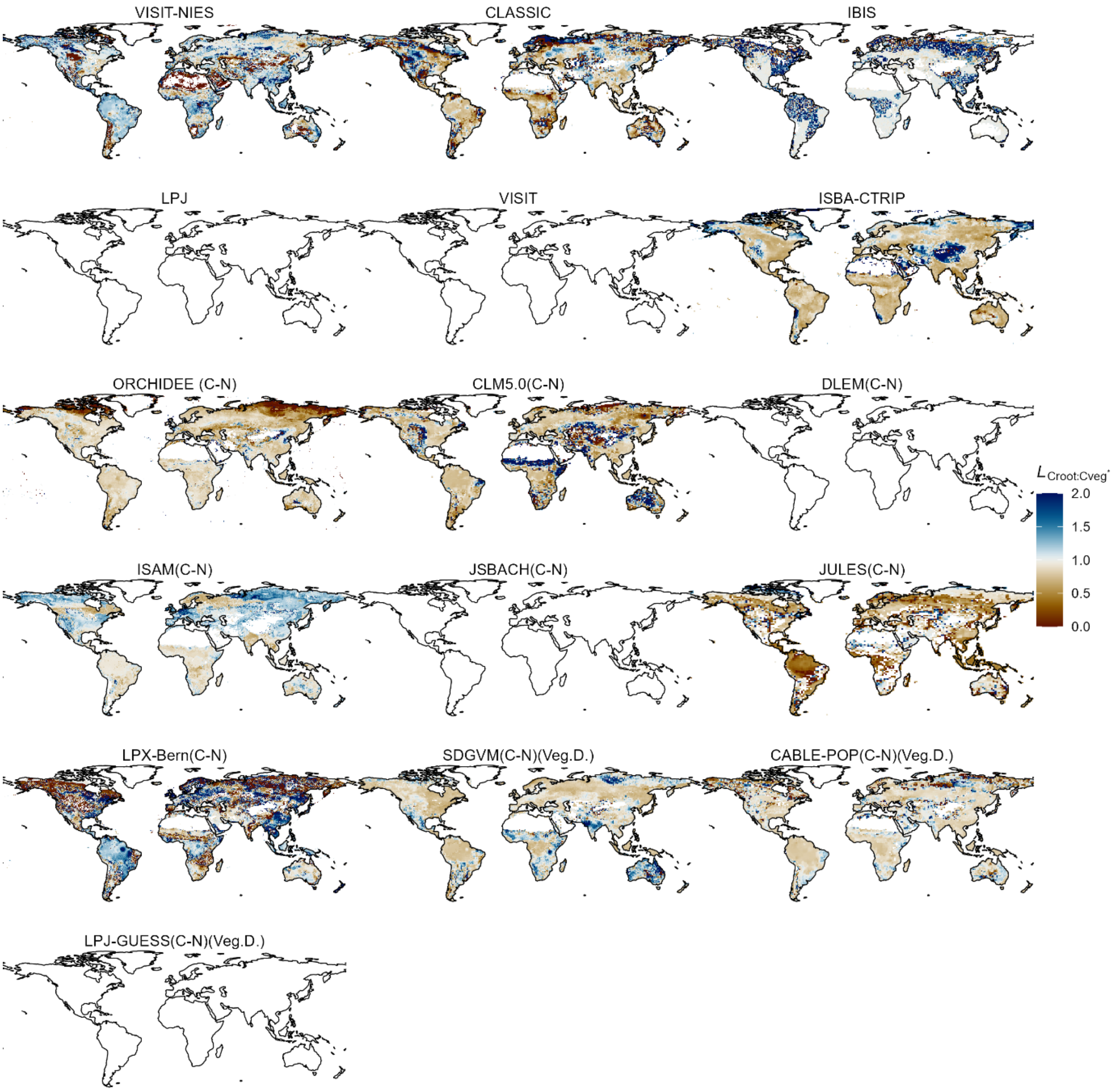

Figure S12 Spatial pattern of $L_{\mathrm{Croot:Cveg*}}$ for each model. The name of each model is printed on top of each map. $L_{\mathrm{Croot:Cveg*}} = R_{\mathrm{Croot}} / R_{\mathrm{Cveg*}}$ where R_{Croot} ($R_{\mathrm{Cveg*}}$) is the relative change in root C (steady-state total vegetation C), evaluated from simulations with rising CO_2 and changing N deposition. Both L and R terms are unitless. As many models include coarse roots in C_{root}, C_{root} should not have rapid response to carbon fertilization. Ideally, we should apply steady state conversion to C_{root}, and calculate $L_{\mathrm{Croot*:Cveg*}}$ but this could not be implemented due to the lack of C_{root} turnover time. We believe that $L_{\mathrm{Croot:Cveg*}}$ is less useful than $L_{\mathrm{Croot:Cveg}}$ (Figure S8).

5. Linearity $L_{Cleaf:Cveg*}$

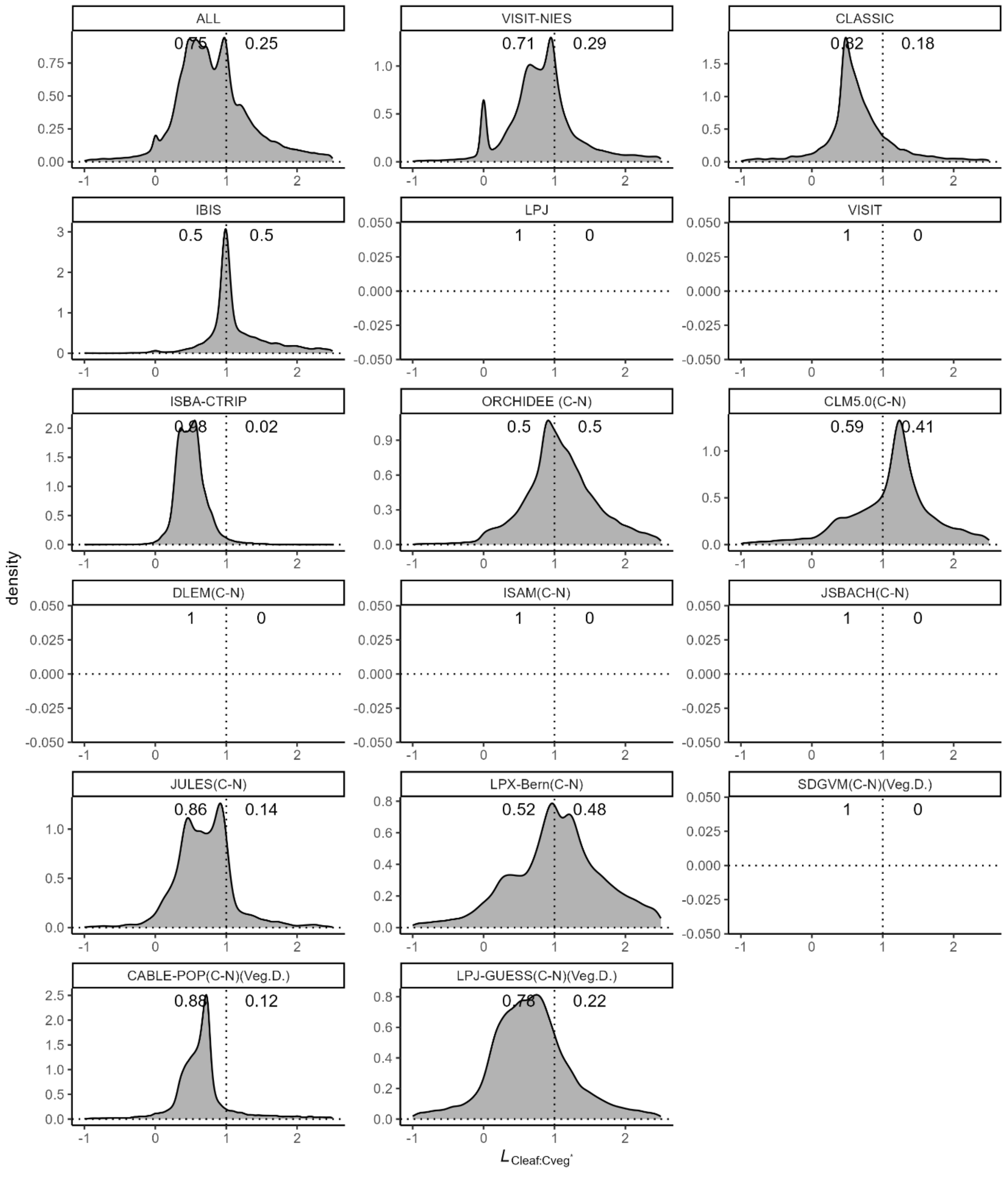

Figure S13 Density of the distribution of $L_{Cleaf:Cveg*}$ values across gridcells by model, printed as labels on top of each panel. The proportion of gridcells with $L_{Cleaf:Cveg*} < 1$ ($L_{Cleaf:Cveg*} > 1$) is given by the annotation on the left (right) side of the plotting area. The panel labelled 'ALL' represents the joint pattern with data pooled from all models. $L_{Cleaf:Cveg*} = R_{Cleaf} / R_{Cveg*}$ where R_{Cleaf} are the relative change of leaf C (steady-state total vegetation C), evaluated from simulations with rising CO_2 and changing N deposition. We tagged models as carbon-nitrogen coupled models (C-N) and vegetation demography models (Veg.D.).

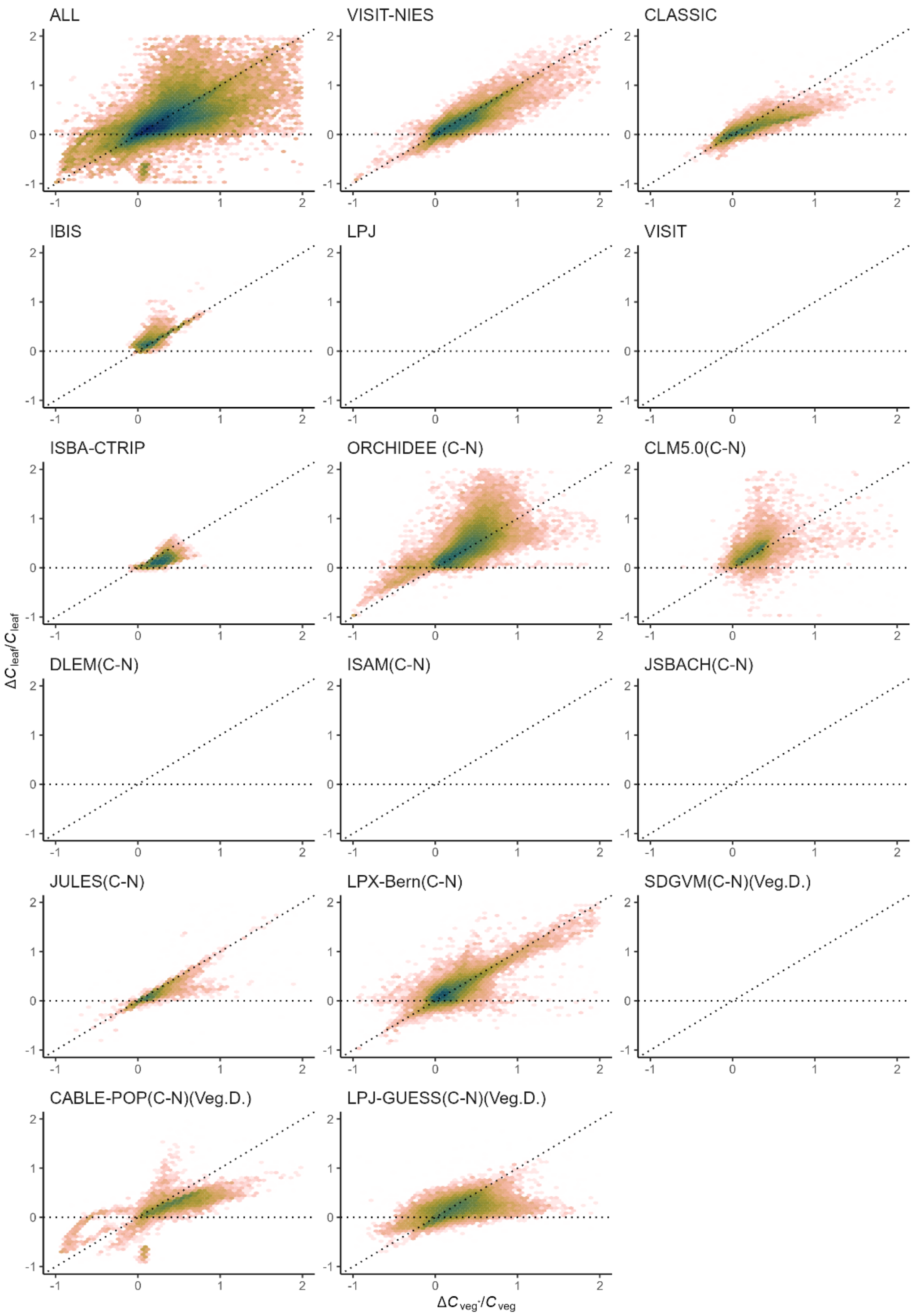

Figure S14 Density scatter plot of R_{Cleaf} vs. $R_{\mathrm{Cveg*}}$ values across all gridcells for each model. The name of the model is printed on top of each histogram. Dark colors denote high density, while bright colors denote low density. R_{Cleaf} is the relative change ($\Delta C_{leaf}/C_{leaf}$, unitless) of leaf C. $R_{\mathrm{Cveg*}}$ is the relative change ($\Delta C_{veg*}/C_{veg}$, unitless) of steady-state total vegetation C. Both relative changes are evaluated from the same simulations. The panel labelled ‘ALL’ represents the joint pattern with data pooled from all models. We tagged models as carbon-nitrogen coupled models (C-N) and vegetation demography models (Veg.D.).

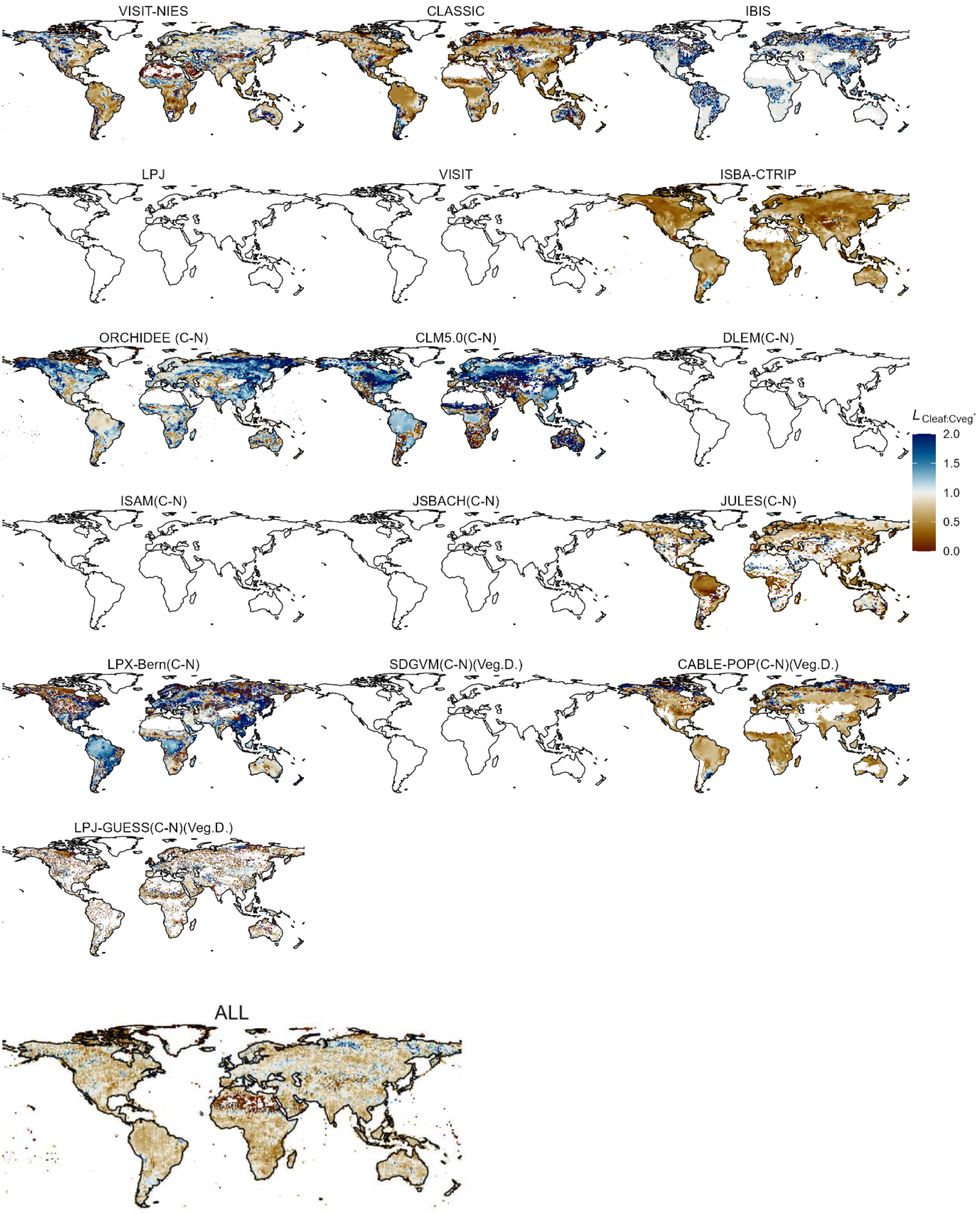

Figure S15 Spatial pattern of $L_{\mathrm{Cleaf:Cveg*}}$ for each model. The name of each model is printed on top of each map. $L_{\mathrm{Cleaf:Cveg*}} = R_{\mathrm{Cleaf}} / R_{\mathrm{Cveg*}}$ where R_{Cleaf} ($R_{\mathrm{Cveg*}}$) is the relative change in leaf C (steady-state total vegetation C), evaluated from simulations with rising CO_2 and changing N deposition. Both L and R terms are unitless. We tagged models as carbon-nitrogen coupled models (C-N) and vegetation demography models (Veg.D.).

6. Linearity $L_{Cwood:Cveg}$

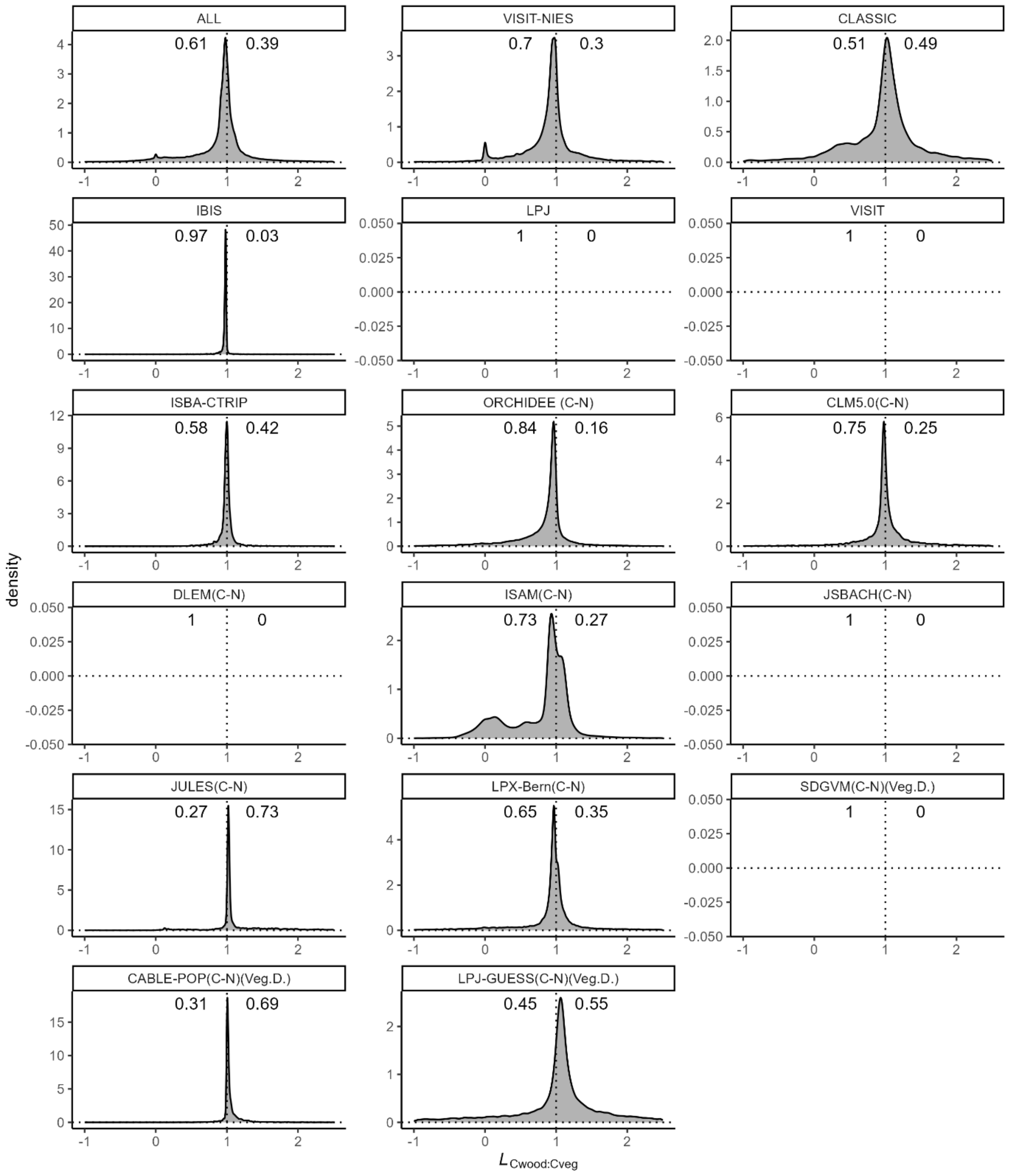

Figure S16 Density of the distribution of $L_{Cwood:Cveg}$ values across gridcells by model, printed as labels on top of each panel. The proportion of gridcells with $L_{Cwood:Cveg} < 1$ ($L_{Cwood:Cveg} > 1$) is given by the annotation on the left (right) side of the plotting area. The panel labelled 'ALL' represents the joint pattern with data pooled from all models. $L_{Cwood:Cveg} = R_{Cwood} / R_{Cveg}$ where R_{Cwood} (R_{Cveg}) are the relative change of wood C (total vegetation C), evaluated from simulations with rising CO_2 and changing N deposition. Both L and R terms are unitless. We tagged models as carbon-nitrogen coupled models (C-N) and vegetation demography models (Veg.D.).

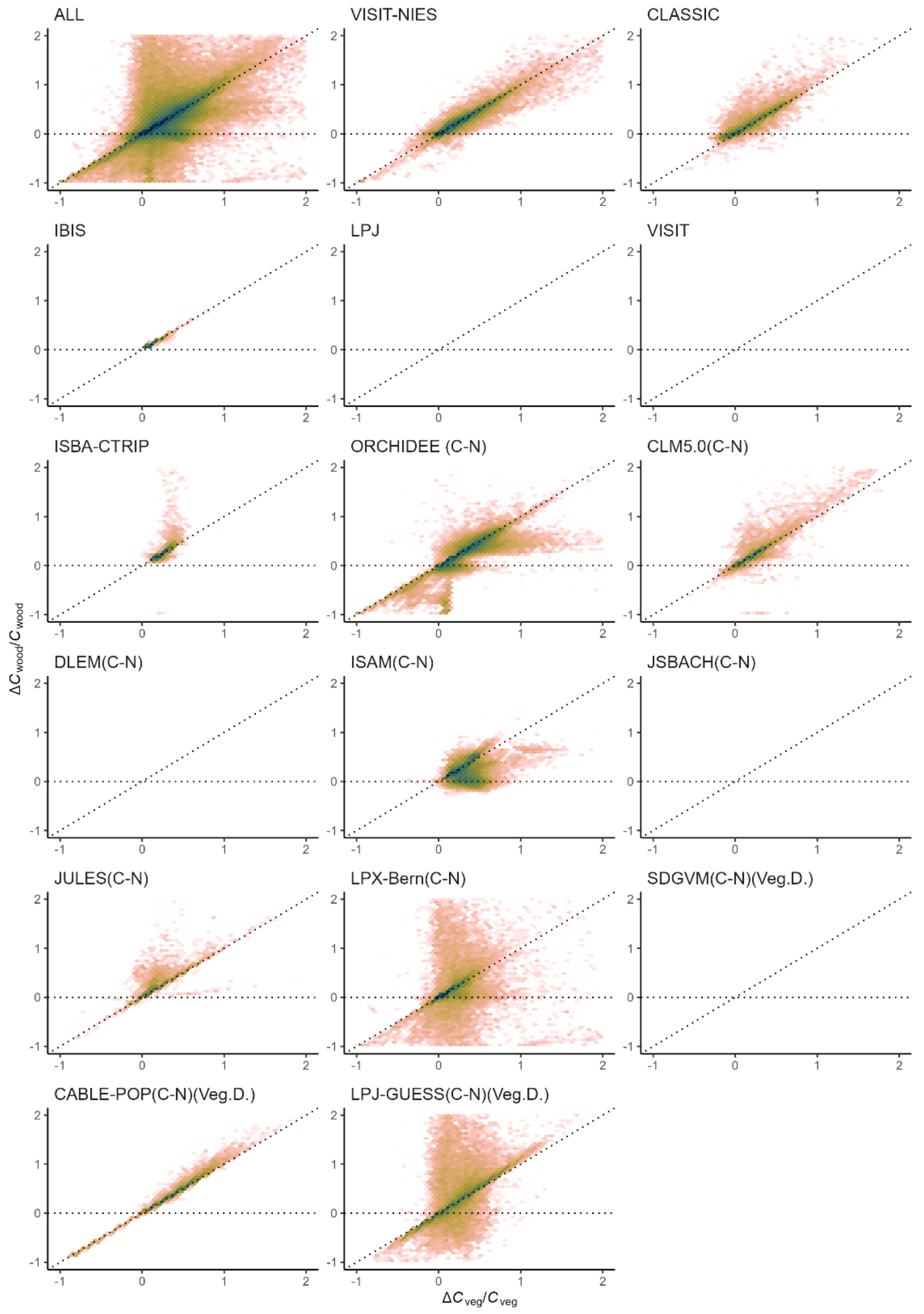

Figure S17 Density scatter plot of R_{Cwood} vs. R_{Cveg} values across all gridcells for each model. The name of the model is printed on top of each histogram. Dark colors denote high density, while bright colors denote low density. R_{Cwood} is the relative change ($\Delta C_{\text{wood}}/C_{\text{wood}}$, unitless) of wood C. R_{Cveg} is the relative change ($\Delta C_{\text{veg}}/C_{\text{veg}}$, unitless) of total vegetation C. Both relative changes are evaluated from the same simulations. The panel labelled 'ALL' represents the joint pattern with data pooled from all models. We tagged models as carbon-nitrogen coupled models (C-N) and vegetation demography models (Veg.D.).

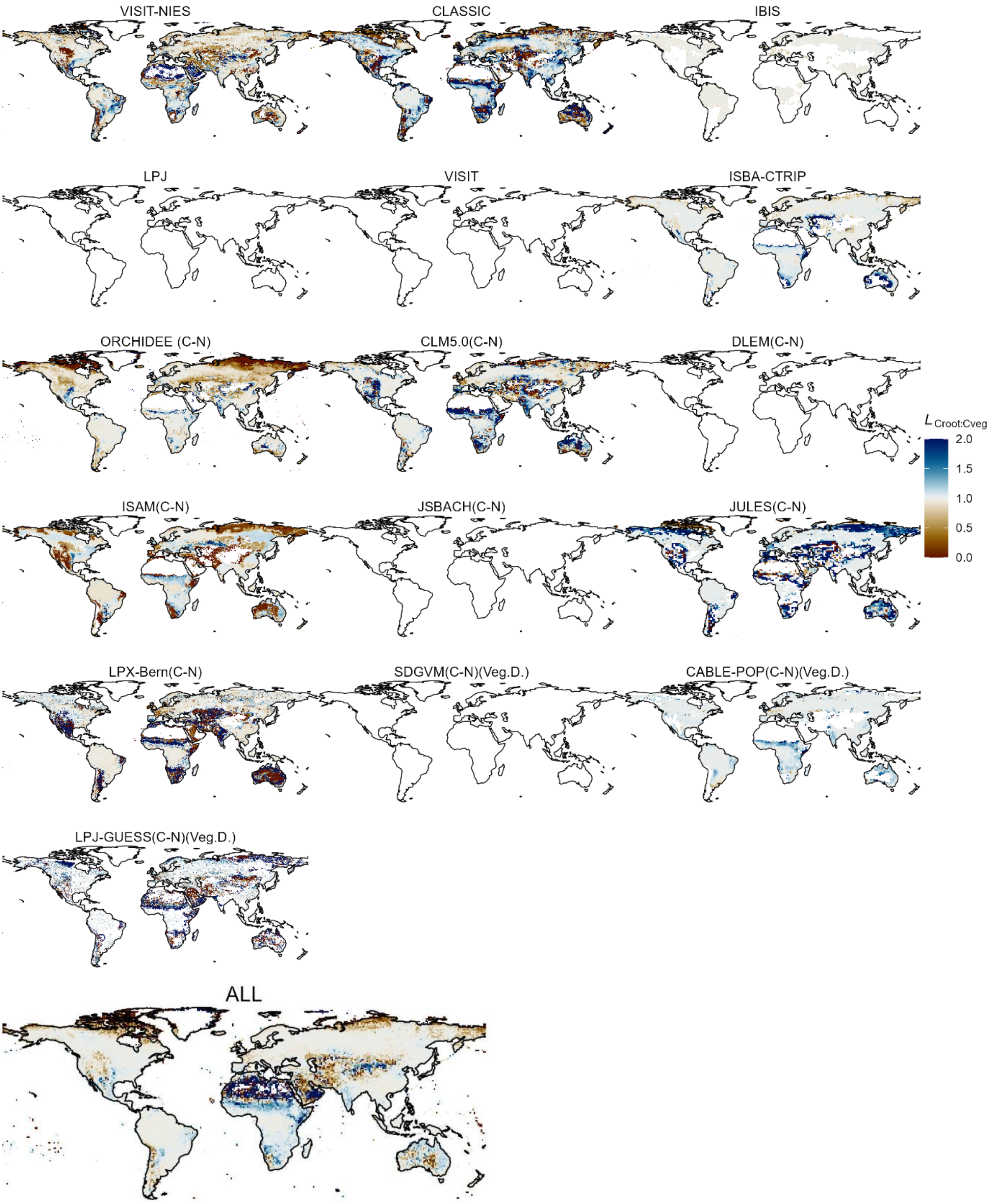

Figure S18 Spatial pattern of $L_{\mathrm{Cwood:Cveg}}$ for each model. The name of each model is printed on top of each map. $L_{\mathrm{Cwood:Cveg}} = R_{\mathrm{Cwood}} / R_{\mathrm{Cve*}}$ where R_{Cwood} (R_{Cveg}) is the relative change in wood C (steady-state total vegetation C), evaluated from simulations with rising CO_2 and changing N deposition. Both L and R terms are unitless. We tagged models as carbon-nitrogen coupled models (C-N) and vegetation demography models (Veg.D.).

7. Linearity $L_{Cveg:NPP}$

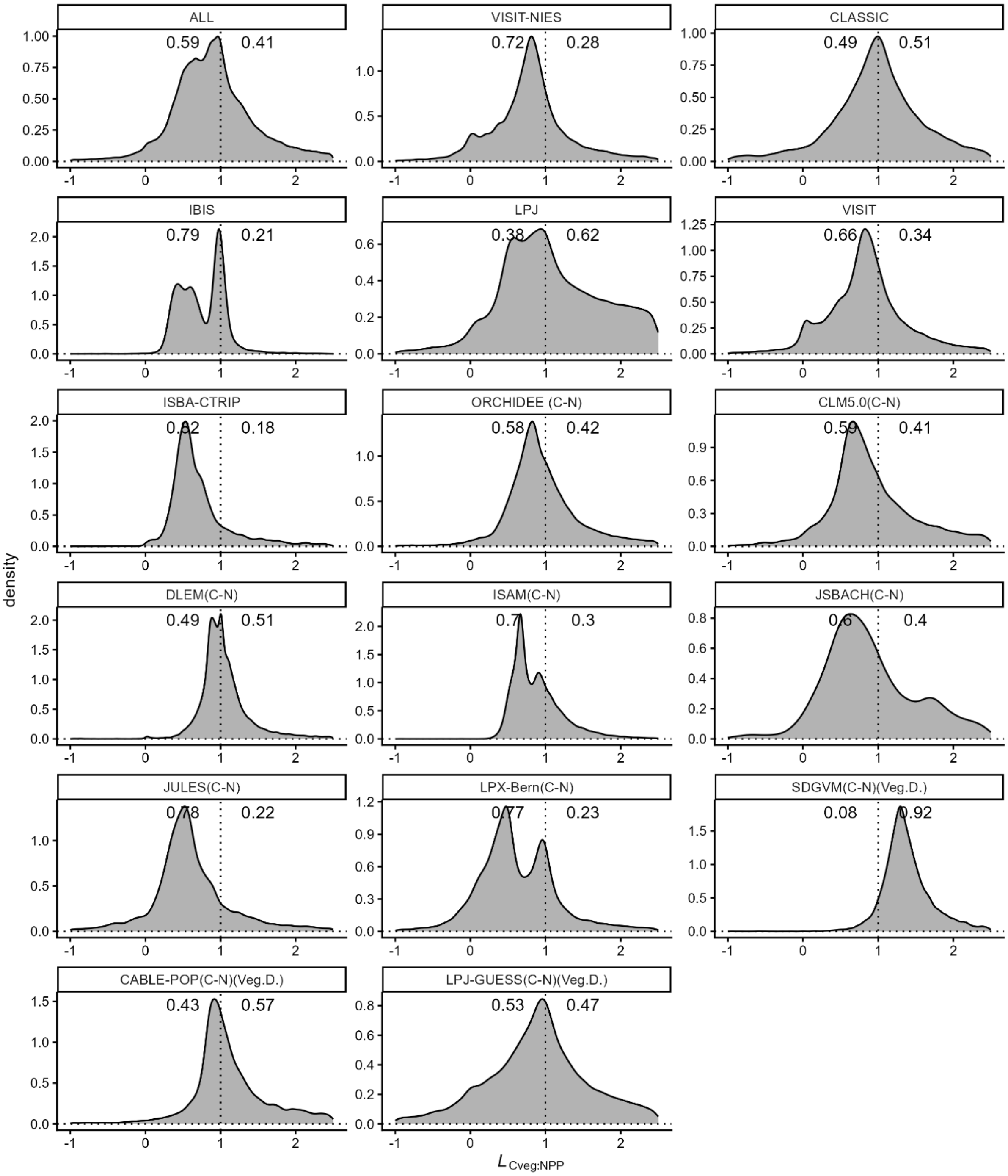

Figure S19 Similar to Figure S4. The only difference is the steady state estimation of C_{veg}* in Figure S4 but not in this figure (see method). The figure is provided for visualising the effect of steady state estimation. We are certain that Figure S4 is more reasonable than this one.

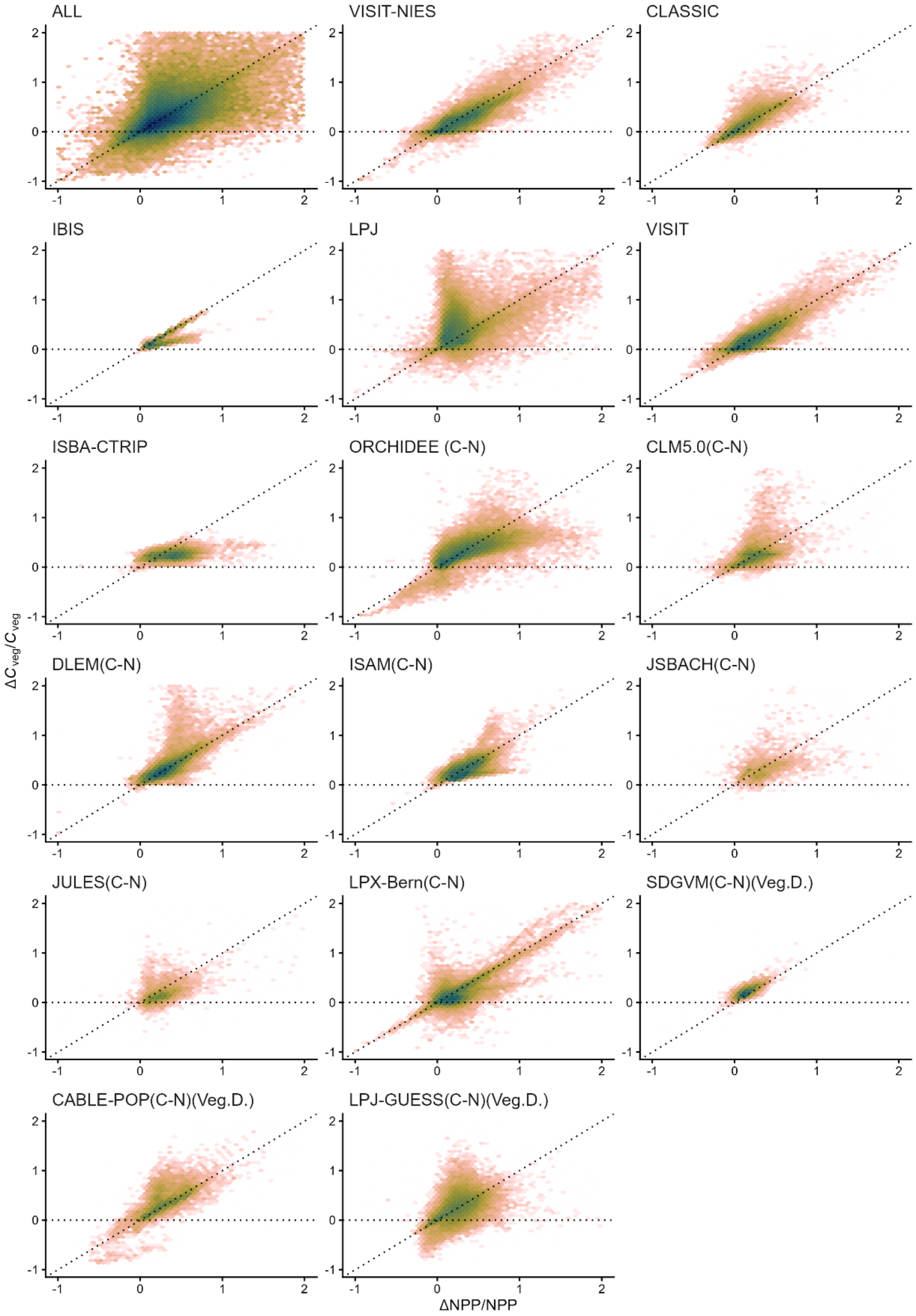

Figure S20 Similar to Figure S5. The only difference is the steady state estimation of C_{veg}* in Figure S5 but not in this figure (see method). The figure is provided for visualising the effect of steady state estimation. We are certain that Figure S5 is more reasonable than this one.

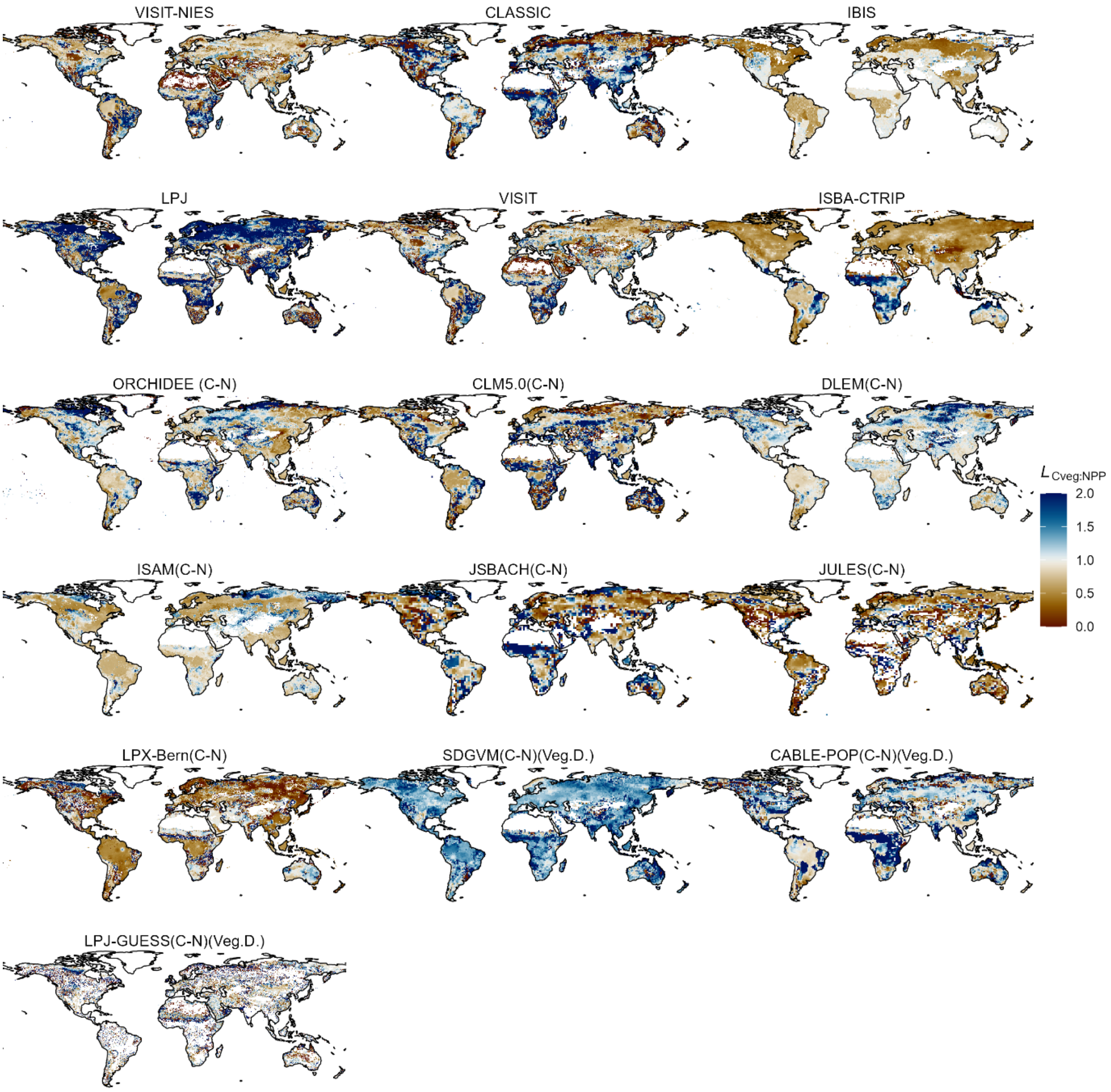

Figure S21 Similar to Figure S6. The only difference is the steady state estimation of C_{veg}* in Figure S6 but not in this figure (see method). The figure is provided for visualising the effect of steady state estimation. We are certain that Figure S6 is more reasonable than this one.

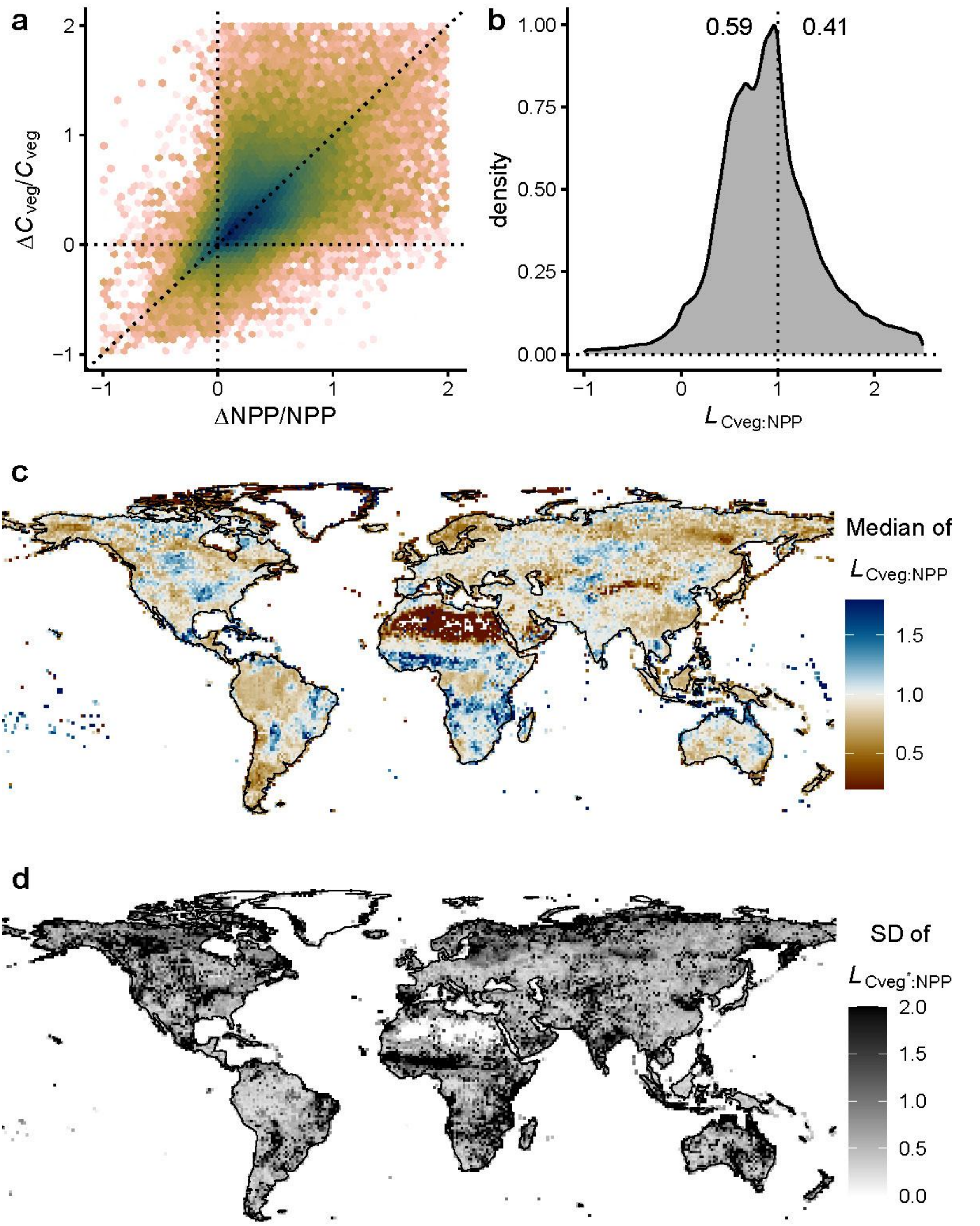

Figure S22 Similar to Figure 4. The only difference is the steady state estimation of C_{veg}* in Figure 4 but not in this figure (see method). The figure is provided for visualising the effect of steady state estimation. We are certain that Figure 4 is more reasonable than this one.

8. Other figures

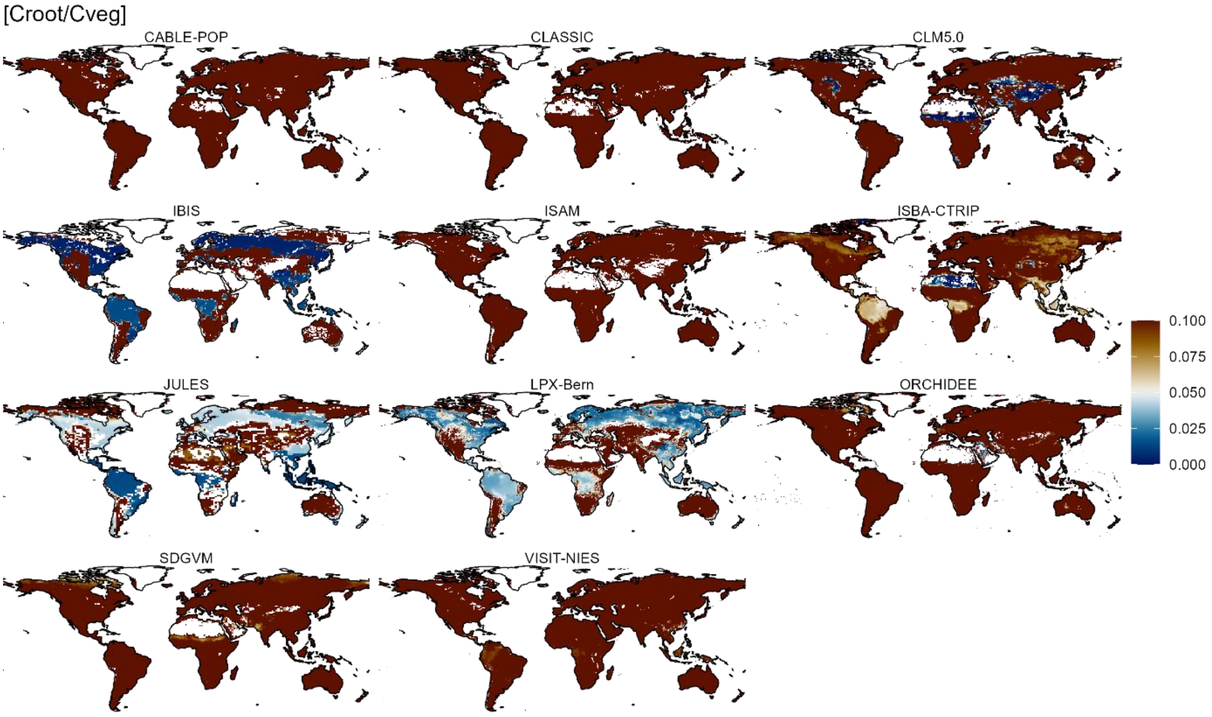

Figure S23 Root biomass (C_{root}) divided by vegetation biomass (C_{veg}) for each model. This figure is used to diagnose which model includes coarse roots in output variable C_{root}. The idea is that for tropical evergreen forests, C_{root} / C_{veg} > 5% would signal that coarse root is included in C_{root}. We did try to find out whether the model outputs of C_{root} include coarse roots, by reading manuals and contracting modelers. However, some modelers just can't remember or they are not responsible for roots simulation. Such information may be archived somewhere in the output explanation of the model manual. In this case, the only risk is that the model manual was not written for TRENDY and the TRENDY outputs could be in a different format. If unfortunately not explained in the output part, it would be somewhere in the codes or equations which will take a whole day to find out (an example of model opaqueness). In any case, the most reliable and efficient way to find out, is by diagnosing the output and draw C_{root} / C_{veg}

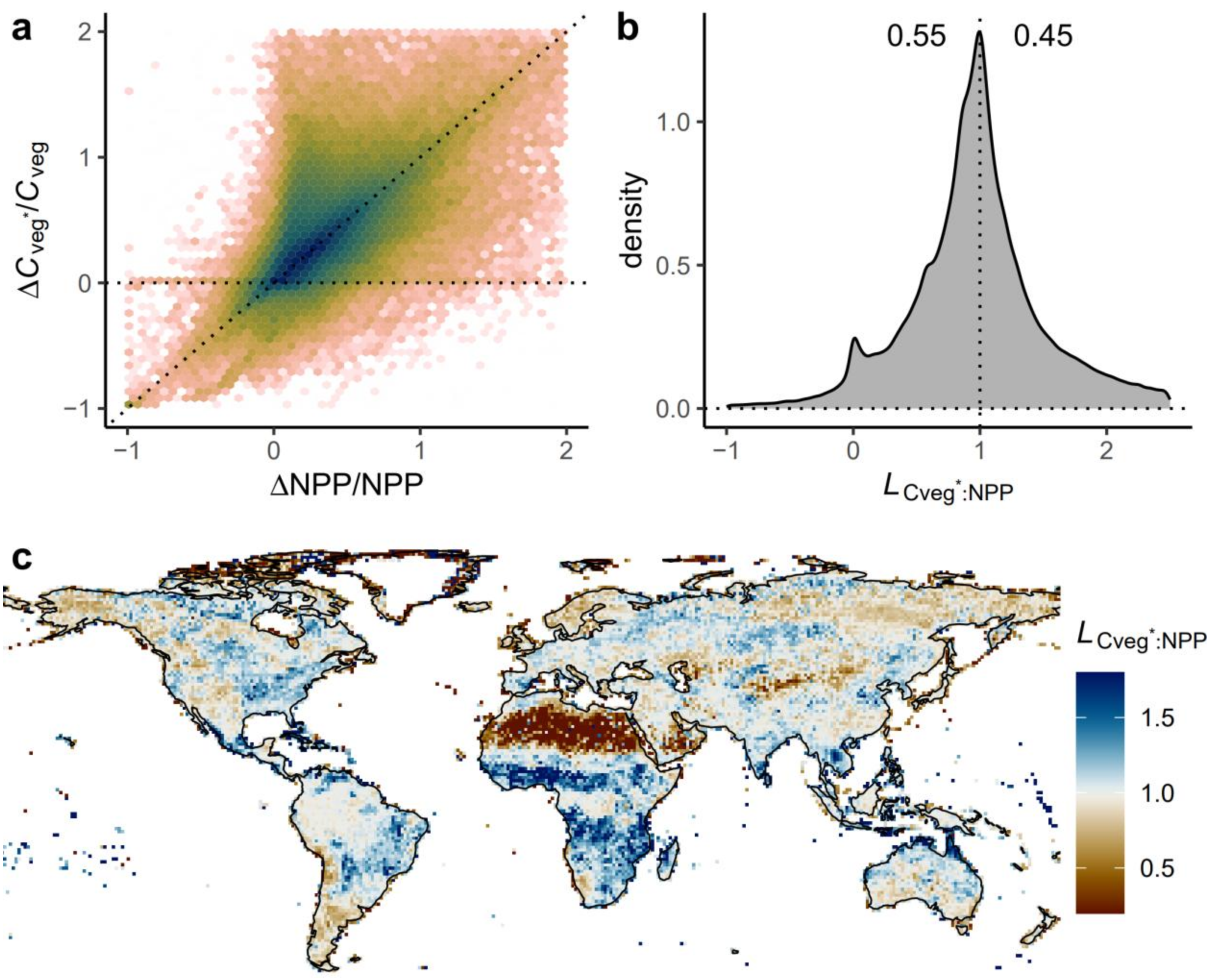

Figure S24 The relationship between the relative change of steady-state vegetation C (R_{Cveg*} = $\Delta C_{veg*}/C_{veg}$) and the relative change of net primary productivity (R_{NPP} = ΔNPP/NPP). Same figure as Figure 4 (in the main text) but here we include gridcells with C_{veg} lower than 5% quantile (i.e. include sparse desert). By comparing with Figure 4, it could be concluded that small C_{veg} values lead to a spike at of $L_{Cveg*:NPP} = 0$.

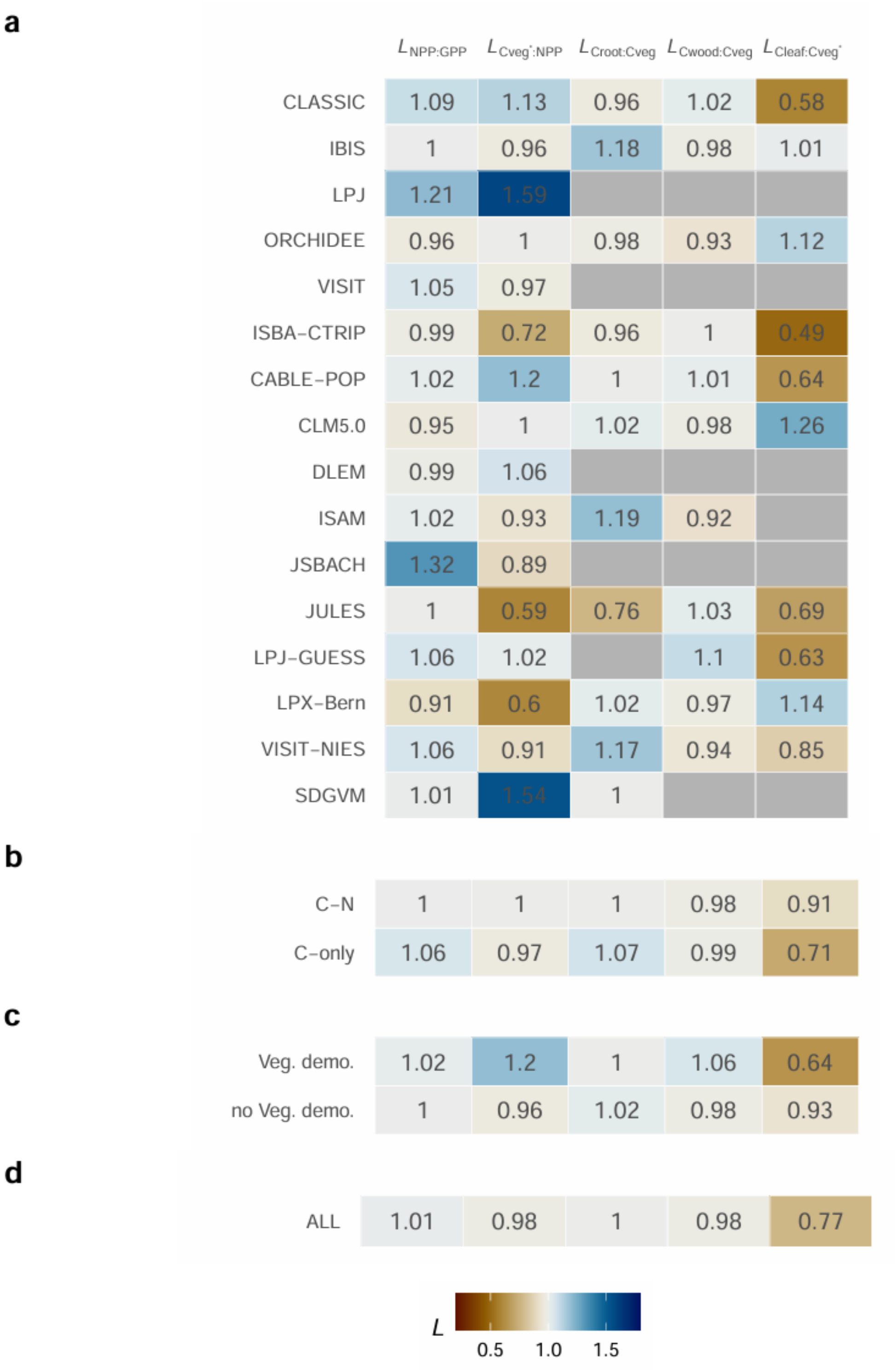

Figure S25 Redo figure 2 with the period **<u>2008 – 2017</u>** instead of 2013 – 2022, while keeping everything else unchanged. This is done as a sensitivity test to check the effect of chosen time period. This figure is very similar to Figure 2, implying that the chosen time period would not affect emergent patterns in *L*.

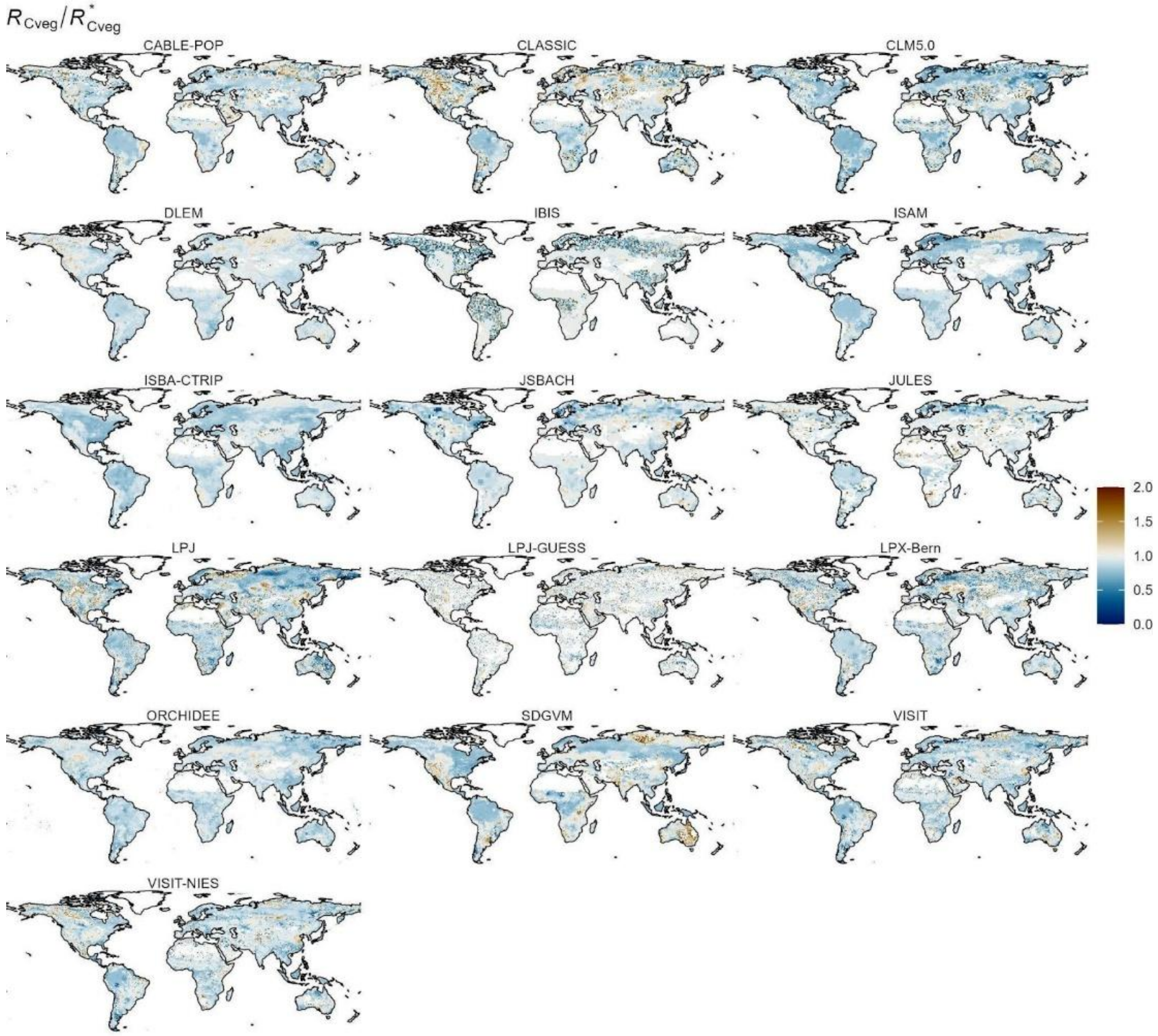

Figure S26 Difference between the relative change of total vegetation C with steady state correction (R_{Cveg}^*) and without correction (R_{Cveg}).

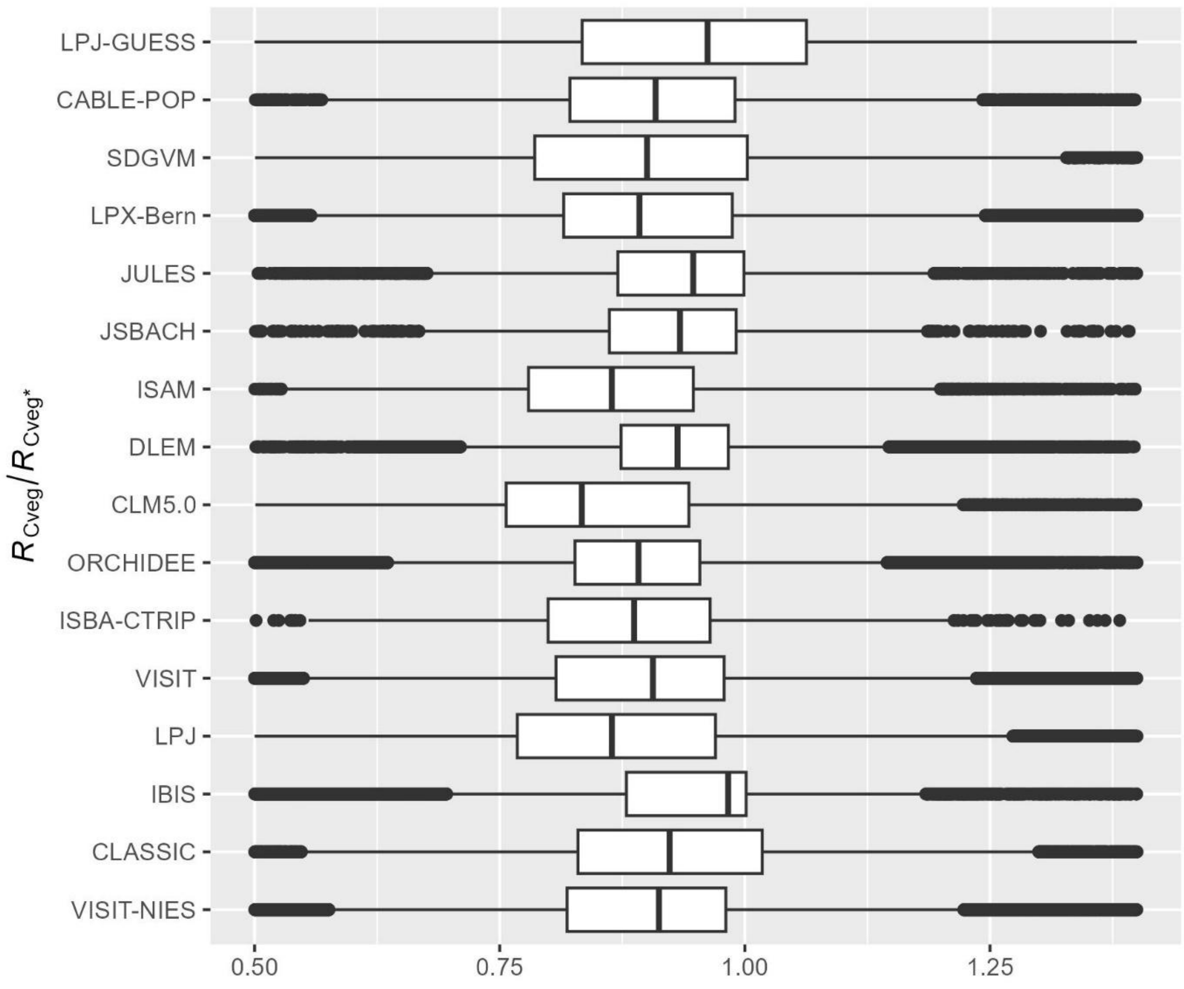

Figure S27 Difference between the relative change of total vegetation C with steady state correction (RC_{veg}^*) and without correction (RC_{veg}).

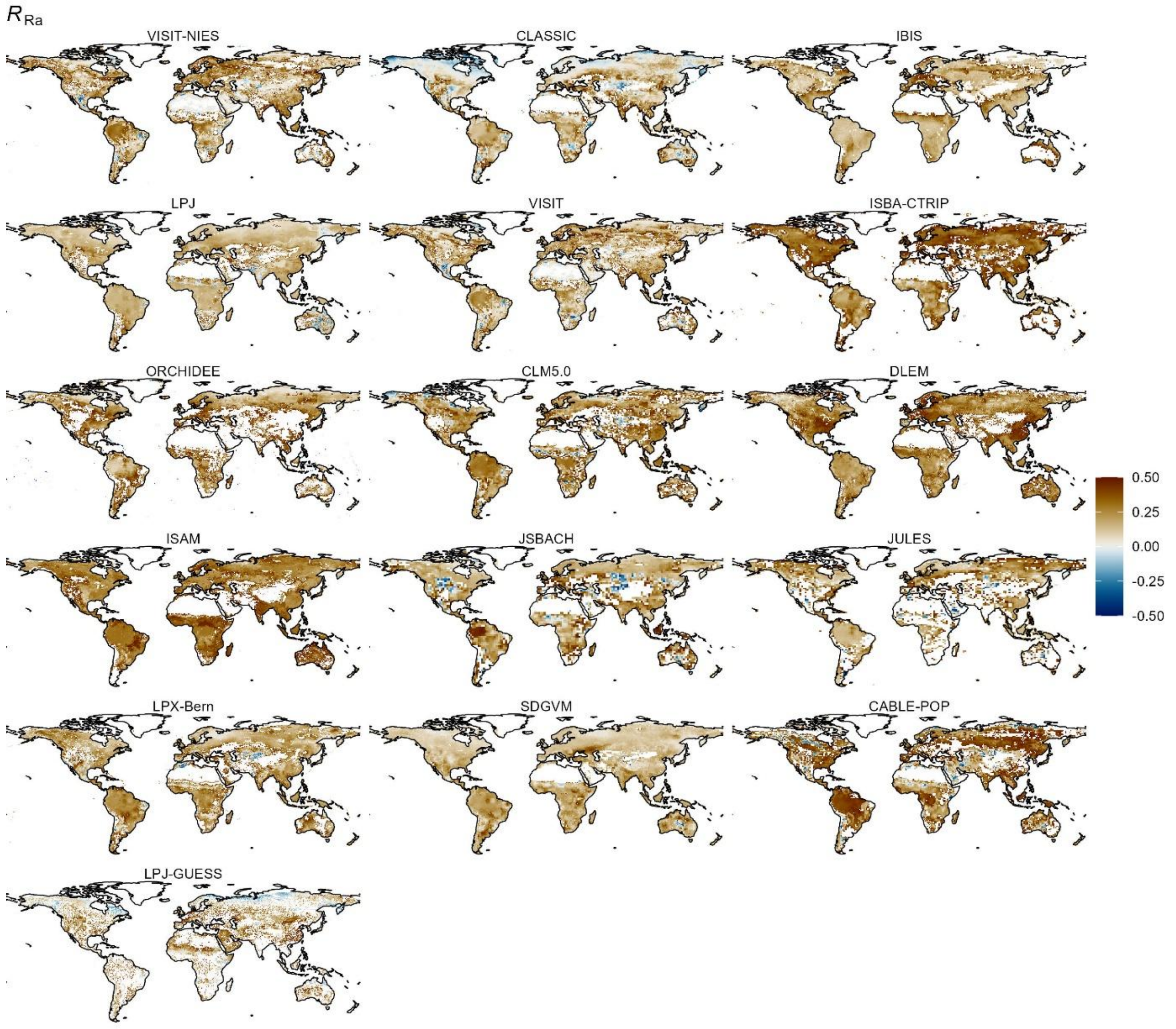

Figure S28 Relative change in autotrophic respiration (R_a).

Supplementary Data 1 available online Median and inter-quantile range (IQR) for each relative change and linearity term. This table is inserted here as a screenshot to remind readers that a full table of *L* and *R* is available as Supplementary Data.

modl	CN_couple	Veg_Demo	R_cveg_me	R_cveg_IQ	R_cveg_ag	R_cveg_ag	R_npp_me	R_npp_IQR
ALL	NA	NA	0.221319	0.253087	0.192046	0.275396	0.23961	0.266594
C only moc	NA	NA	0.204468	0.242747	0.176611	0.201568	0.223317	0.285469
CN couplec	NA	NA	0.230395	0.258707	0.214351	0.342586	0.246346	0.253423
Veg Demo	NA	NA	0.213737	0.25112	0.311444	0.408278	0.176249	0.225011
not Veg De	NA	NA	0.222964	0.255043	0.177357	0.233673	0.252861	0.268374
CLASSIC	No	No	0.140969	0.350509	0.158927	0.370991	0.118609	0.286932
IBIS	No	No	0.118634	0.112425	0.120871	0.115984	0.169287	0.158311
LPJ	No	No	0.351986	0.422578	NA	NA	0.226549	0.230752
ORCHIDEE	Yes	No	0.288228	0.292024	0.270479	0.302834	0.286263	0.36154
VISIT	No	No	0.186924	0.257432	NA	NA	0.193255	0.276742
ISBA-CTRIP	No	No	0.232062	0.091553	0.208561	0.101355	0.390354	0.209443
CABLE-POF	Yes	Yes	0.352245	0.362328	0.371886	0.361812	0.2772	0.310531
CLM5.0	Yes	No	0.242684	0.219143	0.243813	0.269175	0.250044	0.230235
DLEM	Yes	No	0.272836	0.164892	NA	NA	0.281312	0.160464
ISAM	Yes	No	0.242277	0.182337	NA	NA	0.308257	0.170315
JSBACH	Yes	No	0.34529	0.357236	NA	NA	0.371669	0.396924
JULES	Yes	No	0.154277	0.262207	0.161025	0.26752	0.255006	0.23419
LPJ-GUESS	Yes	Yes	0.23097	0.348865	0.239032	0.43015	0.238929	0.268925
LPX-Bern	Yes	No	0.095996	0.160104	0.094712	0.16833	0.176912	0.158454
VISIT-NIES	No	No	0.214673	0.290519	0.200631	0.277951	0.253023	0.34182
SDGVM	Yes	Yes	0.170798	0.075533	NA	NA	0.123461	0.057336

9. Sensitivity test for steady state correction on Cwood and Croot.

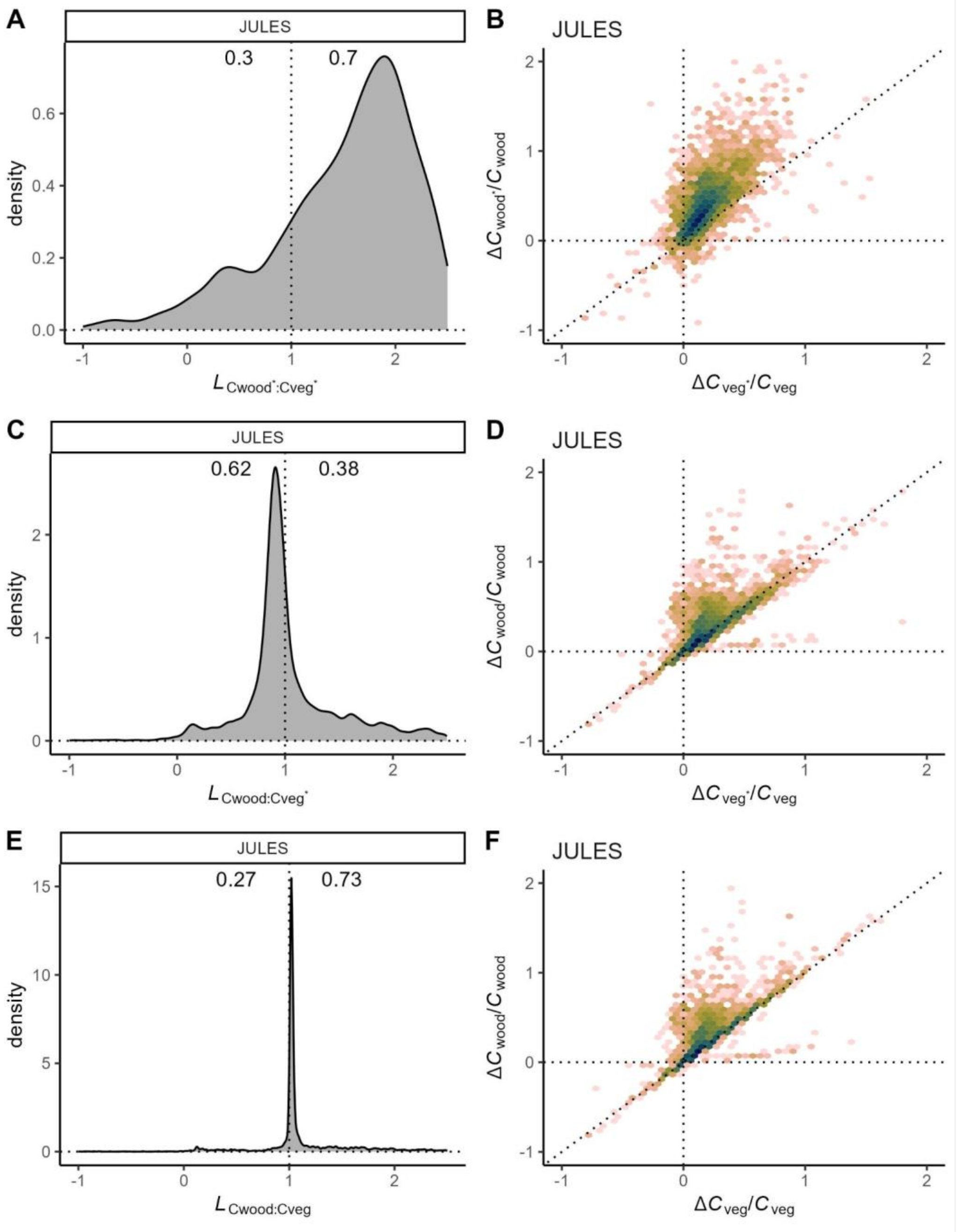

Figure S29 Redo figure S16 and S17 but with steady state correction for Cwood. We can only do this for JULES only because of the availability of relevant output variables.

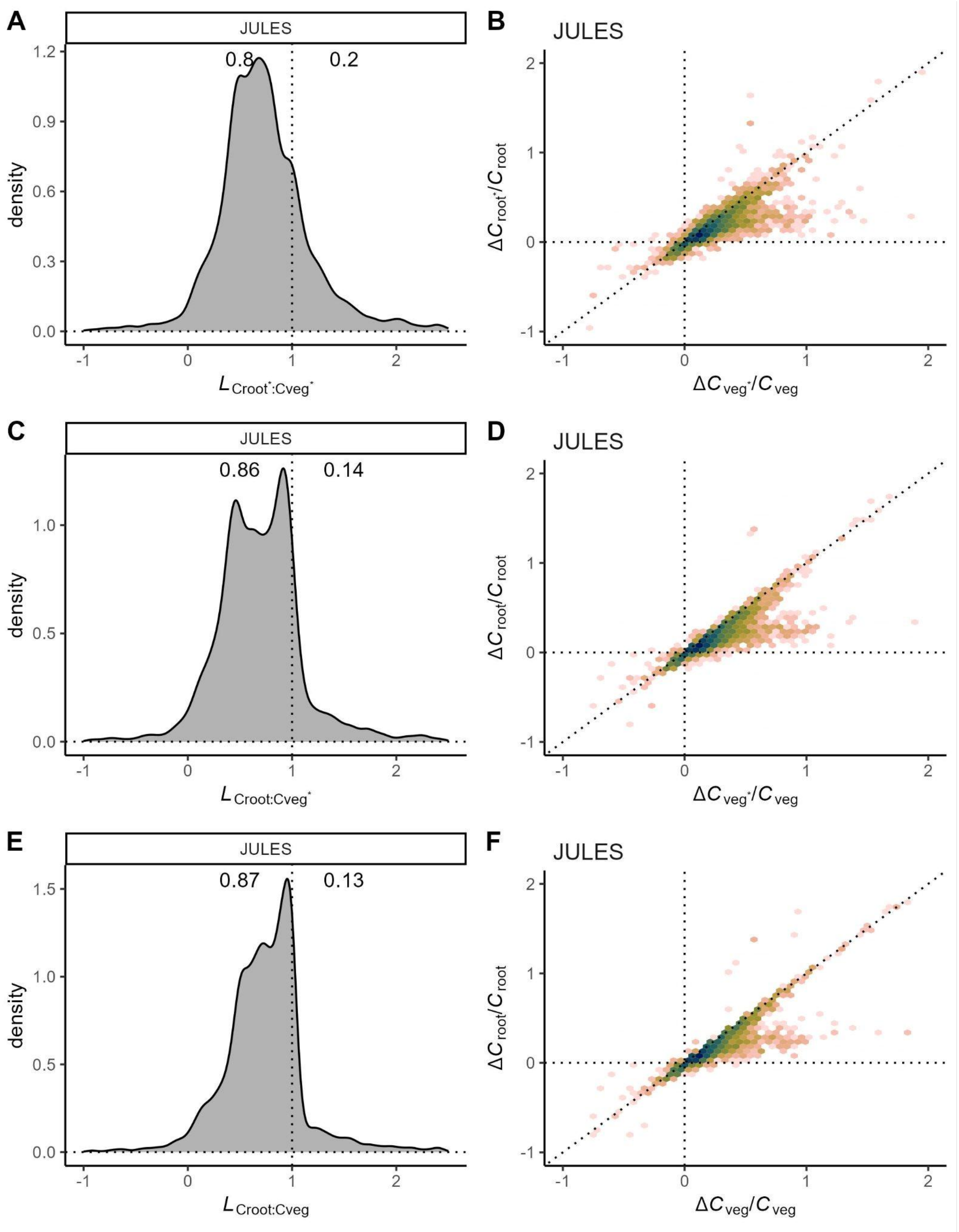

Figure S30 Redo figure S7 and S8 but with steady state correction for Croot. We can do this for JULES only because of the availability of relevant output variables. None of the TRENDY v11 models provided NPP_{root} or NPP_{wood} so steady state correction for root and wood is not possible. However, JULES provides fWoodLitter (which is the carbon flux leaving the wood pool) and fRootLitter (which is the carbon flux leaving the root pool). Therefore we can get NPP_{root} using:

NPP_{root} = dCroot/dt + fRootLitter

Where root NPP equals the change in Croot pool plus fRootLitter.

Then C_{root}* can be calculated following equation (15). The same procedure can be applied for calculating C_{wood}* using fWoodLitter outputs. This allows calculations of $L_{Croot*:Cveg*}$ and $L_{Cwood*:Cveg*}$.

Before interpreting Figures S29 S30, it is important to note, according to equation (55) in (Clark et al., 2011), that C_{root} = C_{leaf} in JULES. C_{root} is fine root only (Figure S23). All other models have coarse roots in C_{root} . C_{veg} is allocated to C_{wood} using constant parameters (except the dynamic in LAI) so $L_{Cwood*:Cveg*}$ should be very linear. Carbon allocation is fairly simple in JULES so we can expect it to be linear.

For $L_{Croot:Cveg}$ (Figure S30), the steady state correction does not change conclusions about linearity patterns. All panels deliver similar messages. Figure S30c S30d looks exactly the same as $L_{Cleaf:Cveg*}$ (Figure S13), so the results are consistent with JULES technical manual. For $L_{Cwood:Cveg}$ however, the linearity shows up strongly without correction. Therefore, no correction is likely the best option. The median of $L_{Cwood*:Cveg*}$ is about 2, meaning R_{Cwood*} = 2 x R_{Cveg*} (C_{wood} increase twice more than C_{veg}) which is supernatural. This means that the calculation from fWoodLitter is wrong because of mortality event at the end of the simulation. To conclude, $L_{Cwood:Cveg}$ (no steady state) is the best option. The steady state correction for $L_{Croot:Cveg}$ (Figure S30) barely affect the results, because root in JULES is fine root only. Other models have coarse roots included in the root. In this case, root turnover time is very long like C_{wood}, so $L_{Croot:Cveg}$ (no steady state like Figure S7) should be the best option.

Ainsworth, E. A., & Long, S. P. (2005). What have we learned from 15 years of free-air CO2 enrichment (FACE)? A meta-analytic review of the responses of photosynthesis, canopy properties and plant production to rising CO2. *New Phytologist*, *165*(2), 351–372. https://doi.org/10.1111/j.1469-8137.2004.01224.x

Brienen, R. J. W., Caldwell, L., Duchesne, L., Voelker, S., Barichivich, J., Baliva, M., Ceccantini, G., Di Filippo, A., Helama, S., Locosselli, G. M., Lopez, L., Piovesan, G., Schöngart, J., Villalba, R., & Gloor, E. (2020). Forest carbon sink neutralized by pervasive growth-lifespan trade-offs. *Nat. Commun.*, *11*(1), 4241.

Brienen, R. J. W., Phillips, O. L., Feldpausch, T. R., Gloor, E., Baker, T. R., Lloyd, J., Lopez-Gonzalez, G., Monteagudo-Mendoza, A., Malhi, Y., Lewis, S. L., Vásquez Martinez, R., Alexiades, M., Álvarez Dávila, E., Alvarez-Loayza, P., Andrade, A., Aragão, L. E. O. C., Araujo-Murakami, A., Arets, E. J. M. M., Arroyo, L., … Zagt, R. J. (2015). Long-term decline of the Amazon carbon sink. *Nature*, *519*(7543), 344–348. https://doi.org/10.1038/nature14283

Bugmann, H., & Bigler, C. (2011). Will the CO2 fertilization effect in forests be offset by reduced tree longevity? *Oecologia*, *165*(2), 533–544. https://doi.org/10.1007/s00442-010-1837-4

Clark, D. B., Mercado, L. M., Sitch, S., Jones, C. D., Gedney, N., Best, M. J., Pryor, M., Rooney, G. G., Essery, R. L. H., Blyth, E., Boucher, O., Harding, R. J., Huntingford, C., & Cox, P. M. (2011). The Joint UK Land Environment Simulator (JULES), model description – Part 2: Carbon fluxes and vegetation dynamics. *Geoscientific Model Development*, *4*(3), 701–722. https://doi.org/10.5194/gmd-4-701-2011

Curtis, P. S., & Wang, X. (1998). A meta-analysis of elevated CO 2 effects on woody plant mass, form, and physiology. *Oecologia*, *113*(3), 299–313. https://doi.org/10.1007/s004420050381

De Kauwe, M. G., Medlyn, B. E., Zaehle, S., Walker, A. P., Dietze, M. C., Wang, Y., Luo, Y., Jain, A. K., El-Masri, B., Hickler, T., Wårlind, D., Weng, E., Parton, W. J., Thornton, P. E., Wang, S., Prentice, I. C., Asao, S., Smith, B., McCarthy, H. R., … Norby, R. J. (2014). Where does the carbon go? A model–data intercomparison of vegetation carbon allocation and turnover processes at two temperate forest free-air CO_2 enrichment sites. *New Phytologist*, *203*(3), 883–899. https://doi.org/10.1111/nph.12847

Franklin, O. (2007). Optimal nitrogen allocation controls tree responses to elevated CO_2. *New Phytologist*, *174*(4), 811–822. https://doi.org/10.1111/j.1469-8137.2007.02063.x

Fransson, P. (2012). Elevated CO2 impacts ectomycorrhiza-mediated forest soil carbon flow: Fungal biomass production, respiration and exudation. *Fungal Ecology*, *5*(1), 85–98. https://doi.org/10.1016/j.funeco.2011.10.001

Gielen, B., Calfapietra, C., Lukac, M., Wittig, V. E., De Angelis, P., Janssens, I. A., Moscatelli, M. C., Grego, S., Cotrufo, M. F., Godbold, D. L., Hoosbeek, M. R., Long, S. P., Miglietta, F., Polle, A., Bernacchi, C. J., Davey, P. A., Ceulemans, R., & Scarascia-Mugnozza, G. E. (2005). Net carbon storage in a poplar plantation (POPFACE) after three years of free-air CO2 enrichment. *Tree Physiology*, *25*(11), 1399–1408. https://doi.org/10.1093/treephys/25.11.1399

Han, X., Zhou, G., Luo, Q., Ferlian, O., Zhou, L., Meng, J., Qi, Y., Pei, J., He, Y., Liu, R., Du, Z., Long, J., Zhou, X., & Eisenhauer, N. (2023). Plant biomass responses to elevated CO2 are mediated by phosphorus uptake. *Science of The Total Environment*, *863*, 160775. https://doi.org/10.1016/j.scitotenv.2022.160775

Jiang, M., Medlyn, B. E., Drake, J. E., Duursma, R. A., Anderson, I. C., Barton, C. V. M., Boer, M. M., Carrillo, Y., Castañeda-Gómez, L., Collins, L., Crous, K. Y., De Kauwe, M. G., dos Santos, B. M., Emmerson, K. M., Facey, S. L., Gherlenda, A. N., Gimeno,

T. E., Hasegawa, S., Johnson, S. N., … Ellsworth, D. S. (2020). The fate of carbon in a mature forest under carbon dioxide enrichment. *Nature*, *580*(7802). https://doi.org/10.1038/s41586-020-2128-9

Körner, C. (2017). A matter of tree longevity. *Science*, *355*(6321), 130–131.

Leakey, A. D. B., Ainsworth, E. A., Bernacchi, C. J., Rogers, A., Long, S. P., & Ort, D. R. (2009). Elevated CO2 effects on plant carbon, nitrogen, and water relations: Six important lessons from FACE. *Journal of Experimental Botany*, *60*(10), 2859–2876. https://doi.org/10.1093/jxb/erp096

Marqués, L., Weng, E., Bugmann, H., Forrester, D. I., Rohner, B., Hobi, M. L., Trotsiuk, V., & Stocker, B. D. (2023). Tree Growth Enhancement Drives a Persistent Biomass Gain in Unmanaged Temperate Forests. *AGU Advances*, *4*(5), e2022AV000859. https://doi.org/10.1029/2022AV000859

Norby, R. J., Loader, N. J., Mayoral, C., Ullah, S., Curioni, G., Smith, A. R., Reay, M. K., van Wijngaarden, K., Amjad, M. S., Brettle, D., Crockatt, M. E., Denny, G., Grzesik, R. T., Hamilton, R. L., Hart, K. M., Hartley, I. P., Jones, A. G., Kourmouli, A., Larsen, J. R., … MacKenzie, A. R. (2024). Enhanced woody biomass production in a mature temperate forest under elevated CO2. *Nature Climate Change*, *14*(9), 983–988. https://doi.org/10.1038/s41558-024-02090-3

Norby, R. J., Warren, J. M., Iversen, C. M., Medlyn, B. E., & McMurtrie, R. E. (2010). CO2 enhancement of forest productivity constrained by limited nitrogen availability. *Proceedings of the National Academy of Sciences*, *107*(45), 19368–19373.

Norby, R. J., & Zak, D. R. (2011). Ecological Lessons from Free-Air CO2 Enrichment (FACE) Experiments. *Annual Review of Ecology, Evolution, and Systematics*, *42*(1), 181–203. https://doi.org/10.1146/annurev-ecolsys-102209-144647

Phillips, R. P., Meier, I. C., Bernhardt, E. S., Grandy, A. S., Wickings, K., & Finzi, A. C. (2012). Roots and fungi accelerate carbon and nitrogen cycling in forests exposed to elevated CO_2. *Ecology Letters*, *15*(9), 1042–1049. https://doi.org/10.1111/j.1461-0248.2012.01827.x

Rogers, H. H., Prior, S. A., Runion, G. B., & Mitchell, R. J. (1995). Root to shoot ratio of crops as influenced by CO2. *Plant and Soil*, *187*(2), 229–248. https://doi.org/10.1007/BF00017090

Smith, N. G. (2017). Plant Respiration Responses to Elevated CO2: An Overview from Cellular Processes to Global Impacts. In G. Tcherkez & J. Ghashghaie (Eds.), *Plant Respiration: Metabolic Fluxes and Carbon Balance* (pp. 69–87). Springer International Publishing. https://doi.org/10.1007/978-3-319-68703-2_4

Stocker, B. D., Dong, N., Perkowski, E. A., Schneider, P. D., Xu, H., De Boer, H. J., Rebel, K. T., Smith, N. G., Van Sundert, K., Wang, H., Jones, S. E., Prentice, I. C., & Harrison, S. P. (2024). Empirical evidence and theoretical understanding of ecosystem carbon and nitrogen cycle interactions. *New Phytologist*, nph.20178. https://doi.org/10.1111/nph.20178

Terrer, C., Phillips, R. P., Hungate, B. A., Rosende, J., Pett-Ridge, J., Craig, M. E., van Groenigen, K. J., Keenan, T. F., Sulman, B. N., & Stocker, B. D. (2021). A trade-off between plant and soil carbon storage under elevated CO2. *Nature*, *591*(7851), 599–603.

Van Sundert, K., Leuzinger, S., Bader, M. K. -F., Chang, S. X., De Kauwe, M. G., Dukes, J. S., Langley, J. A., Ma, Z., Mariën, B., Reynaert, S., Ru, J., Song, J., Stocker, B., Terrer, C., Thoresen, J., Vanuytrecht, E., Wan, S., Yue, K., & Vicca, S. (2023). When things get MESI: The Manipulation Experiments Synthesis Initiative—A coordinated effort to synthesize terrestrial global change experiments. *Global Change Biology*, *29*(7), 1922–1938. https://doi.org/10.1111/gcb.16585

Walker, A. P., De Kauwe, M. G., Bastos, A., Belmecheri, S., Georgiou, K., Keeling, R. F., McMahon, S. M., Medlyn, B. E., Moore, D. J. P., Norby, R. J., Zaehle, S., Anderson-Teixeira, K. J., Battipaglia, G., Brienen, R. J. W., Cabugao, K. G., Cailleret, M., Campbell, E., Canadell, J. G., Ciais, P., … Zuidema, P. A. (2021). Integrating the evidence for a terrestrial carbon sink caused by increasing atmospheric CO2. *New Phytologist*, *229*(5), 2413–2445. https://doi.org/10.1111/nph.16866

Walker, A. P., De Kauwe, M. G., Medlyn, B. E., Zaehle, S., Iversen, C. M., Asao, S., Guenet, B., Harper, A., Hickler, T., Hungate, B. A., Jain, A. K., Luo, Y., Lu, X., Lu, M., Luus, K., Megonigal, J. P., Oren, R., Ryan, E., Shu, S., … Norby, R. J. (2019). Decadal biomass increment in early secondary succession woody ecosystems is increased by CO2 enrichment. *Nature Communications*, *10*(1), 454. https://doi.org/10.1038/s41467-019-08348-1

Weng, E., Dybzinski, R., Farrior, C. E., & Pacala, S. W. (2019). Competition alters predicted forest carbon cycle responses to nitrogen availability and elevated CO_2: Simulations using an explicitly competitive, game-theoretic vegetation demographic model. *Biogeosciences*, *16*(23), 4577–4599. https://doi.org/10.5194/bg-16-4577-2019

Steady-state estimation

Beni Stocker

2026-04-09

This is to assess the accuracy of the steady-state estimation from transient model outputs, in support of Zhang-Zheng et al. (*in rev.*).

We consider three alternative models of the C cycle in a terrestrial ecosystem. The first represents all pool dynamics as a first-order decay process (constant turnover) and assumes constant parameters governing the downstream partition of C flows, determining the ratio of NPP/GPP and allocation fractions. Therefore, by design, it exhibits linear dynamics.

The second model is a modified version of the first, where the turnover time of the wood C pool shortens as the size of that pool increases beyond a critical threshold. This introduces a non-linearity.

The third model, BiomeEP, is a vegetation demography model that explicity simulates forest dynamics. Previous research has demonstrated that upon a change in biomass production (or NPP), steady-state biomass pools change in parallel, but to a lesser degree (in relative terms) (Marqués et al., 2023). The model thus exhibits non-linear dynamics, arising from the relationship between NPP and steady-state vegetation C pools.

This vignette evaluates estimated versus actual steady-state vegetation C pools ($C(t)$) based on the three alternative models. Steady-state vegetation C pools are estimated using an approximative function.

Analytic approach

The steady-state vegetation C pool (C^*) can be estimated, assuming first-order decay (constant turnover τ), as:

$$C^* = \text{NPP}(t)/\tau$$

Also following the first-order decay model, the temporal change in $C(t)$ is given by:

$$dC/dt = \text{NPP}(t) - C(t)/\tau$$

Combining these two equations, the turnover rate (τ) can be estimated from NPP, the current (transiently changing) size ($C(t)$) and the temporal change of the vegetationa C pool (dC/dt):

$$\tau = \frac{C(t)}{\text{NPP}(t) - dC/dt}$$

This, in turn can be plugged into the first equation here. Thus, we get an expression for estimating of the steady-state vegetation C pool size from the transient dynamics as:

$$C^* = \frac{C(t) \times \text{NPP}(t)}{\text{NPP}(t) - dC/dt}$$

Eq. 12 is applied in the following for estimating the steady-state vegetation C pool size C^* from the transient simulation outputs.

The estimated steady state (C^*) serves to yield a more reliable quantification of the linearity than when relying on transient values $C(t)$.

The linearity term L is defined as

$$L_{y:x} = R_y/R_x = \frac{\Delta y/y}{\Delta x/x}$$

Here, y is the vegetation C pool ($C(t)$) and x is NPP. The Δ shall be derived from outputs of transient model simulations, comparing the initial state from the start of the transient simulation to the final state, either at the end of the transient simulation (for fluxes, e.g., NPP), or as an estimate of the steady-state for pools.

Simulations

Load R libraries.

```
library(tidyverse)

## Warning: package 'ggplot2' was built under R version 4.5.3

## Warning: package 'tidyr' was built under R version 4.5.3

## Warning: package 'purrr' was built under R version 4.5.3

## Warning: package 'dplyr' was built under R version 4.5.3

## ── Attaching core tidyverse packages ──────────────────────── tidyverse 2.
0.0 ──
## ✔ dplyr     1.2.1     ✔ readr     2.1.5
## ✔ forcats   1.0.0     ✔ stringr   1.5.1
## ✔ ggplot2   4.0.3     ✔ tibble    3.3.0
## ✔ lubridate 1.9.4     ✔ tidyr     1.3.2
## ✔ purrr     1.2.2
## ── Conflicts ────────────────────────────────────────── tidyverse_conflict
s() ──
## ✖ dplyr::filter() masks stats::filter()
## ✖ dplyr::lag()    masks stats::lag()
## ℹ Use the conflicted package (<http://conflicted.r-lib.org/>) to force all
 conflicts to become errors
```

```r
library(rsofun)  # contains BiomeEP model
```

```
## Warning: package 'rsofun' was built under R version 4.5.3
```

```r
library(slider)
```

```
## Warning: package 'slider' was built under R version 4.5.3
```

```r
library(knitr)
library(khroma)
```

Implement steady state estimation function (in R).

```r
estimate_steady_state <- function(c_pool, c_in, dc){
  (c_pool * c_in)/(c_in - dc)
}
```

Linear model

Define model

The model is implemented in R and described in more detail here.

```r
ccascade <- function(
  gpp,
  state = initpools(),
  par = getpar(),
  fluxes = initfluxes()
  ){

  # determine integration length (number of time steps) from length of 'c_inf
lux'
  len <- length(gpp)

  # output the state and fluxes in one data frame
  df <- dplyr::tibble()

  # integrate over each time step (this is an implementation of the different
ial equation)
  for (yr in seq(len)){

    # update states and fluxes
    out <- ccascade_onestep(gpp[yr], state, par, fluxes)
    state <- out$state
    fluxes <- out$fluxes

    # record for output
    df <- dplyr::bind_rows(
      df,
      dplyr::bind_cols(
        dplyr::tibble(year = yr),
```

```
        dplyr::as_tibble(state),
        dplyr::as_tibble(fluxes))
      )
  }
  return(df)
}

ccascade_onestep <- function(gpp, state, par, fluxes){

  # total biomass production
  bp <- gpp * par$bpe
  fluxes$ra <- gpp * (1 - par$bpe)

  # biomass turnover by pool, use exponential here to avoid numerical instabi
lity with k -> 1
  dleaf <- (1 - exp(-par$kleaf)) * state$cleaf
  dwood <- (1 - exp(-par$kwood)) * state$cwood
  droot <- (1 - exp(-par$kroot)) * state$croot

  # update biomass pools
  state$cleaf <- state$cleaf + bp * par$fleaf - dleaf
  state$cwood <- state$cwood + bp * par$fwood - dwood
  state$croot <- state$croot + bp * par$froot - droot

  # litter turnover
  dflitt <- (1 - exp(-par$kflitt)) * state$flitt
  dslitt <- (1 - exp(-par$kslitt)) * state$slitt

  # update litter: add biomass turnover and update litter pools
  state$flitt <- state$flitt + dleaf + droot - dflitt
  state$slitt <- state$slitt + dwood         - dslitt

  # heterotrophic respiration during litter decomposition
  rh_litt <- (1 - par$eff) * (dflitt + dslitt)

  # soil turnover
  dfsoil <- (1 - exp(-par$kfsoil)) * state$fsoil
  dssoil <- (1 - exp(-par$kssoil)) * state$ssoil

  # update soil: remainder of litter turnover goes to soil, subtract decompos
ing portion
  state$fsoil <- state$fsoil + par$eff * par$ffast       * (dflitt + dslitt)
- dfsoil
  state$ssoil <- state$ssoil + par$eff * (1 - par$ffast) * (dflitt + dslitt)
- dssoil

  # heterotrophic respiration from soil decomposition
  rh_soil <- dfsoil + dssoil
```

```
  fluxes$rh <- rh_litt + rh_soil

  return(list(state = state, fluxes = fluxes))
}

getpar <- function(){
  list(
    bpe = 0.4,
    fleaf = 0.3,
    froot = 0.3,
    fwood = 0.4,
    kleaf = 0.5,
    kwood = 0.02,
    kroot = 0.5,
    kflitt = 0.5,
    kslitt = 0.1,
    kfsoil = 0.1,
    kssoil = 0.003,
    eff = 0.6,
    ffast = 0.95
    )
}

initpools <- function(){
  list(
    cleaf = 0,
    cwood = 0,
    croot = 0,
    flitt = 0,
    slitt = 0,
    fsoil = 0,
    ssoil = 0
    )
}

initfluxes <- function(){
  list(
    ra = 0,
    rh = 0
    )
}
```

Create forcing

Create model forcing: 1000 years spinup at CO2 levels corresponding to year 1700 (first year of transient simulation). Then transiently changing CO2 following observations (ISIMIP2a standard forcing file) up to year 2018. Then 1000 simulation years at constant year-2018 levels.

```r
# --- Load data ---
# CMIP6 historical + SSP CO2 (annual, ppm)
url <- "https://files.isimip.org/ISIMIP2a/InputData/climate_co2/co2/historica
l_CO2_annual_1765_2018.txt"

df <- read_table(
  url,
  col_names = c("year", "co2")
)

##
## ── Column specification ────────────────────────────────────────────────
────────

## cols(
##   year = col_double(),
##   co2 = col_double()
## )

# # Mauna Loa
# url <- "https://gml.noaa.gov/webdata/ccgg/trends/co2/co2_annmean_mlo.txt"

# df <- read_table(
#   url,
#   comment = "#",
#   col_names = c("year", "mean", "uncertainty")
# ) %>%
#   rename(co2 = mean) %>%
#   select(year, co2)

# --- Parameters ---
beta <- 0.46
co2_init <- df$co2[1]
gpp_init <- 100

# --- Compute GPP ---
df <- df %>%
  mutate(gpp = gpp_init * (co2 / co2_init)^beta)

# --- Create 1000-year spin-up (repeat first row) ---
spinup <- df %>%
  slice(1) %>%
  slice(rep(1, 1000)) %>%
  mutate(year = (-999:0) + 1764)

# stabilisation ---------
co2_final <- df$co2[nrow(df)]
gpp_final <- df$gpp[nrow(df)]
year_final <- df$year[nrow(df)]
```

```
stabilization <- tibble(
  year = (year_final + 1):(year_final + 1000),
  co2  = co2_final,
  gpp  = gpp_final
)

# --- Combine spin-up + real data ---
df_full <- bind_rows(spinup, df, stabilization) %>%
  mutate(sim_year = row_number())  # simulation index
```

Run model

```
# run the model, it returns a data frame
df_ccascade <- df_full |>
  mutate(out = ccascade(gpp)) |>
  rename(realyear = year) |>
  unnest(out) |>
  mutate(cveg = cleaf + cwood + croot)
```

Estimate steady state

... from transient outputs.

```
k <- 10

df_ccascade <- df_ccascade |>
  mutate(
    dc = (cveg - lag(cveg, k)) / k,
    bp = gpp - ra
    ) |>
  mutate(c_star = estimate_steady_state(c_pool = cveg, c_in = bp, dc = dc))
```

The estimated steady-state approaches the true steady-state level much faster than transiently changing pools and therefore provides a reliable (albeit imperfect) estimate of the latter before the latter is known.

```
c_star_act <- df_ccascade |>
  tail(n = 1) |>
  pull(cveg) |>
  mean(na.rm = TRUE)

df_ccascade |>
  ggplot() +
  geom_line(aes(realyear, cveg, color = "Vegetation C pool")) +
  geom_line(aes(realyear, c_star, color = "Steady-state estimate")) +
  scale_color_okabeito(name = "") +
  theme_classic() +
  labs(x = "Simulation year", y = "Pool size (PgC)") +
  xlim(2018, 2500) + ylim(900, 1100) +
  geom_vline(xintercept = 2018, linetype = "dotted") +
  geom_hline(yintercept = c_star_act, linetype = "dotted")
```

```
## Warning: Removed 1771 rows containing missing values or values outside the
 scale range
## (`geom_line()`).
## Removed 1771 rows containing missing values or values outside the scale ra
nge
## (`geom_line()`).
```

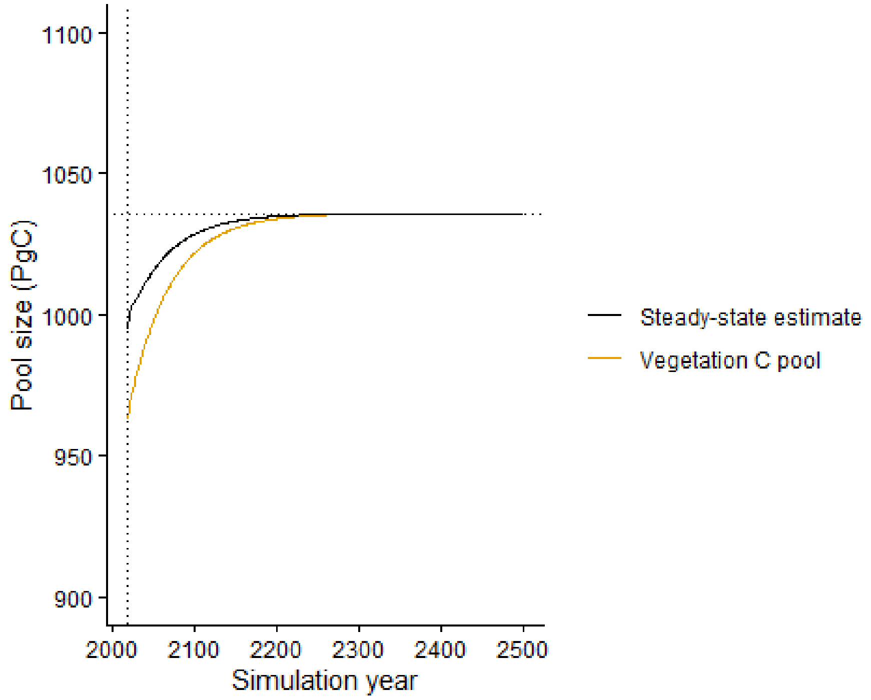

Figure S31 *Simulated vegetation C pool size (orange line) in response to transiently changing CO2 after 1700 up to 2018 and the estimated steady state pool size, derived from transiently changing simulation outputs (black line). CO2 is held constant for 1000 years after 2018.*

The error of the steady state estimate is on the order of a 16-0 % percent of the absolute steady-state pool size and approaches zero towards the end of the transient simulation. The time scale to approach zero depends on the turnover rates of the respective pool(s) (not shown).

```
df_ccascade <- df_ccascade |>
  mutate(
    diff_cveg = cveg - c_star_act,
    diff_cstar = c_star - c_star_act
    ) |>
  mutate(
    reldiff_cveg = 100 * diff_cveg / c_star_act,
    reldiff_cstar = 100 * diff_cstar / c_star_act
    )

df_ccascade |>
```

```
  ggplot() +
  geom_line(aes(realyear, diff_cstar)) +
  theme_classic() +
  labs(x = "Simulation year", y = "Error of estimate (PgC)") +
  xlim(2018, 2500) + ylim (-50, 2) +
  geom_vline(xintercept = 2018, linetype = "dotted") +
  geom_hline(yintercept = 0, linetype = "dotted")

## Warning: Removed 1771 rows containing missing values or values outside the
 scale range
## (`geom_line()`).
```

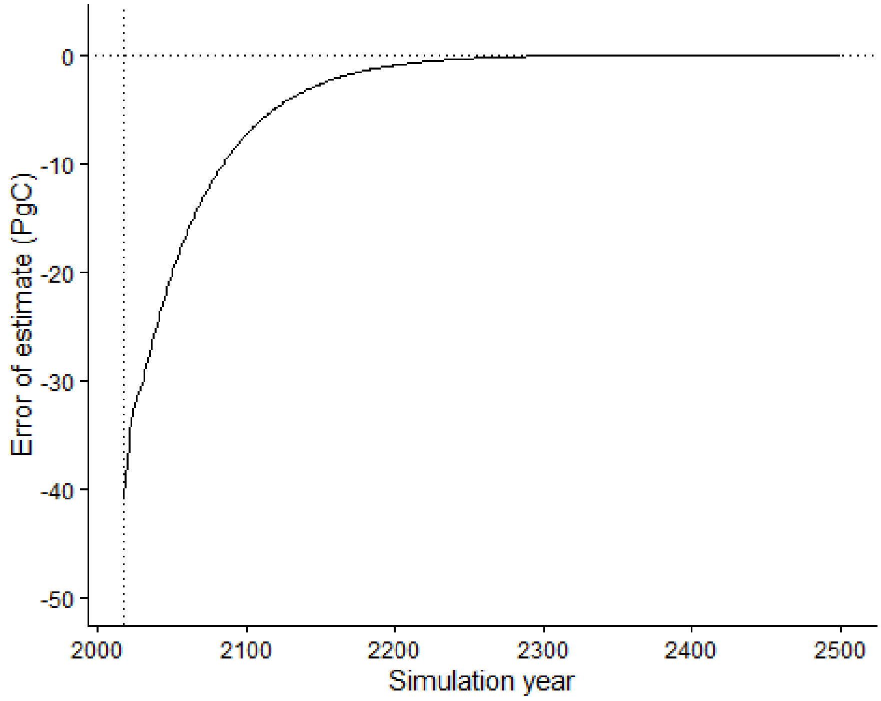

Figure S32 *Difference between the actual steady state vegetation C pool and the estimated value, derived from transient model outputs.*

```
df_ccascade |>
  ggplot() +
  geom_line(aes(realyear, reldiff_cstar)) +
  theme_classic() +
  labs(x = "Simulation year", y = "Relative error of estimate (%)") +
  xlim(2018, 2500) + ylim (-5, 0.5) +
  geom_vline(xintercept = 2018, linetype = "dotted") +
  geom_hline(yintercept = 0, linetype = "dotted")

## Warning: Removed 1771 rows containing missing values or values outside the
 scale range
## (`geom_line()`).
```

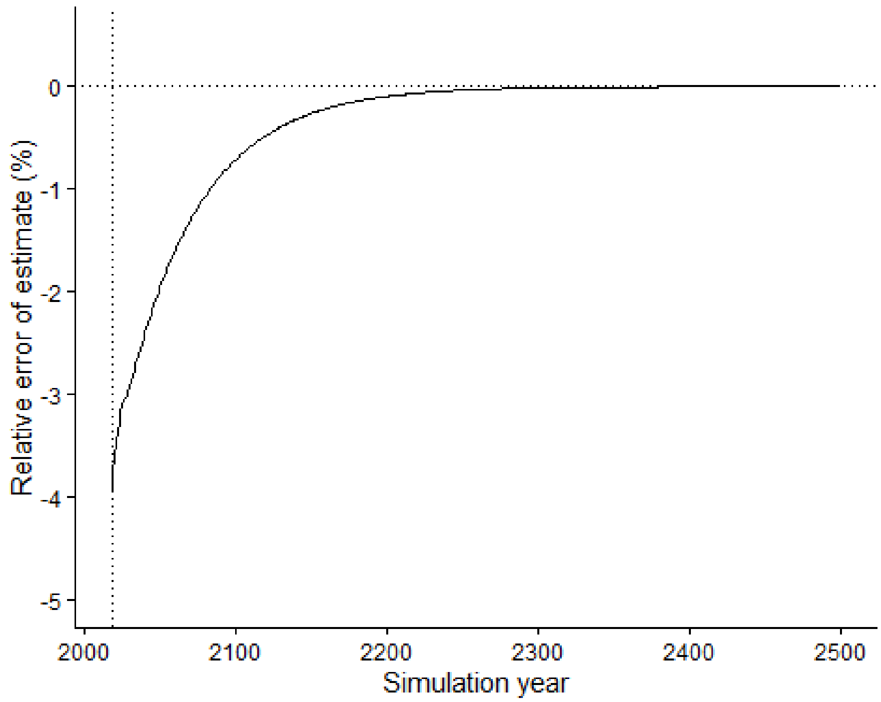

Figure S33 *Difference between the actual steady state vegetation C pool and the estimated value, derived from transient model outputs.*

Assess L

By design, the actual linearity term L diagnosed from the actual steady-state yields a value of $L = 1$. In contrast, when diagnosing it from uncorrected transient outputs, the value is below 0.7. L calculated from the estimated steady state yields a more accurate value–close to 1.0

```
df_lin <- df_ccascade |>
  filter(realyear %in% 1765:1769) |>
  summarise(cveg = mean(cveg), npp = mean(bp), c_star = mean(c_star)) |>
  mutate(time = 0) |>
  bind_rows(
    df_ccascade |>
      filter(realyear %in% 2014:2018) |>
      summarise(cveg = mean(cveg), npp = mean(bp), c_star = mean(c_star)) |>
      mutate(time = 1)
  ) |>
  bind_rows(
    df_ccascade |>
      tail(n = 5) |>
      summarise(cveg = mean(cveg), npp = mean(bp), c_star = mean(c_star)) |>
      mutate(time = 9)
  ) %>%
  pivot_wider(
    names_from = time,
```

```r
    values_from = c(cveg, npp, c_star),
    names_glue = "{.value}_{time}"
  ) |>
  mutate(
    lin_transient = ((cveg_1 - cveg_0)/cveg_0)/((npp_1 - npp_0)/npp_0),
    lin_ss_est = ((c_star_1 - c_star_0)/c_star_0)/((npp_1 - npp_0)/npp_0),
    lin_ss_act = ((c_star_9 - c_star_0)/c_star_0)/((npp_9 - npp_0)/npp_0),
  )

df_lin |>
  select(lin_transient, lin_ss_est, lin_ss_act) |>
  kable()
```

lin_transient	lin_ss_est	lin_ss_act
0.5623136	0.7558109	1.001482

```r
df_lin |>
  select(starts_with("lin_")) |>
  pivot_longer(cols = everything(), names_to = "source", values_to = "lin") |
>
  ggplot(aes(x = 1, y = lin, color = source)) +
  geom_point(size = 3) +
  scale_color_okabeito() +
  theme_classic() +
  geom_hline(yintercept = 1, linetype = "dotted") +
  labs(y = "L (unitless)")+ theme(
  axis.title.x = element_blank(),
  axis.text.x  = element_blank(),
  axis.ticks.x = element_blank()
)
```

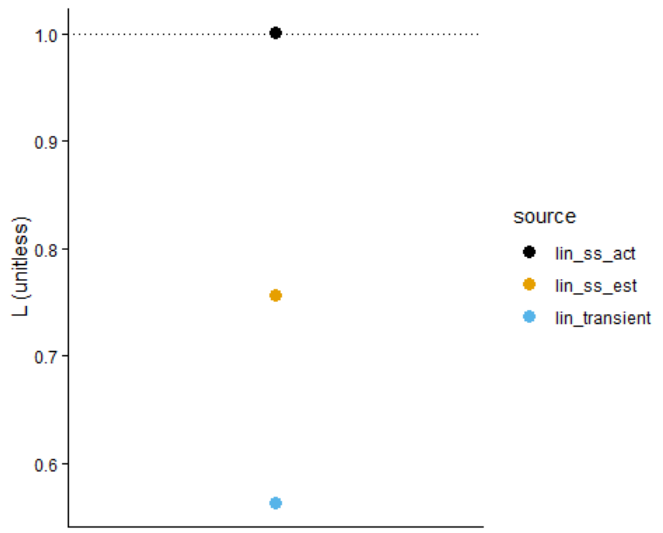

Figure S34 *Linearity term L derived for the linear model (ccascade) from (uncorrected) transient outputs (blue), from the estimated steady-state (orange), and from the actual steady state (black).*

Non-linear model

This is a modified version of the `ccascade` model where the woody C pool turnover rate increases with the size of the woody C pool.

Define model

Define the model as before, but with a modification

```
ccascade <- function(
  gpp,
  state = initpools(),
  par = getpar(),
  fluxes = initfluxes(),
  nonlinear = FALSE
  ){

  # determine integration length (number of time steps) from length of 'c_inf
lux'
  len <- length(gpp)

  # output the state and fluxes in one data frame
  df <- dplyr::tibble()

  # integrate over each time step (this is an implementation of the different
ial equation)
  for (yr in seq(len)){

    # update states and fluxes
```

```r
    if (nonlinear){
      out <- ccascade_onestep_nonlinear(gpp[yr], state, par, fluxes)
    } else {
      out <- ccascade_onestep(gpp[yr], state, par, fluxes)
    }
    state <- out$state
    fluxes <- out$fluxes

    # record for output
    df <- dplyr::bind_rows(
      df,
      dplyr::bind_cols(
        dplyr::tibble(year = yr),
        dplyr::as_tibble(state),
        dplyr::as_tibble(fluxes))
      )
  }
  return(df)
}

ccascade_onestep_nonlinear <- function(gpp, state, par, fluxes){

  # total biomass production
  bp <- gpp * par$bpe
  fluxes$ra <- gpp * (1 - par$bpe)

  kwood_eff <- par$kwood *
    ifelse(
    state$cwood < 800,
    par$kwood,
    par$kwood * (state$cwood/800) ^ 1.0
    )

  # biomass turnover by pool, use exponential here to avoid numerical instabi
lity with k -> 1
  dleaf <- (1 - exp(-par$kleaf)) * state$cleaf
  dwood <- (1 - exp(-kwood_eff)) * state$cwood
  droot <- (1 - exp(-par$kroot)) * state$croot

  # update biomass pools
  state$cleaf <- state$cleaf + bp * par$fleaf - dleaf
  state$cwood <- state$cwood + bp * par$fwood - dwood
  state$croot <- state$croot + bp * par$froot - droot

  # litter turnover
  dflitt <- (1 - exp(-par$kflitt)) * state$flitt
  dslitt <- (1 - exp(-par$kslitt)) * state$slitt

  # update litter: add biomass turnover and update litter pools
```

```r
  state$flitt <- state$flitt + dleaf + droot - dflitt
  state$slitt <- state$slitt + dwood         - dslitt

  # heterotrophic respiration during litter decomposition
  rh_litt <- (1 - par$eff) * (dflitt + dslitt)

  # soil turnover
  dfsoil <- (1 - exp(-par$kfsoil)) * state$fsoil
  dssoil <- (1 - exp(-par$kssoil)) * state$ssoil

  # update soil: remainder of litter turnover goes to soil, subtract decompos
ing portion
  state$fsoil <- state$fsoil + par$eff * par$ffast       * (dflitt + dslitt)
- dfsoil
  state$ssoil <- state$ssoil + par$eff * (1 - par$ffast) * (dflitt + dslitt)
- dssoil

  # heterotrophic respiration from soil decomposition
  rh_soil <- dfsoil + dssoil
  fluxes$rh <- rh_litt + rh_soil

  return(list(state = state, fluxes = fluxes))
}
```

Run model

```r
# run the model, it returns a data frame
df_ccascade_nonlin <- df_full |>
  mutate(out = ccascade(gpp, nonlinear = TRUE)) |>
  rename(realyear = year) |>
  unnest(out) |>
  mutate(cveg = cleaf + cwood + croot)
```

Estimate steady state

… from transient outputs.

```r
k <- 10

df_ccascade_nonlin <- df_ccascade_nonlin |>
  mutate(
    dc = (cveg - lag(cveg, k)) / k,
    bp = gpp - ra
    ) |>
  mutate(c_star = estimate_steady_state(c_pool = cveg, c_in = bp, dc = dc))
```

The estimated steady-state approaches the true steady-state level much faster than transiently changing pools and therefore provides a reliable (albeit imperfect) estimate of the latter before the latter is known.

```
c_star_act <- df_ccascade_nonlin |>
  tail(n = 1) |>
  pull(cveg) |>
  mean(na.rm = TRUE)

df_ccascade_nonlin |>
  ggplot() +
  geom_line(aes(realyear, cveg, color = "Vegetation C pool")) +
  geom_line(aes(realyear, c_star, color = "Steady-state estimate")) +
  scale_color_okabeito(name = "") +
  theme_classic() +
  labs(x = "Simulation year", y = "Pool size (PgC)") +
  xlim(2018, 2500) +
  ylim(5500, 6500) +
  geom_vline(xintercept = 2018, linetype = "dotted") +
  geom_hline(yintercept = c_star_act, linetype = "dotted")

## Warning: Removed 1771 rows containing missing values or values outside the
 scale range
## (`geom_line()`).
## Removed 1771 rows containing missing values or values outside the scale ra
nge
## (`geom_line()`).
```

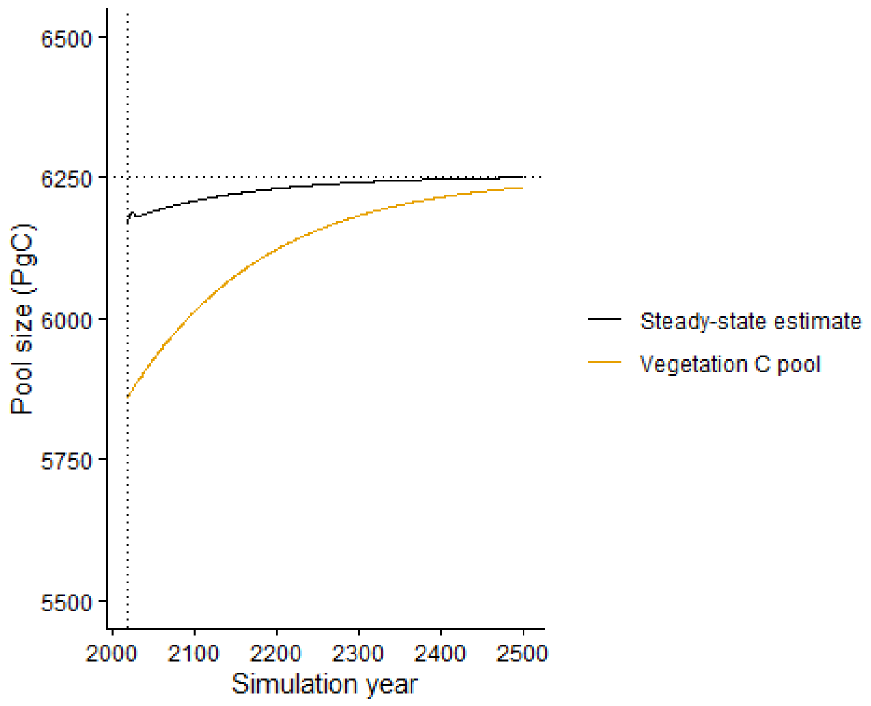

Figure S35 *Simulated vegetation C pool size (orange line) in response to transiently changing CO2 after 1700 up to 2018 and the estimated steady state pool size, derived from transiently changing simulation outputs (black line). CO2 is held constant for 1000 years after 2018.*

The error of the steady state estimate is on the order of a 16-0 % percent of the absolute steady-state pool size and approaches zero towards the end of the transient simulation. The time scale to approach zero depends on the turnover rates of the respective pool(s) (not shown).

```
df_ccascade_nonlin <- df_ccascade_nonlin |>
  mutate(
    diff_cveg = cveg - c_star_act,
    diff_cstar = c_star - c_star_act
    ) |>
  mutate(
    reldiff_cveg = 100 * diff_cveg / c_star_act,
    reldiff_cstar = 100 * diff_cstar / c_star_act
    )

df_ccascade_nonlin |>
  ggplot() +
  geom_line(aes(realyear, diff_cstar)) +
  theme_classic() +
  labs(x = "Simulation year", y = "Error of estimate (PgC)") +
  xlim(2018, 2500) +
  ylim (-100, 100) +
  geom_vline(xintercept = 2018, linetype = "dotted") +
  geom_hline(yintercept = 0, linetype = "dotted")

## Warning: Removed 1771 rows containing missing values or values outside the
 scale range
## (`geom_line()`).
```

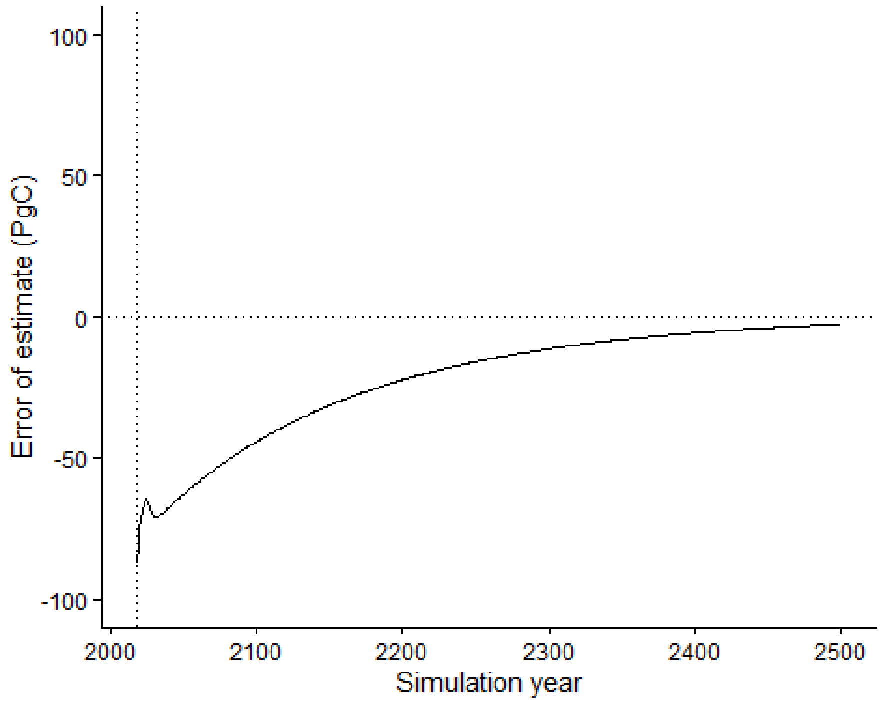

Figure S36 *Difference between the actual steady state vegetation C pool and the estimated value, derived from transient model outputs.*

```
df_ccascade_nonlin |>
  ggplot() +
  geom_line(aes(realyear, reldiff_cstar)) +
  theme_classic() +
  labs(x = "Simulation year", y = "Relative error of estimate (%)") +
  xlim(2018, 2500) +
  ylim (-2, 1) +
  geom_vline(xintercept = 2018, linetype = "dotted") +
  geom_hline(yintercept = 0, linetype = "dotted")

## Warning: Removed 1771 rows containing missing values or values outside the
 scale range
## (`geom_line()`).
```

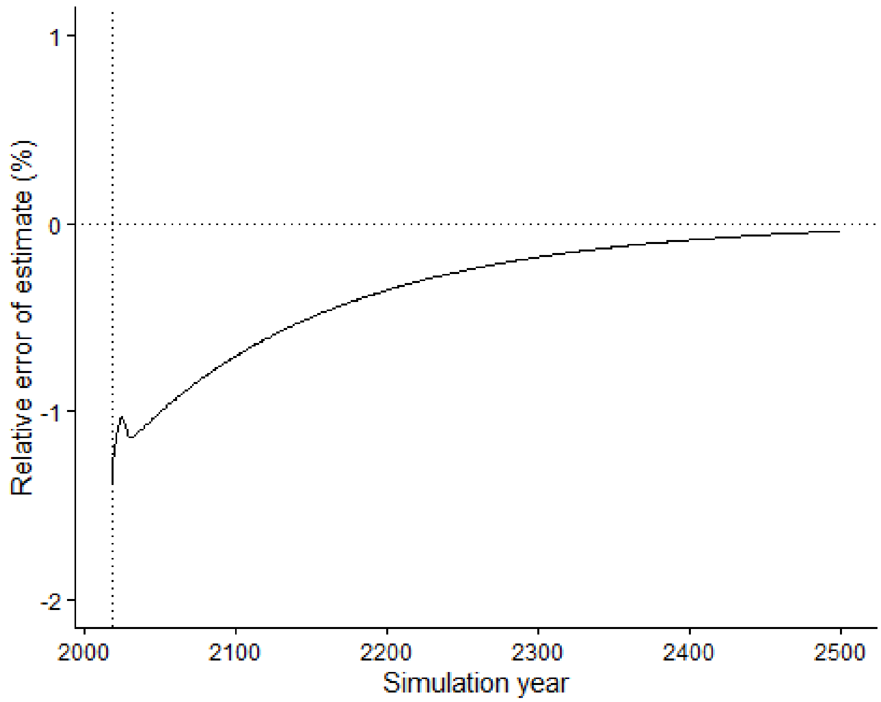

Figure S37 *Difference between the actual steady state vegetation C pool and the estimated value, derived from transient model outputs.*

Assess L

By design, the actual linearity term L diagnosed from the actual steady-state yields a value of $L = 1$. In contrast, when diagnosing it from uncorrected transient outputs, the value is below 0.7. L calculated from the estimated steady state yields a more accurate value– close to 1.0

```
df_lin <- df_ccascade_nonlin |>
  filter(realyear %in% 1765:1769) |>
  summarise(cveg = mean(cveg), npp = mean(bp), c_star = mean(c_star)) |>
  mutate(time = 0) |>
  bind_rows(
    df_ccascade_nonlin |>
      filter(realyear %in% 2014:2018) |>
      summarise(cveg = mean(cveg), npp = mean(bp), c_star = mean(c_star)) |>
      mutate(time = 1)
  ) |>
  bind_rows(
    df_ccascade_nonlin |>
      tail(n = 5) |>
      summarise(cveg = mean(cveg), npp = mean(bp), c_star = mean(c_star)) |>
      mutate(time = 9)
  ) %>%
  pivot_wider(
    names_from = time,
```

```r
    values_from = c(cveg, npp, c_star),
    names_glue = "{.value}_{time}"
  ) |>
  mutate(
    lin_transient = ((cveg_1 - cveg_0)/cveg_0)/((npp_1 - npp_0)/npp_0),
    lin_ss_est = ((c_star_1 - c_star_0)/c_star_0)/((npp_1 - npp_0)/npp_0),
    lin_ss_act = ((c_star_9 - c_star_0)/c_star_0)/((npp_9 - npp_0)/npp_0),
  )

df_lin |>
  select(lin_transient, lin_ss_est, lin_ss_act) |>
  kable()
```

lin_transient	lin_ss_est	lin_ss_act
0.16124	0.4121651	0.4910464

```r
df_lin |>
  select(starts_with("lin_")) |>
  pivot_longer(cols = everything(), names_to = "source", values_to = "lin") |
>
  ggplot(aes(x = 1, y = lin, color = source)) +
  geom_point(size = 3) +
  scale_color_okabeito() +
  theme_classic() +
  geom_hline(yintercept = 1, linetype = "dotted") +
  labs(y = "L (unitless)")+ theme(
  axis.title.x = element_blank(),
  axis.text.x  = element_blank(),
  axis.ticks.x = element_blank()
)
```

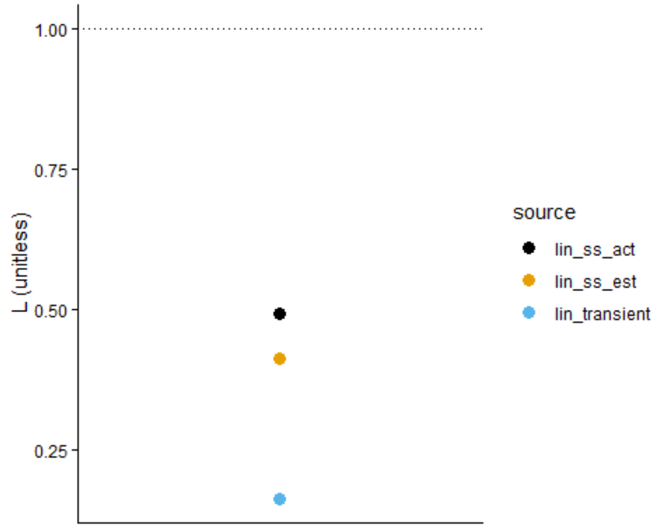

Figure S38 *Linearity term L derived for the linear model (ccascade) from (uncorrected) transient outputs (blue), from the estimated steady-state (orange), and from the actual steady state (black).*

BiomeEP

This performs the same analysis with a vegetation demography model, BiomeEP.

Create forcing

```
# expand to daily
co2_daily <- df %>%
  mutate(date = map(year, ~ seq.Date(
    from = as.Date(paste0(.x, "-01-01")),
    to   = as.Date(paste0(.x, "-12-31")),
    by   = "day"
  ))) %>%
  unnest(date) %>%
  select(date, co2)

# extract BiomeEP forcing
forcing <- biomee_p_model_drivers$forcing[[1]]

# align years
forcing_full <- forcing %>%
  mutate(doy = yday(date)) %>%
  select(-date)

years <- df$year

forcing_expanded <- map_dfr(years, function(y) {
  forcing_full %>%
    mutate(
      year = y,
```

```r
      date = as.Date(doy - 1, origin = paste0(y, "-01-01"))
    )
})

# merge CO2 forcing
forcing_final <- forcing_expanded %>%
  select(-co2) |>
  left_join(co2_daily, by = "date")
```

Add 1000 year stabilisation phase in forcing.

```r
last_year <- max(lubridate::year(forcing_final$date))

forcing_last <- forcing_final %>%
  filter(lubridate::year(date) == last_year) %>%
  mutate(doy = lubridate::yday(date)) %>%
  select(-date)

co2_final <- forcing_final %>%
  filter(lubridate::year(date) == last_year) %>%
  summarise(co2 = mean(co2, na.rm = TRUE)) %>%
  pull(co2)

years_ext <- (last_year + 1):(last_year + 1000)

forcing_stabilization <- purrr::map_dfr(years_ext, function(y) {
  forcing_last %>%
    mutate(
      year = y,
      date = as.Date(doy - 1, origin = paste0(y, "-01-01")),
      co2 = co2_final
    )
})

forcing_full <- bind_rows(
  forcing_final,
  forcing_stabilization
)

biomee_p_model_drivers$forcing[[1]] <- forcing_full
```

Run model

Some simulation parameters: 1000 years spinup (repeating first year)

```r
biomee_p_model_drivers$params_siml[[1]]$spinupyears <- 1000
biomee_p_model_drivers$params_siml[[1]]$recycle <- 1
biomee_p_model_drivers$params_siml[[1]]$nyeartrend <- 2018 - 1765 + 1000
```

```r
out <- runread_biomee_f(
  biomee_p_model_drivers,
  makecheck = TRUE,
  parallel = FALSE
  )
```

```
## Warning: There was 1 warning in `dplyr::mutate()`.
## ℹ In argument: `data = purrr::pmap(., run_biomee_f_bysite, makecheck =
##   makecheck)`.
## Caused by warning in `build_params_siml()`:
## ! Info: provided value of nyeartrend is less than the number of years of f
orcing data (1254). Only the first 1253 will be used.
```

```r
df_biomeep <- out$data[[1]]$output_annual_tile |>
  as_tibble() |>
  select(year, npp = NPP, cveg = plantC) |>
  mutate(realyear = year + 764)
```

Smooth to avoid oscillations

```r
# determine frequency
spec <- spectrum(
  df_biomeep |>
    filter(realyear %in% 2018:3000) |>
    pull(cveg),
  plot = FALSE
  )

freq <- spec$freq[which.max(spec$spec)]
period <- 1 / freq

vars <- c("npp", "cveg")

win <- ceiling(period)

df_biomeep_smooth <- df_biomeep %>%
  mutate(
    across(
      all_of(vars),
      ~ slide_dbl(.x, mean, .after = win - 1, .complete = TRUE),
      .names = "{.col}"
    )
  ) %>%
  slice(1:(n() - win + 1))
```

Estimate steady state.

```r
k <- 10
df_biomeep_smooth <- df_biomeep_smooth |>
  mutate(
```

```
    dc = (cveg - lag(cveg, k)) / k
    ) |>
  mutate(c_star = estimate_steady_state(c_pool = cveg, c_in = npp, dc = dc))
```

Evaluate

```
c_star_act <- df_biomeep_smooth |>
  tail(n = 5) |>
  pull(cveg) |>
  mean(na.rm = TRUE)

df_biomeep_smooth |>
  ggplot() +
  geom_line(aes(realyear, cveg, color = "Vegetation C pool")) +
  geom_line(aes(realyear, c_star, color = "Steady-state estimate")) +
  khroma::scale_color_okabeito(name = "") +
  theme_classic() +
  labs(x = "Simulation year", y = "Pool size (kg C)") +
  xlim(1980, 2500) +
  ylim(11, 14) +
  geom_vline(xintercept = 2018, linetype = "dotted") +
  geom_hline(yintercept = c_star_act, linetype = "dotted")

## Warning: Removed 1680 rows containing missing values or values outside the
 scale range
## (`geom_line()`).
## Removed 1680 rows containing missing values or values outside the scale ra
nge
## (`geom_line()`).
```

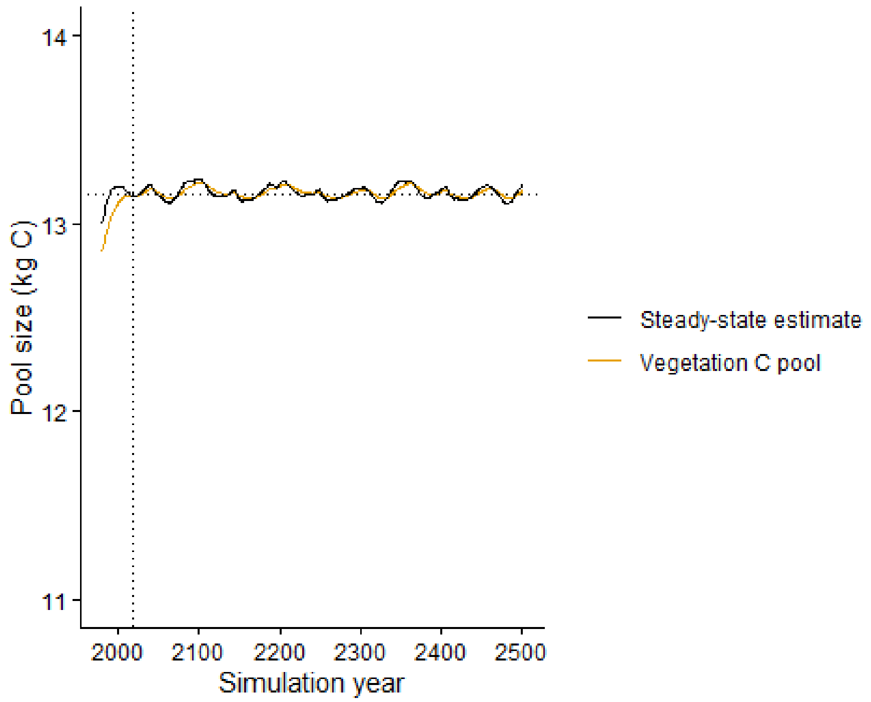

Figure S39 *Simulated vegetation C pool size (orange line) in response to transiently changing CO2 after 1700 up to 2018 and the estimated steady state pool size, derived from transiently changing simulation outputs (black line). CO2 is held constant for 1000 years after 2018.*

The error of the steady state estimate is on the order of a 16-0 % percent of the absolute steady-state pool size and approaches zero towards the end of the transient simulation. The time scale to approach zero depends on the turnover rates of the respective pool(s) (not shown).

```
df_biomeep_smooth <- df_biomeep_smooth |>
  mutate(
    diff_cveg = cveg - c_star_act,
    diff_cstar = c_star - c_star_act
    ) |>
  mutate(
    reldiff_cveg = 100 * diff_cveg / c_star_act,
    reldiff_cstar = 100 * diff_cstar / c_star_act
    )

df_biomeep_smooth  |>
  ggplot() +
  geom_line(aes(realyear, diff_cstar)) +
  theme_classic() +
  labs(x = "Simulation year", y = "Error of estimate (kg C)") +
  xlim(2018, 2500) +
  ylim (-1, 0.5) +
```

```
  geom_vline(xintercept = 2018, linetype = "dotted") +
  geom_hline(yintercept = 0, linetype = "dotted")

## Warning: Removed 1718 rows containing missing values or values outside the
 scale range
## (`geom_line()`).
```

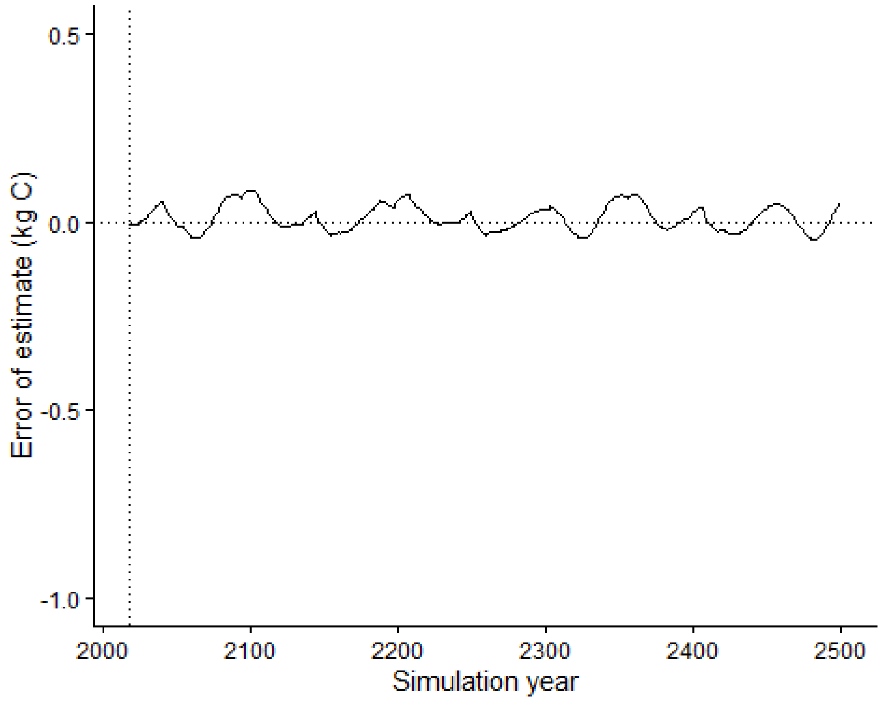

Figure S40 *Difference between the actual steady state vegetation C pool and the estimated value, derived from transient model outputs.*

```
df_biomeep_smooth |>
  ggplot() +
  geom_line(aes(realyear, reldiff_cstar)) +
  theme_classic() +
  labs(x = "Simulation year", y = "Relative error of estimate (%)") +
  xlim(2018, 2500) +
  ylim (-5, 2) +
  geom_vline(xintercept = 2018, linetype = "dotted") +
  geom_hline(yintercept = 0, linetype = "dotted")

## Warning: Removed 1718 rows containing missing values or values outside the
 scale range
## (`geom_line()`).
```

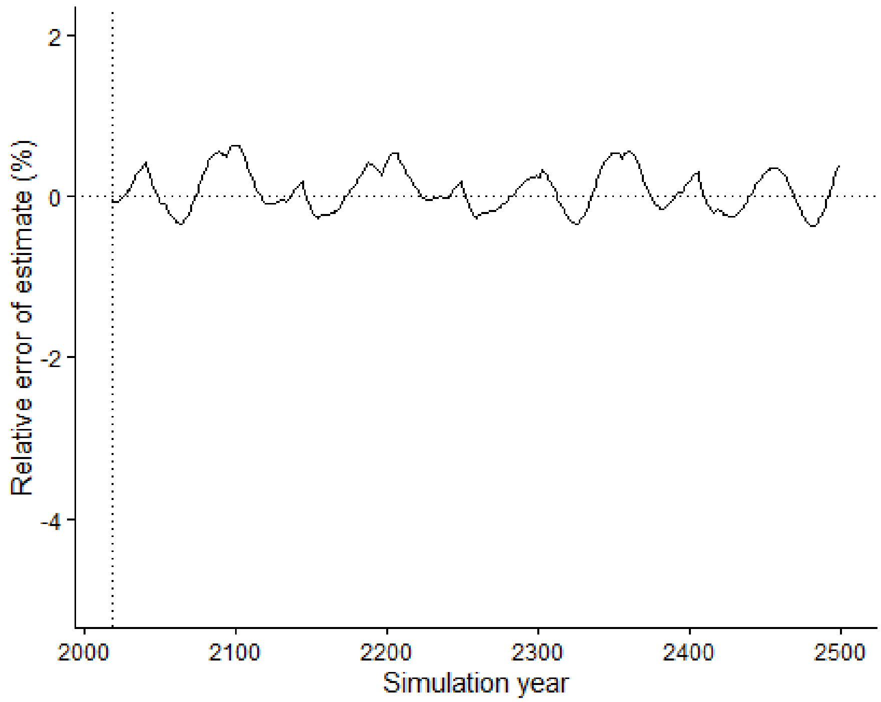

Figure S41 *Difference between the actual steady state vegetation C pool and the estimated value, derived from transient model outputs.*

Assess L

By design, the actual linearity term L diagnosed from the actual steady-state yields a value of $L = 1$. In contrast, when diagnosing it from uncorrected transient outputs, the value is below 0.7. L calculated from the estimated steady state yields a more accurate value–close to 1.0

```
df_lin <- df_biomeep_smooth |>
  # filter(realyear %in% 1765:(1765+ceiling(period))) |>
  filter(realyear %in% 1765:1769) |>
  summarise(cveg = mean(cveg), npp = mean(npp), c_star = mean(c_star)) |>
  mutate(time = 0) |>
  bind_rows(
    df_biomeep_smooth |>
      # filter(realyear %in% (2018-ceiling(period)):2018) |>
      filter(realyear %in% 2014:2018) |>
      summarise(cveg = mean(cveg), npp = mean(npp), c_star = mean(c_star)) |>

      mutate(time = 1)
  ) |>
  bind_rows(
    df_biomeep_smooth |>
      # tail(n = ceiling(period)) |>
      tail(n = 5) |>
      summarise(cveg = mean(cveg), npp = mean(npp), c_star = mean(c_star)) |>
```

```r
    mutate(time = 9)
  ) %>%
  pivot_wider(
    names_from = time,
    values_from = c(cveg, npp, c_star),
    names_glue = "{.value}_{time}"
  ) |>
  mutate(
    lin_transient = ((cveg_1 - cveg_0)/cveg_0)/((npp_1 - npp_0)/npp_0),
    lin_ss_est = ((c_star_1 - c_star_0)/c_star_0)/((npp_1 - npp_0)/npp_0),
    lin_ss_act = ((c_star_9 - c_star_0)/c_star_0)/((npp_9 - npp_0)/npp_0),
  )

df_lin |>
  select(lin_transient, lin_ss_est, lin_ss_act) |>
  kable()
```

lin_transient	lin_ss_est	lin_ss_act
0.8009264	0.8188336	0.827601

```r
df_lin |>
  select(starts_with("lin_")) |>
  pivot_longer(cols = everything(), names_to = "source", values_to = "lin") |
>
  ggplot(aes(x = 1, y = lin, color = source)) +
  geom_point(size = 3) +
  scale_color_okabeito() +
  theme_classic() +
  geom_hline(yintercept = 1, linetype = "dotted") +
  labs(y = "L (unitless)")+ theme(
  axis.title.x = element_blank(),
  axis.text.x  = element_blank(),
  axis.ticks.x = element_blank()
)
```

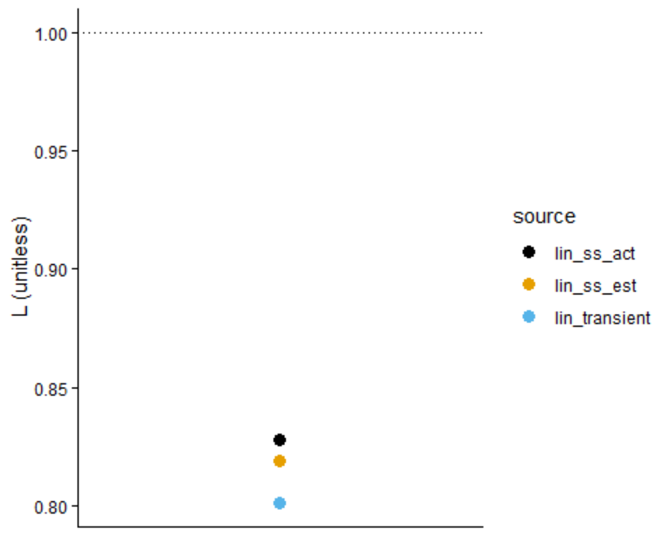

Figure S42 *Linearity term L derived for the non-linear model (BiomeEP) from (uncorrected) transient outputs (blue), from the estimated steady-state (orange), and from the actual steady state (black).*

Summary

The result of the steady-state estimation, made for year 2018, is summarised below.

Linear model

```
# linear model (ccascade)
summ_ccascade <- df_ccascade |>
  filter(realyear == 2018) |>
  dplyr::select(realyear, cveg, c_star, diff_cveg, diff_cstar, reldiff_cveg,
reldiff_cstar)

summ_ccascade |>
  kable()
```

realyear	cveg	c_star	diff_cveg	diff_cstar	reldiff_cveg	reldiff_cstar
2018	962.708<br>9	994.899<br>6	-73.23049	-41.03984	-7.068993	-3.961606

- Vegetation C pool is {r} `format(abs(summ_ccascade$reldiff_cveg[1]), digits = 2)` below its steady-state.
- The estimated steady-state vegetation C pool is {r} `format(abs(summ_ccascade$reldiff_cstar[1]), digits = 2)`% below its actual steady-state.

Non-linear model

```
# non-linear model (ccascade)
summ_ccascade_nonlin <- df_ccascade_nonlin |>
```

```
  filter(realyear == 2018) |>
  dplyr::select(realyear, cveg, c_star, diff_cveg, diff_cstar, reldiff_cveg,
reldiff_cstar)

summ_ccascade_nonlin |>
  kable()
```

realyear	cveg	c_star	diff_cveg	diff_cstar	reldiff_cveg	reldiff_cstar
2018	5857.814	6165.579	-395.1043	-87.33884	-6.318718	-1.396769

- Vegetation C pool is {r} `format(abs(summ_ccascade_nonlin$reldiff_cveg[1]), digits = 2)`% below its steady-state.
- The estimated steady-state vegetation C pool is {r} `format(abs(summ_ccascade_nonlin$reldiff_cstar[1]), digits = 2)`% below its actual steady-state.

Non-linear model (BiomeEP)

```
# non-linear model (BiomeEP)
summm_biomeep <- df_biomeep_smooth |>
  filter(realyear == 2018) |>
  dplyr::select(realyear, cveg, c_star, diff_cveg, diff_cstar, reldiff_cveg,
reldiff_cstar)

summm_biomeep |>
  kable()
```

realyear	cveg	c_star	diff_cveg	diff_cstar	reldiff_cveg	reldiff_cstar
2018	13.1428	13.14562	-0.0108274	-0.0080131	-0.0823152	-0.0609194

- Vegetation C pool is {r} `format(abs(summm_biomeep$reldiff_cveg[1]), digits = 2)`% below its steady-state.
- The estimated steady-state vegetation C pool is {r} `format(abs(summm_biomeep$reldiff_cstar[1]), digits = 2)`% below its actual steady-state.
